# Analytical Size Exclusion Chromatography Coupled with Mass Spectrometry in Parallel with High-Throughput Venomics and Bioassaying for Venom Profiling

**DOI:** 10.3390/toxins15090552

**Published:** 2023-09-05

**Authors:** Sedef Terzioglu, Mátyás A. Bittenbinder, Julien Slagboom, Bas van de Velde, Nicholas R. Casewell, Jeroen Kool

**Affiliations:** 1 Division of Bioanalytical Chemistry, Department of Chemistry and Pharmaceutical Sciences, Faculty of Sciences, Amsterdam Institute of Molecular and Life Sciences (AIMMS), Vrije Universiteit Amsterdam, 1081 HZ Amsterdam, The Netherlands; 2 Naturalis Biodiversity Center, 2333 CR Leiden, The Netherlands; 3 Centre for Analytical Sciences Amsterdam (CASA), 1098 XH Amsterdam, The Netherlands; 4 Centre for Snakebite Research & Interventions, Liverpool School of Tropical Medicine, Liverpool L3 5QA, UK

**Keywords:** snakebite, coagulopathy, size exclusion chromatography, mass spectrometry, snake venomics, *Daboia russelii*, *Bungarus multicinctus*

## Abstract

Modern analytical size exclusion chromatography (SEC) is a suitable technique to separate venom toxin families according to their size characteristics. In this study, a method was developed to separate intact venom toxins from *Bungarus multicinctus* and *Daboia russelii* venoms via analytical SEC using volatile, non-salt-containing eluents for post-column mass spectrometry, coagulation bioassaying and high-throughput venomics. Two venoms were used to demonstrate the method developed. While the venom of *Bungaurs multicinctus* is known to exert anticoagulant effects on plasma, in this study, we showed the existence of both procoagulant toxins and anticoagulant toxins. For *Daboia russelii* venom, the method revealed characteristic procoagulant effects, with a 90 kDa mass toxin detected and matched with the Factor X-activating procoagulant heterotrimeric glycoprotein named RVV-X. The strong procoagulant effects for this toxin show that it was most likely eluted from size exclusion chromatography non-denatured. In conclusion, the separation of snake venom by size gave the opportunity to separate some specific toxin families from each other non-denatured, test these for functional bioactivities, detect the eluting mass on-line via mass spectrometry and identify the eluted toxins using high-throughput venomics.

## 1. Introduction

Snakebite envenoming is considered as a neglected tropical disease mainly affecting those living in (sub-)tropical countries in the developing world [1]. Due to the low dose efficacy of currently used antivenoms, therapeutic improvements for snakebite are urgently required [1]. In addition, snake venoms can be valuable sources of compounds with potential medical relevance, due to their bioactive potencies and selectivity toward targets that are commonly of relevance to mammalian physiology [2]. For both reasons, understanding the mode of action of venoms, investigating their toxin composition, and gaining knowledge about their bioactivities is important. Antivenom developed for one species will most likely not have potent efficacy as a treatment for bites caused by other species due to inter-specific variations in toxin composition [3,4]. Even at the intraspecies level, variation in venom composition is commonplace between different populations of the same species [5]. In recent years, modern analytical tools have enabled comprehensive characterisations of venom composition, enabling the field to gain considerable insights into the key toxins that contribute to venom-induced pathology [6].

The two most medically relevant snake families are the Elapidae (elapids) and Viperidae (vipers) [1]. Both of these front-fanged snake families contain toxins in their venom that are members of several different protein families; these include three-finger toxins (3FTx), phospholipase A_2_s (PLA_2_s), snake C-type lectins (snaclecs), snake venom metalloproteinases (SVMPs), and snake venom serine proteinases (SVSPs) [7,8,9,10], with the first two types most commonly abundant in elapid venoms and the latter four in viper venoms. Crude snake venoms on average hold around 30 to more than 100 different toxins, which provides considerable scope for variation [11].

To determine the individual activities of specific toxins, separation of the crude venom followed by bioactivity measurements is required. In many studies, separation is performed by reversed-phase high-pressure liquid chromatography (RP-HPLC or RPLC) using a common C_18_ column and a gradient with an acidic-organic mobile phase [12]. This separation method, however, has the potential to denature some venom toxins due to the use of high concentrations of acidic and/or organic eluents, especially where the larger toxins are difficult to separate in their native form [13,14,15]. Denatured toxins that have lost their bioactivity in these situations represent false negatives in downstream bioassay testing. In the case of snake venom toxins, the toxin families SVMPs and SVSPs are considered to belong to the larger toxin category. Their sizes vary between 20 and 100 kDa and 26 and 67 kDa, respectively [9,10].

Since the total mass of toxins within one family falls in a specific mass range, for example, 3FTxs are between 5 and 8 kDa or PLA_2_s are between 12 and 15 kDa, separation by size-based chromatography provides a useful separation tool in venom research [16]. In conventional size exclusion chromatography (SEC), however, high sample amounts are usually demanded due to the large column size. These methods also make use of a mobile phase containing high concentrations of non-volatile buffer salts and therefore are not compatible with many post-column bioassays and also not with mass spectrometry (MS) [17,18]. By means of proteomics approaches in venom research (i.e., venomics), venom toxins can be characterised in their denatured form [19]. However, to be able to study them in parallel with bioactivity testing, other methods (that do not disrupt the toxin’s structure) are required. Several analytical SEC columns are compatible with water-based eluents containing low concentrations of organic solvents. Such columns have been used previously for snake venom separations, but using non-volatile buffer salts in the eluent [20].

The objective of this study was to develop an analytical technique that could be used to separate and fractionate snake venoms by toxin family in their native and/or biologically active state using SEC coupled with MS, followed by bioassaying and high-throughput venomics (HT-venomics [21]) characterisation. To minimize the denaturation and still be able to perform on-line MS, low concentrations of volatile organic acidic buffers had to be used for SEC separation. Since SEC is only a size-based separation, HT-venomics has been of high importance in distinguishing between co-eluting toxins. The focus of bioactivity testing here was to assess the pro- and anti-coagulant effects using a post-column implemented high-throughput screening (HTS) coagulation assay via nanofractionation analytics [22]. For the coagulation bioassay, the separated venom toxins were fractionated into 384-well plates followed by bioassaying from which so-called bioactivity chromatograms could be constructed. These could then be correlated with the parallel acquired SEC-UV, SEC-MS, and SEC-HT-venomics data. Since coagulopathy was the biological activity measured in this research, two venoms that include toxins previously demonstrated to cause distinct modulation of coagulation were selected to establish, develop and validate the analytical methodology from the many-banded krait (*Bungarus multicinctus*) and the Russell’s viper (*Daboia russelii*) [23,24].

## 2. Results and Discussion

The first part of this study consisted of the optimisation of the SEC-separation approach, with a core focus on exploring the impact that differences in the mobile phase conditions had on separation (Section 2.1). Next, several optimal mobile phase conditions were selected and implemented to separate and fractionate the venom of *D. russelii* and *B. multicinctus* onto 384-well plates, which were then used for HT-venomics and bioassays. SEC-MS was performed post-column in an on-line fashion. The results of the HT-venomics, bioassays, and SEC-MS analysis are displayed and discussed in Section 2.2.

### 2.1. SEC Optimisation

Optimisation of the SEC separations was performed using the protein mix (PM) and venom from *B. multicinctus* and *D. russelii*. The optimisation steps are elaborated on in the Supporting Information (SI) document in Section S3 and involve differences in the flow rate (Section S3.1), injection volume (Section S3.2), concentration of venom and/or PM (Section S3.2), mobile phase composition tested with a PM (Section S3.3), and mobile phase composition tested with venom (Section S3.4). The PM was also used as reference material for calibrations (Section S2). The optimisation steps were carried out in duplicate.

As anticipated, different flow rates (See SI Section S3.1) did not influence peak shapes, only the retention time. Injection volumes up to 50 µL also showed no alterations in peak shapes, only concentration-dependent increases in peak area (See SI Section S3.2). The injection of concentrations up to 5 mg/mL venom was possible without significant peak shape variation (See SI Section S3.2). To achieve adequate concentrations of venom toxins in the coagulation bioassay after fractionation, a venom concentration of 5 mg/mL and injection volume of 20 µL were selected to pursue the research.

The separation of the venom from *B. multicinctus* showed similar results when different mobile phases were used. Separations of venom from *D. russelii* evaluated with different mobile phase compositions were difficult to compare using SEC-UV data. This was due to the many dissimilarities in retention time profiles (see SI Figure S8). Some peaks showed shifts in retention time with increasing organic solvent concentration in the mobile phase, including a peak eluting after the column volume time. The retention time of this specific peak decreased with increasing concentrations of organic eluent, indicating a reduction in possible secondary interactions with the column material. It is well known that the separation of proteins by SEC can cause interactions with the SEC surface material due to the hydrophobicity of some proteins [25]. A higher concentration of acetonitrile (ACN) in the mobile phase will cause these proteins to be better soluble in the mobile phase and interact less with the SEC material.

Overall, the optimisation of the mobile phase composition showed that 10–20% ACN or isopropanol (IPA), with the addition of 0.05–0.1% acidifier, was sufficient for SEC-based separation (see SI Section S3 Figures S6–S9). However, due to the higher viscosity of IPA in the mobile phase, as opposed to the lower viscosity of the injected samples, the peaks can become unstable and can therefore cause peak splitting [26,27]. Therefore, 10% ACN was chosen as the optimal concentration of the organic solvent in the eluent since it was the lowest concentration at which sufficient separation was achieved and thus had the lowest influence on the possible denaturation of the venom toxins. From the acidifiers added to the mobile phase in concentrations of 0.1% and 0.05%, all had a slightly different influence on the resulting separation. Formic acid (FA) gave the lowest resolution in the chromatographic separations, while trifluoroacetic acid (TFA) and difluoroacetic acid (DFA) both showed improved separation (See SI Sections S3.3 and S3.4). No initial selection was conducted for the tested acidifiers since their presence can also affect ionisation within the MS analysis and the bioactivity of the toxins tested via bioassays. Therefore, the three acidifiers (FA, TFA, and DFA) in both concentrations (0.05% and 0.1%) were incorporated into the downstream experimental approach.

### 2.2. Analytical SEC Implemented to Venom Research

The venoms of the viper *D. russelii* and the elapid *B. multicinctus* were used to evaluate the application of analytical SEC for venom research. Both species are known to have anti- and/or pro-coagulant venom toxins [23,24] but have considerably different mass ranges of toxin constituents present in their venoms, making them ideal models for testing our developed approach. The six mobile phase compositions were tested to gain a better insight into the optimal SEC mobile phase composition. The mobile phase composition needs to be compatible with direct post-column proteomics (i.e., HT venomics), post-column bioassays (i.e., only non-denaturing eluents are allowed), and post-column on-line coupled MS (i.e., only volatile eluents at relatively low pH for efficient positive ESI are allowed). For the SEC separations of the venoms, post-column HT venomics, coagulation bioassay, and high-resolution MS data were thus acquired, allowing an overlay of these datasets to be made. These results will be discussed in the order of SEC-HT-venomics, SEC-bioassays, and SEC-MS in the following sections.

#### 2.2.1. Post-Column HT-Venomics after SEC-Fractionation

High-throughput (HT) venomics was performed on SEC-separated and fractionated snake venoms. The identified toxins are presented in chromatograms plotted by the measured protein score against the retention time of fractionation, in so-called protein score chromatograms (PSCs). The data can be found in SI Section S5 and includes the identified protein names, mascot codes, family, exact mass retrieved from the database, number of post-translational modifications (PTMs) such as glycosylation, and the sum of the protein scores. Additionally, protein score chromatograms (PSCs) for identified toxins for each separation tested condition are given.

HT-venomics data obtained for the six different mobile phase compositions were compared. Since the plates with fractionated venom toxins were vacuum-centrifuged to dryness before performing the HT-venomics step, the mobile phases had no influence on the downstream tryptic digestions and subsequent nanoLC-MS/MS measurements. For *B. multicinctus* venom, a total of 32 Uniprot toxin identities were matched when combining the results for all separately measured 384-well plates containing SEC-fractionated venom. Samples separated using the eluents with the two different TFA concentrations gave the most hits (28 toxins identified), while separations with 0.05% FA in the eluent gave the least hits (22 toxins identified). None of the identified toxins had known coagulation activity according to the sparse functional data associated with the proteins present in the Uniprot database; however, some were described as causing platelet aggregation inhibition which is known to enhance anticoagulation [28].

For *D. russelii* venom, a total of 33 different toxins were detected from all tested SEC separations combined. Most toxins were identified in the fractionated wells from the separation with 0.05% DFA in the eluent (27 venom toxins), and the lowest number of toxins was found for separation with 0.1% TFA in the eluent (19 venom toxins). A toxin from another closely related species, *Daboia palaestinae,* was also retrieved (with a low protein score) in one of the six fractionated plates (separation by 10% ACN + 0.05% DFA). Toxins in the *D. russelii* venom predicted to cause both anti- and pro-coagulant venom effects (according to the Uniprot database) were identified from the HT-venomics data. The anticoagulant toxins detected included two PLA_2_s (PA2B8 and PA2B3) and five snaclecs (SL3, SL4, SL5, SL6, and SL7). Tentative procoagulant toxins found were two snaclecs (SLLC1 and SLLC2), an SVMP (VM3CX), and two SVSPs (VSPA and VSPG).

#### 2.2.2. Post-Column Bioassays after SEC-Fractionation

For *B. multicinctus* venom, aligning the venom SEC-UV separation chromatograms with the bioassay data provided toxin mass ranges where anti- and/or pro-coagulant venom activity occurred, based on the SEC elution time profile. Substantial variations were observed for the coagulation assay results depending on the different separation conditions applied (see SI Section S6 for the detailed results). For example, although anticoagulant venom effects were apparent in five of the six separation conditions tested, this activity was not observed with the eluent condition with 10% ACN + 0.1% TFA. Anticoagulant venom effects have previously been described for *B. multicinctus* venom, with Kunitz-type serine protease inhibitors invoked as the toxin family responsible [29,30]. In a recent case study of a girl bitten by a *B. multicinctus* snake, anticoagulation was clearly visible [31]. For all measurements where anticoagulation was observed, these effects were observed around the same retention time (13–16 min for FA, 16–20 min for TFA and 14–18 min for DFA). In this study, this corresponds to a mass range of approximately 5 to 10 kDa, which includes 3FTxs and Kunitz-type serine protease inhibitors (also classified as the b-chain of ß-bungarotoxins) [32].

Contrastingly, pro-coagulant venom effects were only detected when using the separation conditions containing FA (i.e., 10% ACN + 0.1% FA and 10% ACN + 0.05% FA). Although procoagulant venom effects have been detected in several *Bungarus* species [33,34], to our knowledge, this is the first description of *B. multicinctus* venom. Procoagulation was observed for separations with 0.05% FA at a retention time of 9–13 min and at 9–12 min for separations with 0.1% FA. Within this time range, one peak with a front shoulder was visible which corresponded to a mass range between approximately 10 and 70 kDa (deduced from the retention times and toxin masses of the proteins in the protein standards mix). Because of the large toxin mass range, it was challenging to identify the responsible toxins. However, given that *B. multicinctus* venom is known to have approximately 4% SVMPs [5,35,36] and many SVMPs are procoagulant, it was a logical next step to investigate this further with an SVMP inhibitor. To our knowledge, no coagulation-activating toxins were detected before in the *B. multicinctus* venom. However, a coagulation factor X activation toxin is known to be present in the venom of a closely related species, *B. fasciatus* [34].

**Figure 1:**
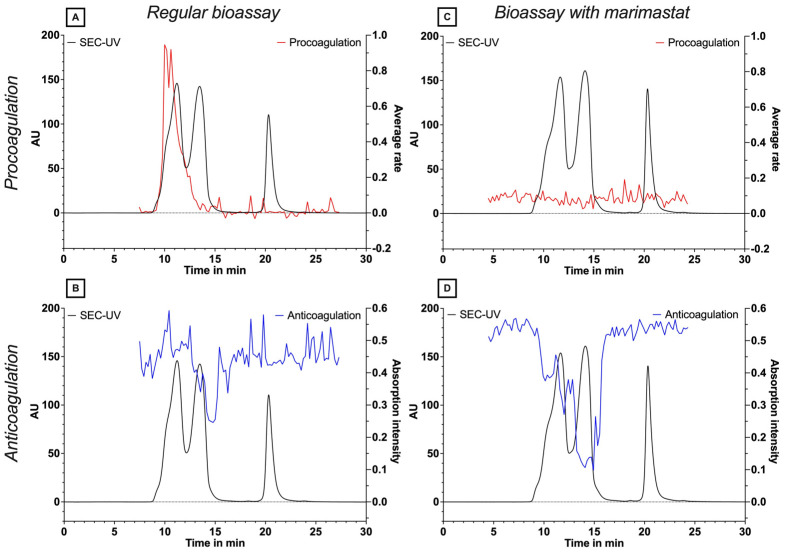
Plasma coagulation assay performed on fractionated plates of SEC separation of *B. multicinctus* venom. The procoagulation activity is depicted by the red positive peak; and the anticoagulation activity by the blue negative peak. The SEC-UV chromatogram is shown in black. Panels (**A**,**B**) show the procoagulant and anticoagulant venom profiles in the absence of marimastat, respectively, while panels (**C**,**D**) show procoagulant and anticoagulant effects in the presence of marimastat.

To investigate whether the observed procoagulant activity of *B. multicinctus* is caused by SVMP toxins, experiments were repeated in the presence of the metalloprotease inhibitor marimastat. *B. multicinctus* venom was separated via SEC using 10% ACN + 0.05% FA as an eluent, and marimastat was added to the coagulation bioassay with a final assay concentration of 4 µM. The results of the bioassay in the presence and absence of marimastat are shown in Figure 1. The procoagulation activity that was first detected with the coagulation assay (Figure 1A) is clearly inhibited by the addition of marimastat (Figure 1C), providing evidence that SVMPs in *B. multicinctus* venom exert procoagulant effects in this bioassay. We also observed a change in anticoagulant venom effects in the presence of marimastat (Figure 1B,D) [37,38], characterised by increased activity. This is possibly the result of coeluting procoagulant SVMP toxins being counteracted by the SVMP inhibitor marimastat, resulting in an unmasking of increased anticoagulant venom potency by other toxins.

For the *D. russelii* venom fractionations, anti- and pro-coagulation peaks were detected with the SEC separations under all six different eluent conditions tested (see SI Section S6 for a detailed overview of these results). *D. russelii* venom is known to contain both SVSPs and SVMPs with procoagulation activity [39]. ‘Slow’ procoagulation was observed mostly at mass ranges where both SVMPs and SVSPs were detected as eluting. For two eluent conditions tested (10% ACN + 0.1% FA and 10% ACN + 0.1% TFA), anticoagulation activity was still observed after the inclusion limit of the SEC column, probably caused by secondary column interactions of the responsible anticoagulant toxins with the SEC column material (See SI document Figure S14). Anticoagulation activity was observed in some cases at retention time areas where no peaks were visible in the SEC-UV and SEC-MS data. From the HT-venomics data, it was shown that anticoagulant PLA_2_s were detected in the same wells as where anticoagulation was detected with the bioassay. The toxins present in *D. russelii* venom that are thought to predominately cause anticoagulant effects are members of the PLA_2_ [40,41,42] or C-type lectin-like (snaclecs) toxin family [43].

**Figure 2:**
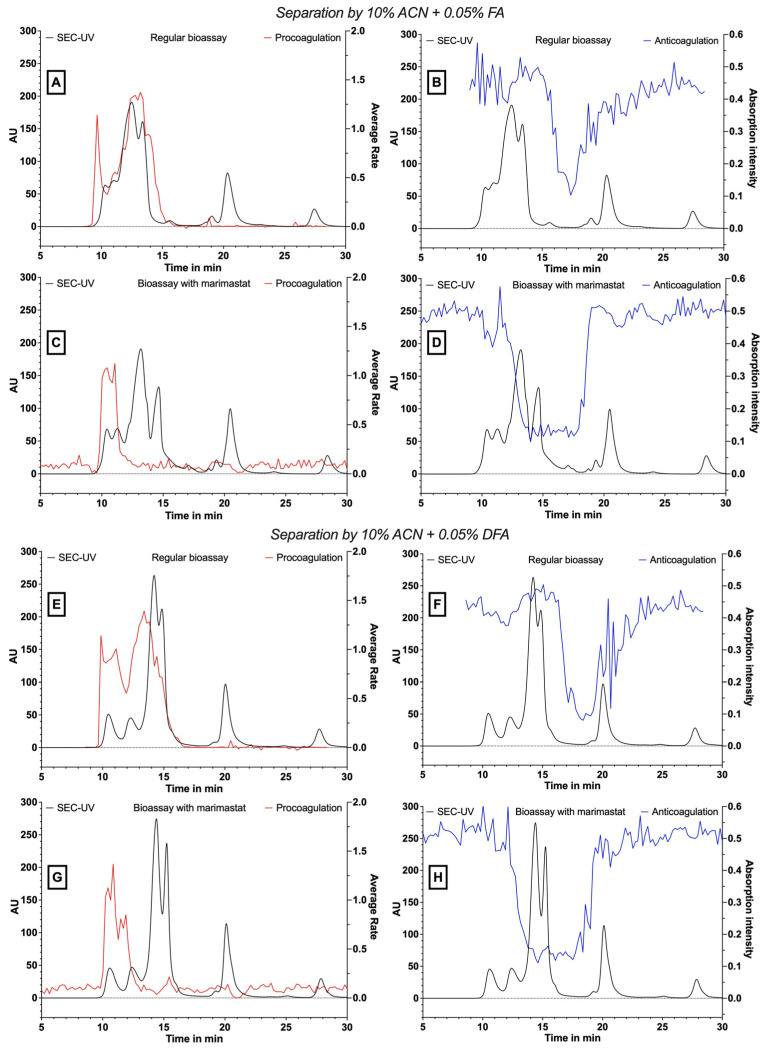
Pro- and anti-coagulation profiles for *D. russelii* venom separated via SEC with various mobile phase conditions. The procoagulation activity is depicted by the red positive peak present in panels on the left and the anticoagulation activity by the blue negative peak present in panels on the right. The SEC-UV chromatogram is shown in black on all panels. The mobile phase conditions used were 10% ACN + 0.05% FA (**A**–**D**) or 10% ACN + 0.05% DFA (**E**–**H**). Panels (**A**,**B**,**E**,**F**) show activity profiles in the absence of marimastat, while panels (**C**,**D**,**G**,**H**) show data in the presence of marimastat.

In line with the experiments described above with *B. multicinctus* venom, next, we repeated our *D. russelii* venom fractionations and performed the bioassay in the presence of the SVMP inhibitor marimastat. Figure 2 shows the results of the coagulation bioassay plotted against the SEC-UV data for both procoagulation and anticoagulation and with and without marimastat for separations performed using 10% ACN + 0.05% FA (Figure 2A–D) and 10% ACN + 0.05% DFA (Figure 2E–H) as eluents. The bioassay results presented in Figure 2 show that the procoagulation bioassay peak profile is substantially reduced, but not completely abrogated, in the presence of marimastat. Concurrent with this, the inhibition of procoagulation is evidence of a larger retention time range containing anticoagulation peak signals, as also observed for *B. multicinctus*. The masking effect of the procoagulant toxins is clearly visible between the non-marimastat- and marimastat-treated chromatographic bioassay results. However, in contrast to the study of Xie et al., 2020, not all procoagulant activity could be neutralised by marimastat [37], which may be due to using an insufficiently high marimastat concentration and/or due to using higher venom concentrations than those used previously. Additionally, the SEC separation used in this study was performed under eluent conditions that are mostly non-denaturing for proteins, whereas the study by Xie et al., 2020 used reversed-phase separations with eluent conditions that might have resulted in the denaturation of some of the SVMPs present in this venom.

#### 2.2.3. Integration and Correlation of Post-SEC Bioassay and HT-Venomics Data

**Figure 3:**
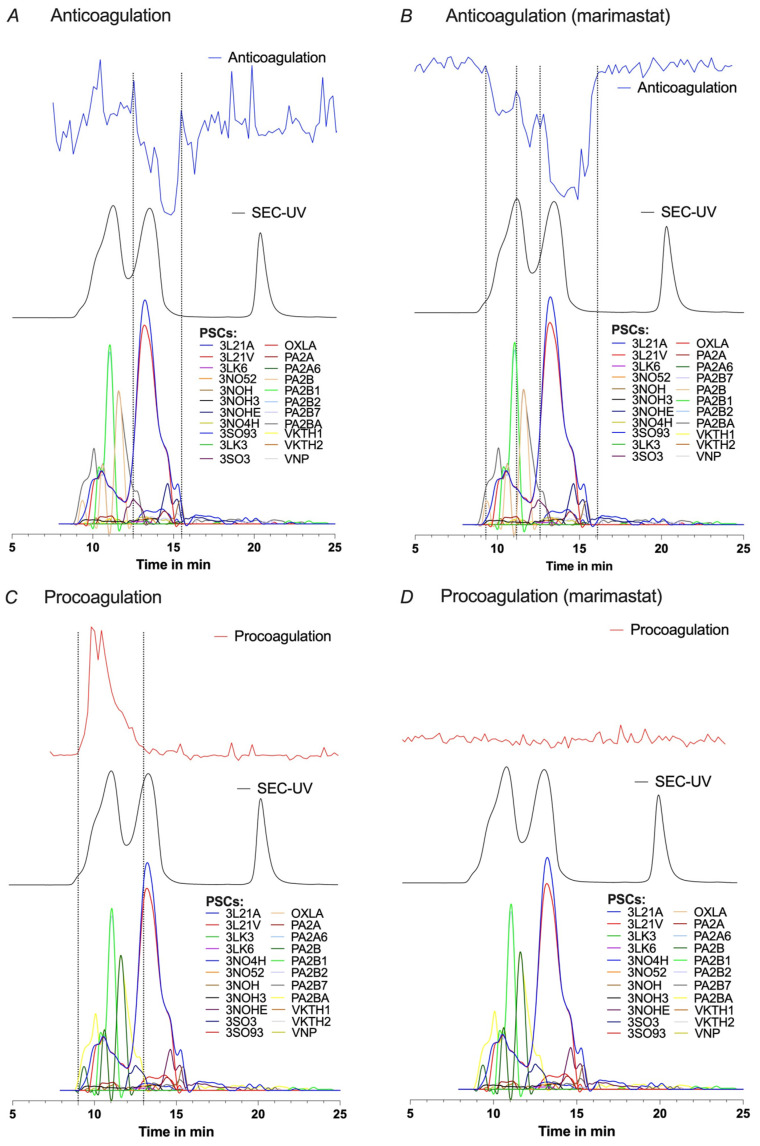
Pro- and anticoagulation observed for SEC-separated *B. multicinctus* venom with 10% ACN and 0.05% FA. Pro- and anticoagulation (upper graph) detected in the *B. multicinctus* venom overlayed with the SEC-UV data (middle graph) and protein score chromatograms (PSCs) (lower graph). The detected anticoagulation (**A**), anticoagulation with marimastat (**B**), procoagulation (**C**), and procoagulation with marimastat (**D**) are overlayed with all PSCs from the HT-venomics data. More detailed information on the identified toxins can be found in SI Section S5 Table S1, and all HT venomics data can also be found in the SI document Prism S4—‘*B. multicinctus* separated by 10% ACN + 0.05% FA’.

**Figure 4:**
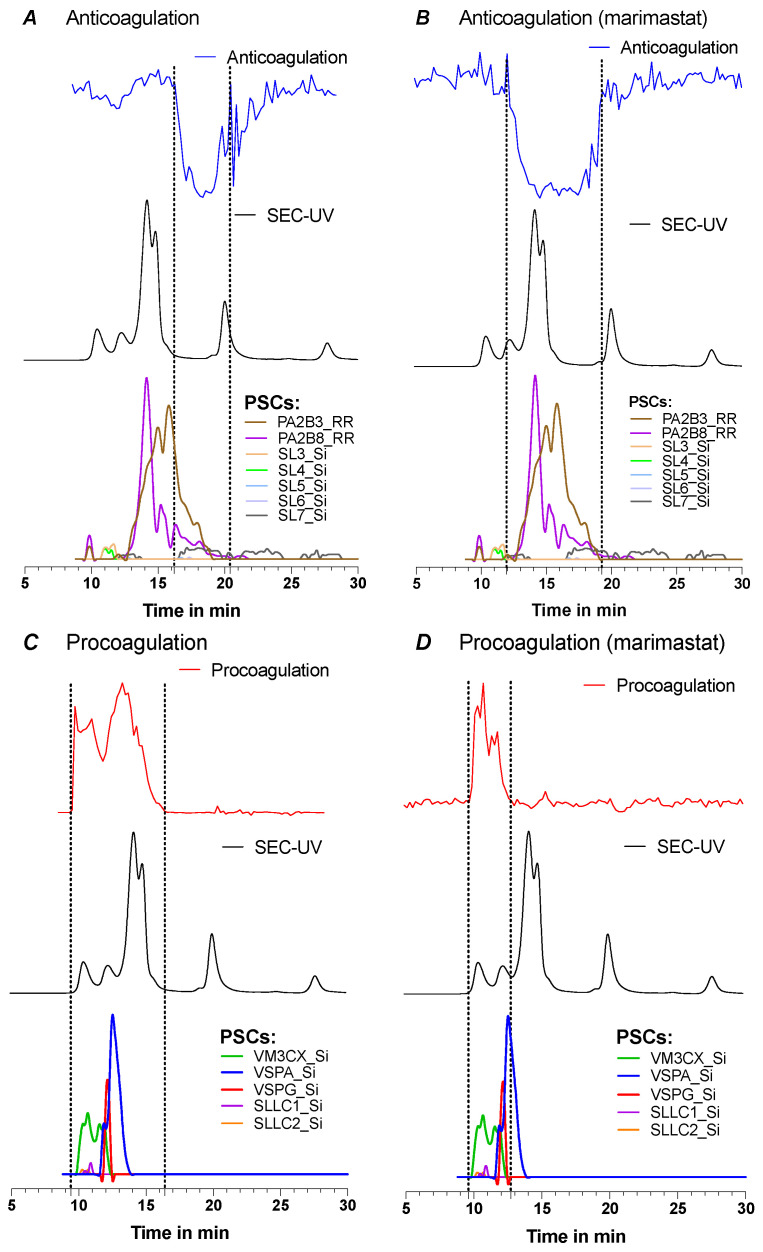
Pro- and anticoagulation observed for SEC-separated *B. multicinctus* venom with 10% ACN and 0.05% FA. Pro- and anticoagulation (upper graph) detected in the *D. russelii* venom overlayed with the SEC-UV data (middle graph) and protein score chromatograms (PSCs) (lower graph) of the coagulant toxins. The detected anticoagulation (**A**) and anticoagulation with marimastat (**B**) are overlayed with the PSCs of the anticoagulant toxins and the detected procoagulation (**C**) and procoagulation with marimastat (**D**) are overlayed with the PSCs of the procoagulant toxins. More detailed information on the identified toxins can be found in SI Section S5 Table S2, and all HT venomics data can also be found in the SI document Prism S10—‘*D. russelii* separated by 10% ACN + 0.05% FA’.

The HT-venomics data from the optimal separation conditions obtained independently for *B. multicinctus* (Figure 3) and *D. russelii* (Figure 4) venoms were overlaid with the corresponding bioassay chromatogram data for each venom. For *B. multicinctus*, separation using 10% ACN + 0.05% FA was chosen. In the presence of the metalloproteinase inhibitor marimastat, most of the identified toxins were present within the retention time frame for the detected anticoagulation (see Figure 3B). For the detected procoagulation activity, mostly PLA_2_s were identified together with several serine proteinases and 3FTxs (all co-eluting in this time frame). The identified toxins in Figure 3 can be divided into groups of 3FTxs or α-/γ-bungarotoxins (codes starting with a 3), a group of PLA_2_s or A-chain of a ß-bungarotoxin (code starts with PA) and Kunitz-type serine proteinases or B-chain of a ß-bungarotoxins (VKTH1 and VKTH2). Since there are many different toxins within the retention times for both the coagulation activities, no exact matches could be made, which was expected for the intrinsically low-resolution SEC separations. In the detected procoagulation elution time frame of 9 to 13 min (the experiment without marimastat; Figure 3C), no toxins with known procoagulation toxicity were identified from Uniprot. From the HT-venomics data, toxins from the families PLA_2_ and Kunitz-type serine proteinase inhibitors were observed within the same area, but these have not yet been reported as procoagulants. On the contrary, they have been identified before as anticoagulant toxins [23,40,44]. In the same elution time range, procoagulation was eliminated when the bioassay including marimastat was used. Since this compound is a metalloproteinase inhibitor, it is highly suggested that an SVMP has caused this activity. Conflicting with this assumption is that no SVMP was detected by the HT-venomics data within this same elution time range. This conflicting result could have different reasons; for example, the toxin causing pro-coagulation could be a toxin which is not in the Uniprot database, the HT-venomics data were not sensitive enough to detect the toxin, or another toxin causing a procoagulant effect was inhibited by marimastat.

Figure 4 shows the corresponding data for *D. russelii* venom, with an overlay provided of the bioassay data (with and without marimastat) and HT-venomics data for the SEC separation, using 10% ACN and 0.05% DFA as the mobile phase. In Figure 4, only the PSCs are plotted that are known for their coagulation-modulating activities identified from Uniprot, which are PLA_2_s (PA2B3 and PA2B8), snaclecs (SL3-SL7, SLLC1 and SLLC2), SVSPs (VSPA and VSPG), and an SVMP (VM3CX). All plotted PSCs are illustrated separately in SI Figure S15. For the bioassay trace without marimastat (Figure 4A), anticoagulation was observed within 16 to 21 min, whereas for the bioassay with marimastat (Figure 4B), anticoagulation occurred within 12 to 19 min. The two anticoagulant PLA_2_s (PA2B3 and PA2B8) identified by HT-venomics fall within this range of 12 to 19 min and therefore match with the anticoagulation profile observed for the bioassay performed with marimastat. The potentially anticoagulant snaclecs were also detected, outside of the main anticoagulation range, and were found at different retention time frames within the whole retention time range. Therefore, it is assumed that these are most likely not the main cause of the anticoagulant venom effects observed. The anticoagulation profile in Figure 4A did not entirely match with the PSC profiles of the anticoagulant toxins (i.e., it was smaller in peak width). Due to the addition of marimastat, the procoagulation activity was inhibited which cleared a path for the anticoagulation to show up. As seen in Figure 4B, the anticoagulation area now detected falls within the same retention time range as the PSCs from the tentative anticoagulant toxins. From Figure 4D, it can be observed that the procoagulation was not completely cancelled out by marimastat, but it did show a procoagulation decrease compared to Figure 4C. The remaining procoagulation trace in Figure 4D matched in terms of retention time and peak shape with the trace of the SVMP found by HT venomics with the Uniprot identifier code VM3CX.

#### 2.2.4. Application and Results of On-Line SEC-MS Analysis

Because we employed watery mobile phases during SEC separation without non-volatile salts as buffer components in the mobile phase (which are usually employed for SEC separations and are not compatible with on-line MS), we could connect the SEC effluent directly to MS to obtain intact toxin MS data for the separated toxins in each venom analysed. With a flow splitter integrated in-between the SEC and the MS, we directed 10% of the flow from the SEC output to the MS, enabling us to accurately measure the high-resolution masses of eluting intact toxins. These SEC-MS data were used as validation for the SEC separations of the toxins in the venoms analysed. The toxin masses found were compared to the exact masses of the toxins retrieved by the Mascot searches from the HT-venomics experiments. All MS data, including the deconvoluted *m*/*z* peaks, the total ion currents (TICs), and the extracted ion chromatograms (EICs), can be found in SI Section S7, Tables S3–S8, Figures S16–S27, and documents PP S1, S2.

##### *Bungarus Multicinctus* 

The separation of *B. multicinctus* venom resulted in three main peaks and one shoulder peak in front of the first peak in the SEC-UV data for every mobile phase composition. Since ionisation sensitivity by ESI decreases upon mass size, the first eluting largest toxins were only observed in very low abundance. Additionally, it is known from this elapid venom (and elapid venoms in general) that larger toxins are only generally present in low abundances [45]. When examining the first main peak observed in the TIC, it was found that it was composed of masses of around 21 kDa, corresponding to the masses of ß-bungarotoxins. ß-bungarotoxins are formed by an A-chain which is a PLA_2_ subunit (±13.5 kDa) and a B-chain that is a Kunitz-type serine protease inhibitor subunit (±7 kDa) both bound together by cysteine bridges according to Uniprot [46,47]. The MS analyses thus indicate that (a fraction of) the ß-bungarotoxins are still intact after separation. The next main peak showed mostly masses of 7–9 kDa, which correspond to 3FTxs and Kunitz-type serine protease inhibitors. Both peaks also showed several masses around 13 kDa which matched with the masses of the PLA_2_s. Since the peaks were not baseline-separated (which is logical for the intrinsically low-resolution SEC separations), it is not unexpected that masses of around 13 kDa are represented in both peaks. The last main peak within the column volume time (also named inclusion limit) only resulted in the detection of around 9 kDa masses when TFA was used in the mobile phase. In this case, secondary interactions with the column are the most likely cause of this. Overall, more toxin masses were detected with a concentration of 0.1% acidifier compared to 0.05% acidifier. The acidifier is necessary for proper ionisation in positive electrospray ionisation mode, which also explains why a higher concentration gives more (intense) peaks. Most toxins were detected with 0.1% DFA, and the least were found for 0.05% FA. Despite FA being the better ioniser according to the literature, in this study, better ionisation performance was clearly obtained in DFA [48].

##### *Daboia Russelii* 

For each acidifier, slight variations in resulting peak distribution were observed. This, for example, resulted in non-baseline separated peaks in the case of separation by 10% ACN + 0.1% FA and 10% ACN + 0.05% FA where two peaks from the SEC-UV chromatogram were fused into one broad TIC peak. Despite these differences, a comparison could be made between eluting masses for all separations tested. No significant masses in the venom toxin range could be derived from the first peak in the TIC for separations with TFA and DFA. The second peak in the TIC for these separations gave 2–5 toxins with masses of around 25 or 29 kDa. The first two peaks of the separations with TFA or DFA were fused into one broad peak in the TIC of separations by FA. From this fused peak for separations by FA, masses of ±25, ±29, ±31, and ±90 kDa were extracted. The highest detected accurate mass was 90,378.34 Da, calculated from an *m*/*z* value of 3477.10 with a 26+ charge. The charge state distribution for this peak was determined manually due to low background noise. To our knowledge, the detection of an around 90 kDa peak for *D. russelii* venom has not yet been shown by ESI-MS but only by MALDI-MS in the past [49]. This mass falls into the mass range of an SVMP known to be present in this venom [16]. The next peak in the TIC was eluted at the same time frame as ribonuclease A from the protein mix. This compound is used as a reference and has a mass of approximately 13.7 kDa. From all separations, masses between 11 and 14 kDa could be deconvoluted from this peak observed in the *D. russelii* analyses. These masses correspond to the mass range of PLA_2_s within this venom. The remaining peaks were expected to contain masses <11 kDa, but this was not the case. There were masses of ±13 and ±27 kDa that were eluted here and even after the column inclusion end time of 21 min. These compounds most likely interacted with the column material and were thus not separated solely based on their size. From the SEC-UV data, a peak was also observed at 28 min retention time (RT), but for this peak, no masses were found in the MS data.

#### 2.2.5. Comparison of All Acquired Post-Column Data Sets

The HT-venomics data were next matched with the parallel obtained SEC-MS data. Matches were made based on the calculated mass, peak shape, and retention time of the PSCs and the EICs. For toxins without post-translational modifications (PTMs), masses derived from the SEC-MS data could directly be matched with the masses of the toxins calculated from their sequence. A PTM such as glycosylation alters the mass, and, therefore, matching the exact deconvoluted masses from SEC-MS to data retrieved from the HT-venomics experiments is challenging. However, it was possible to match several masses to specific toxins (see Table 1), with more matches obtained for *B. multicinctus* than for *D. russelii* venom, likely because toxin types abundant in *B. multicinctus* venom (such as most 3FTx and PLA_2_s) lack PTMs.

The combined analysis revealed several toxins detected in the 20–21 kDa mass range for the *B. multicinctus* venom, which is consistent with the mass range of ß-bungarotoxins, which are composed of a PLA_2_ chain (A-chain) and a Kunitz-type serine proteinase inhibitor chain (B-chain), covalently linked via a cysteine bridge. These two chains were individually identified in the HT-venomics data, where nine A-chains and three B-chains were identified. According to Uniprot, any A-chain can be bound to any other B-chain. Therefore, the exact masses from the SEC-MS data were not matched with one 20–21 kDa toxin from the HT-venomics data but could only be matched with an A-chain of ±15 kDa and a B-chain of ±7 kDa combined. After making calculations, two detected masses from the SEC-MS data could be matched with an A- and B-chain combined, with a difference in mass of ±3 Da (see Table 1). The chains could also be found individually as the reduced chains within the SEC-MS data. An example is the PLA_2_ ß-bungarotoxin A2 chain (Uniprot code PA2B2), of which, the exact mass derived from the Uniprot database matched one mass calculated from the SEC-MS EIC (see Table 1). These data suggest that at least part of these toxin dimers is also present in the venom individually.

**Figure 5:**
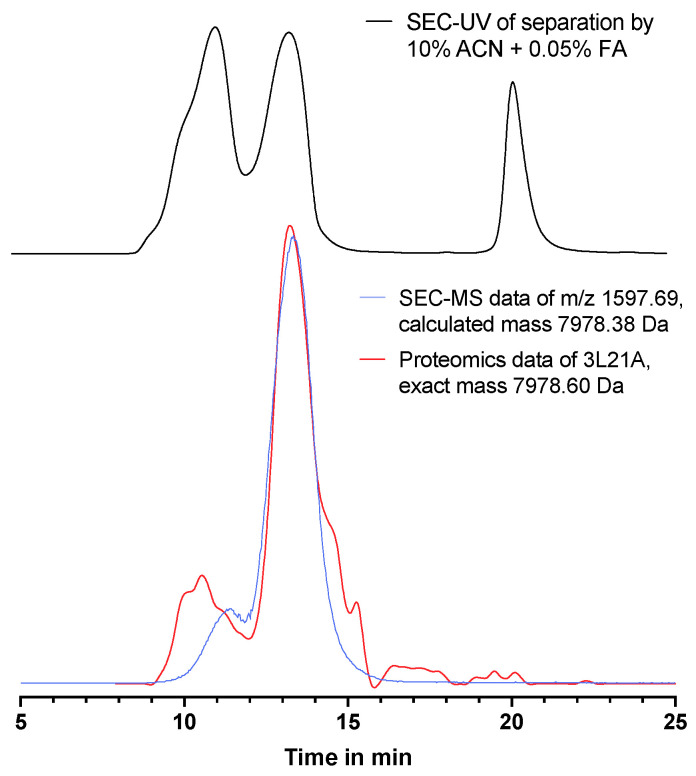
An overlay of the SEC-UV chromatogram (280 nm), HT-venomics protein score chromatogram, and SEC-MS EIC of identified toxin 3L21A present in *B. multicinctus* venom. SEC separation was performed using the mobile phase containing 10% ACN + 0.05% FA. Protein score chromatogram of 3L21A is depicted by the red peak and the EIC of mass 7978.38 by the blue peak. The SEC-UV chromatogram is shown in black.

One of the identified toxins, the toxin with code 3L21A (an α-bungarotoxin), showed an unusual peak shape, appearing as a first small followed by a second larger peak, in both the EIC from the MS data and in the corresponding PSC (see Figure 5). The first small peak in the EIC at 10–12 min corresponded to a mass range above 13 kDa deduced from its retention time in SEC, while the mass range of the larger peak according to its retention time in SEC fits with a mass of around 8 kDa. Since the 3L21A toxin has a mass of ±8 kDa according to Uniprot, it was not expected to elute at the elution time frame of 10–12 min. A hypothesis can be that a certain percentage of this toxin in the crude venom binds to other toxins, causing a larger total mass complex with a consequently earlier retention time for this first small peak. Another explanation could be that this is a dimer of the same toxin. Further research is required to explain this phenomenon.

As for *D. russelii* venom, toxins were found within the mass range of 11 to 14 kDa. This venom contains several PLA_2_s of approximately 13 kDa. No toxins were detected by HT-venomics with around 11 to 12 kDa masses (see SI Section S5), and, therefore, no direct matches could be made for these toxins. The three toxins with a mass between 13 and 14 kDa detected via SEC-MS that showed that a match with toxins from the HT-venomics data (see Table 1) were identified with a relatively high protein score.

**Figure 6:**
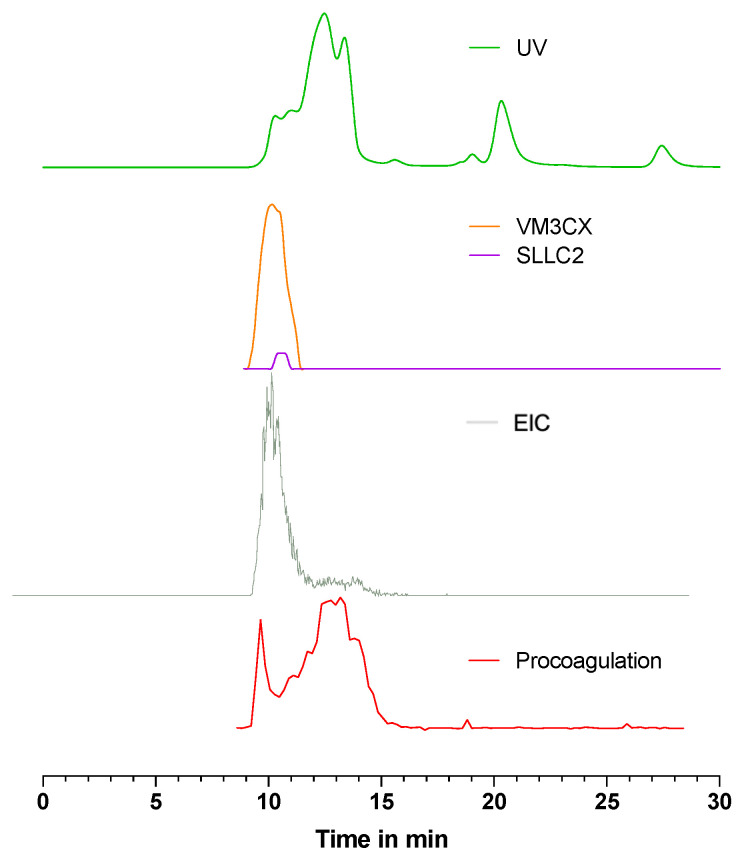
An overlay of analysis results for *D. russelii* venom separated using 10% ACN + 0.05% FA. The SEC-UV chromatogram (green), PSCs of the RVV-X chains VM3CX SVMP (orange), and SLLC2 snaclec (purple), SEC-MS EIC of the 90,378.34 Da compound (*m*/*z* 3477.10, *z* = 26) (grey), and the procoagulation (red) bioassay traces from the bioassay are visualised. For the venomics and bioassay data, measurements were performed after approx. 8 min retention time. The RT range of the procoagulation matches perfectly with the EIC of the 90 kDa component. The dip in the procoagulation, measured by taking the average of the 1st and 15th absorption readings, is caused by an almost instant procoagulation in the first reading, further explained by Figure 7.

*D. russelii* venom contains a coagulation-factor-X-activating glycoprotein (RVV-X) that has a covalently bound heterotrimeric structure [50] and a predicted mass of 78,500.43 Da, based on the protein sequence retrieved by Mascot from the HT-venomics data. This toxin consists of one heavy chain (SVMP VM3CX) and two light chains (snaclecs SLLC1 and SLLC2), where the heavy chain has a total of four oligosaccharides and both light chains each individually have one oligosaccharide, all covalently attached [49,51]. However, the literature suggests this protein is glycosylated, with a native mass of approximately 93 kDa, as analysed by SDS-PAGE, and a mass of approximately 78 kDa after deglycosylation [49]. The individual masses of the chains before and after deglycosylation were 62 to 48 kDa for the heavy chain (VM3CX), 18 to 14 kDa for the first light chain (SLLC1), and 21 to 17 kDa for the second light chain (SLLC2). In this study, the SEC elution time of each of the individual chains identified by HT-venomics fell between the retention times of the bovine serum albumin (BSA) dimer (133 kDa) and BSA (66.5 kDa), measured with the protein mix reference material. This indicates that the three individual chains were likely covalently attached as the toxin trimer RVV-X following SEC separation, further supported by the SEC-MS data which identified a 90,378.34 Da toxin, when FA was used as an acidifier in the mobile phase. This toxin was not detected via SEC-MS when DFA or TFA were used as acidifiers, while the glycoprotein was present in all HT-venomics data (i.e., from the SEC results with FA, DFA, and TFA in the mobile phase), collectively suggesting that only FA provided sufficient ionisation for ESI-MS analyses. To investigate this further, an overlay was conducted for the SEC-UV, HT-venomics, SEC-MS, and bioassay data from a separation of *D. russelii* using 10% ACN + 0.05% FA as the mobile phase (see Figure 6). From Figure 6, the EIC peak shape and retention time plotted from the MS data (3477.10 *m*/*z*; *z* = 26; accurate mass = 90,378.34) matched with the bioassay procoagulation peak. The small shoulder peak in the EIC at time 11.5–15 min explained the extended procoagulation observed. Earlier, in Figure 4, the peak shape of the procoagulation after adding marimastat matched perfectly in retention time and peak shape with that of the PSC of the VM3CX SVMP. After making the correlation between different data in Figure 6, the whole extended procoagulation (measured without marimastat) between 10 and 15 min could also be explained by the same RVV-X toxin.

**Figure 7:**
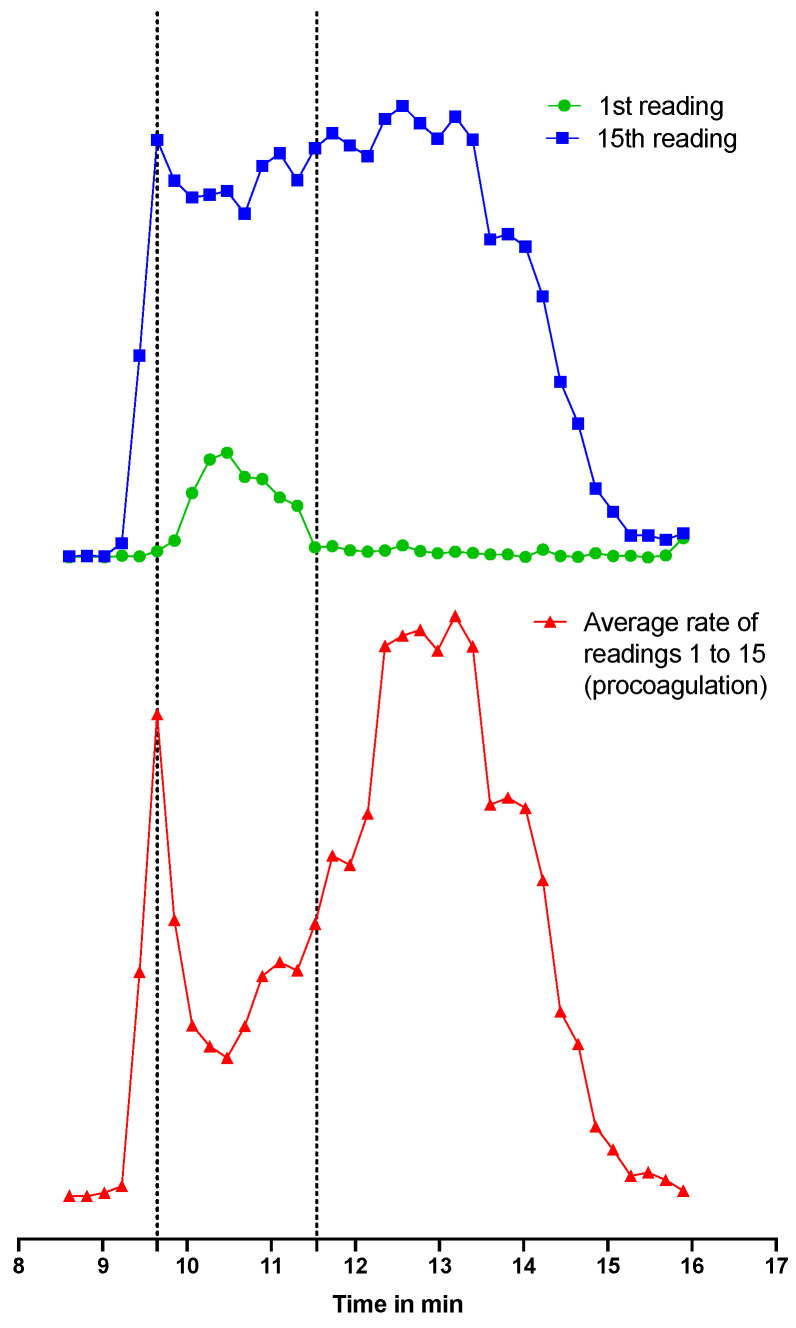
Absorbance spectra of the 1st reading (green), the 15th reading (blue) and the average rate (i.e., slope) of the first 15 readings (red) are overlayed. These readings are from a bioassay measurement of SEC-separated and fractionated *D. russelii* venom. An increase in absorbance was observed between 9.8 and 11.5 min when plotting the 1st reading (implying that procoagulation was already initiated at reading 1), which caused during this time frame a decrease in the average rate from the first 15 readings.

In the same figure, Figure 6, a dropdown of the procoagulation peak for the bioassay chromatograms is observed at approximately 10.5 min. This dropdown, however, does not mean that there is less procoagulation at that point. The same procoagulation is depicted in Figure 7 in red, this time with extra information to explain this dropdown. Every dot in the figure represents a fractionated well from the 384-well plate. The absorbance in each well is measured a total of 80 times (80 readings) with a timeframe of approximately 80 s between each reading. For the procoagulation measurements, an average rate (i.e., slope) was calculated from the first 15 readings of each well, meaning that procoagulation was measured from the starting point until ±20 min. When inspecting the 1st reading at T = 0 (Figure 7 in green) and the 15th reading at T = 20 (Figure 7 in blue) for several wells, it was found that for these wells, the procoagulation was so fast that at T = 0, the procoagulation was already initiated, and as such, the absorbance was already higher than all the other wells (where procoagulation was not yet observed). Thus, almost instant procoagulation activity was observed. Due to this reason, the average rate (i.e., slope) measured for these wells was lower than the ones before and after the observed procoagulation peak dropdown. From the dotted lines in Figure 7, it is clearly observed that plotting the first reading gives a positive peak, which causes a dropdown in the dotted line from the calculated average rate when the 1–15 readings slope is used for the slope calculation.

## 3. Conclusions

The aim of this study was to develop an analytical method to separate snake venom toxins based on their size while retaining their biological activity, using post-column on-line MS analysis. An analytical SEC method using HPLC with low concentrations of organic acidic mobile phases was optimised to perform the separation. A post-column HTS coagulation bioassay provided information about the coagulation-modulating toxins in the snake venoms, where parallel HT-venomics was used to identify these specific toxins. ESI-MS gave accurate masses of eluting toxins and provided information about the capacity of the separation via SEC based on toxin mass. Toxins such as SVMPs and SVSPs tend to lose their bioactivity when separated by the more often used reversed-phase HPLC because of possible alterations in their native structure caused by either column interactions or the high concentrations of organic solvents used in the mobile phase. From the organic solvent modifiers tested, IPA gave high column back-pressures due to its high viscosity and caused peak splitting. ACN (10%) with the addition of an acidifier was found to be the best organic modifier and concentration for the mobile phase. The separations differed slightly from each other, with three acidifiers tested at concentrations of both 0.1% and 0.05%. FA gave the most promising results when analysing the downstream bioassays and MS data, as it was the only condition that enabled the detection of procoagulant activity caused by *B. multicinctus* venom and achieved on-line MS detection of a 90 kDa mass *D. russelii* heterotrimeric toxin with SEC-MS. Contrastingly, TFA and DFA showed better baseline-separated peaks in the resulting SEC-UV data. The ESI-MS results showed that some toxins, predominantly PLA_2_s in *D. russelii* venom, eluted later than most of the other toxins within the same mass range. Therefore, it is important to take into consideration that the SEC column is not always suitable for a perfect size-based separation of all toxins in any given venom. Whether this is a phenomenon that occurs specifically with the SEC column used in this research needs to be tested further by comparison of different analytical SEC columns.

To our knowledge, procoagulant venom activity by *B. multicinctus* venom has not been described before, and the inhibition of this activity by marimastat suggests that it is mediated by SVMP toxins. Toxins in *D. russelii* venom consistently showed both anti- and pro-coagulant activity. HT-venomics was used to gain information on the identity of these coagulopathic toxins, confirming the presence of the heterotrimeric procoagulant toxin RVV-X and demonstrating that it remained intact following SEC separation. Collectively, our data demonstrate that it is feasible to separate snake venom by size using an analytical SEC approach coupled on-line with ESI-MS. Venom HT-venomics and ESI-MS can be performed in parallel and in combination with post-column bioassaying to enable simultaneous functional characterisation and identification of venom toxins that are susceptible to denaturation via more commonly employed reverse-phase separation approaches.

## 4. Methods

### 4.1. Reagents

#### 4.1.1. Chemicals and Solutions

For the SEC optimisation and calibration steps, a protein mix (PM) was made from proteins 1 mg/mL bovine serum albumin (BSA) (mass of 66.5 kDa) and 1 mg/mL ribonuclease A (RNase A) (mass of 13.7 kDa), and nucleotide 0.1 mg/mL uracil (mass of ±100 Da), all purchased from Sigma-Aldrich, dissolved in Milli-Q water, kept cold at 4 °C, and used within one month. Uracil was added as the inclusion limit measurement. The organic solvents used for the optimisation of the mobile phase were acetonitrile (ACN) (Biosolve) and isopropanol (IPA) (Sigma-Aldrich) at eluent concentrations of 5–20% diluted in Milli-Q. Trifluoroacetic acid (TFA), formic acid (FA) (Biosolve), and difluoroacetic acid (DFA) (Sigma-Aldrich) were added to the eluents at final concentrations of 0.05% or 0.1%. All reagents were of analytical grade. For the coagulation assay, 20 mM calcium chloride was prepared by dissolving calcium chloride (Sigma-Aldrich) in Milli-Q water. Bovine plasma used for this assay was purchased from Biowest. The metalloproteinase inhibitor marimastat was purchased from Sigma-Aldrich.

#### 4.1.2. Venoms

Venoms from *Bungarus multicinctus* and *Daboia russelii* were sourced from the historical collection at the division of BioAnalytical Chemistry, Vrije Universiteit Amsterdam (VU). The venoms were stored at −80 °C in their lyophilised form, brought into solution with Milli-Q in Eppendorf tubes at concentrations of 1, 2.5, and 5 mg/mL, aliquoted to the required volumes, and stored again at −80 °C until further use. At the time of analysis, the venoms were taken from the freezer, kept at 4 °C, and used within one week.

### 4.2. SEC-MS Measurements

Size exclusion chromatography was performed using a 4.6 × 300 mm Sepax Zenix SEC-300 column with a 3 µm particle size and 300 Å pore size. The column was operated at 30 °C during the separations using a Shimadzu CTD-30A column oven. The flowrate and injection volumes were varied at first to observe their effects, whereafter the most fit parameters were chosen. Separation was performed using a Shimadzu LC20AB system with a dual pump. The high-performance liquid chromatography (HPLC) system was connected to a Shimadzu SPD 20A UV/Vis detector. Detection was performed at wavelengths of 220, 254, and 280 nm, respectively, where 280 nm was used for HPLC-UV data analysis. HPLC-UV data was acquired by and processed with Shimadzu LabSolutions software and was exported to PRISM software for plotting the HPLC-UV chromatograms. For electrospray ionization mass spectrometry (ESI-MS) analysis, a Bruker Maxis II quadrupole time-of-flight (Q-TOF) mass spectrometer was used. SEC-MS was performed online where a fraction of 10% from the HPLC effluent was sent to MS using a post-column flow splitter. An electrospray ionisation (ESI) source was applied in positive mode and ions were scanned in a mass range of 800–5000 *m*/*z*. Other parameters were 500 V for the end plate offset, 4500 V for the capillary, 1.5 bar for the nebuliser, 8 L/min dry gas, and 200 °C as the dry temperature. MS calibration was performed using an ESI-L low-concentration tune mix (Agilent). From the on-line SEC-MS data, the total ion chromatograms (TICs) and extracted ion chromatograms (EICs) for each venom separated with every different mobile phase solution used were plotted and depicted in two PowerPoint documents. The TICs and EICs for *B. multicinctus* are visualised in ‘PP S1 SEC-MS *B. multicinctus*’, and for *D. russelii* in ‘PP S2 SEC-MS *D. russelii*’.

### 4.3. Nanofractionation

High-resolution fractionation was performed during the HPLC runs, after the flow split, using 90% of the effluent portion for fractionation into transparent flat-bottom polystyrene 384-well plates (781185, Greiner Bio One), making use of a FractioMate^TM^ FRM100 nanofraction collector (SPARK-Holland and Vrije Universiteit Amsterdam). A fractionation resolution of 12.5 s was used, resulting in approximately 37.5 µL eluent collected per well at a flow rate of 0.2 mL/min. Within 1 h after fractionation, plates containing volatile eluent were vacuum-centrifuged overnight using a Christ Rotational Vacuum Concentrator RVC 2-33 CD plus (Salm and Kipp) and kept at −20 °C until further use. The delay time between the UV/Vis-detector and the fractionation system was measured by injecting 10 µL of 0.1 mg/mL argatroban, fractionating this anticoagulant compound after separation, and then performing the plasma coagulation assay on the plate containing the fractionated argatroban. The resulting negative peak in the bioassay chromatogram was subsequently compared to the argatroban peak observed in the UV/Vis chromatogram from which a delay of 0.6 min was observed.

### 4.4. HT-Venomics

To identify the SEC-separated toxins, venom proteomics (i.e., HT-venomics) was performed using the methodology of Slagboom et al., 2023 [21]. This was carried out for SEC-separations of both snake venoms included in this study, each investigated under six different mobile phase compositions, each containing 10% ACN and either 0.05% or 0.01% FA, TFA, or DFA. For HT-venomics, in-solution tryptic digestions of the SEC-nanofractionated toxins in vacuum-centrifuged well plates were conducted. Using robotic pipetting, 25 µL of reduction buffer consisting of 25 mM ammonium bicarbonate and 0.05% 2-mercaptoethanol was added to each well, whereafter the plates were incubated for 15 min at 95 °C. The cysteines from the reduced disulphide bridges were then alkylated by adding 10 µL alkylation buffer (12.5 mM iodoacetamide) followed by incubation at room temperature for 30 min in the dark. Next, 3 µL of a solution containing 0.1 µg trypsin was added and the plates were incubated overnight at 37 °C. The next day, quenching of the digestion was performed by adding 10 µL of 1.25% formic acid. After this last step, the plates were stored at −20 °C prior to analysis.

The analytical part of the HT-venomics procedure was accomplished by nanoLC-MS/MS. The digested samples were separated by an UltiMate 3000 RSLCnano system (Thermo Fisher Scientific, Breda, Netherlands). This system used an autosampler which allowed direct sampling from the 384-well plates. A volume of 1 µL was injected in a C18 nanoLC column (150 mm × 75 µm) with a 2 µm particle size and 100 Å pore size, coupled with a trapping C18 column (5 mm × 0.3 mm) with a 5 µm particle size and 100 Å pore size. Both columns were from Acclaim™ PepMap™ 100 (Thermo Fischer Scientific). The column oven was set to 45 °C. The mobile phase included eluent A (2% ACN and 0.1% FA in Milli-Q) and eluent B (98% ACN and 0.1% FA in Milli-Q), both used in a gradient program. This gradient was: 3 min isocratic separation at 1% B, linear increase to 40% B in 7.5 min, linear increase to 85% B in 0.1 min, isocratic separation at 85% for 0.7 min, linear decrease to 1% B in 0.2 min and isocratic separation at 1% B for 3.7 min. A flow rate of 5 µL/min was used. The nanoLC was connected to a MaXis II Q-TOF MS operated in positive-ion mode. The nanoESI source had the following parameters: capillary voltage, 1.6 kV; source temperature, 150 °C; gas flow, 3.0 L/min; and nanoBooster pressure, 0.20 bar. The mass range was set from 50 to 3000 *m*/*z*. MS/MS was performed via collision-induced dissociation (CID) in data-dependent mode using 10 eV collision energy. Data were collected and processed by Bruker DataAnalysis software. Finally, the identification of the toxins was performed using MASCOT (Matrix Science) searches against Swiss-Prot and species-specific databases using the same search conditions as Slagboom et al. in 2023 [21]. The proteomics data were plotted in PRISM; see SI PRISM S3-S8 for *B. multicinctus* and PRISM S9-S14 for *D. russelii*.

### 4.5. Plasma Coagulation Assay

An HTS (high-throughput screening) coagulation assay described by Still et al., 2017 was utilised in this study to measure the effect on plasma coagulation caused by different toxins in the fractionated samples of the analysed venoms [22,37]. Bovine plasma incubated with Ca^2+^ results in a kinetic coagulation profile measured by spectrophotometric instrumentation, as upon coagulation, the plasma becomes less transparent, hence resulting in higher absorbance. The coagulation bioactivity of crude venoms and venom toxins can be measured by testing the influence on the coagulation profile kinetically (i.e., by measuring the coagulation curve in time). Whether a compound is activating or inhibiting the coagulation process depends on the velocity of the plasma clotting. A high velocity, meaning a fast increase in absorbance and steep slope, is caused by a procoagulant toxin, while an anticoagulant toxin is responsible for a slow increase in absorbance.

To reduce the potential for different toxins coeluting after separation simultaneously causing competing bioactivities (i.e., pro- and anti-coagulation), we also used the small-molecule-inhibiting drug marimastat (i.e., a metalloprotease inhibitor) to investigate the effect of inhibiting procoagulant SVMPs after chromatographic separation. The concentrations of marimastat used were selected based on Xie et al., 2020 [37]. Inhibitors for SVSPs were not included since they tend to inhibit plasma clotting factors as well, and additionally would influence the plasma coagulation assay. See the supporting information (SI) Section S1 for the complete coagulation bioassay procedure. Collected data for anti- and procoagulation were plotted in PRISM; see SI PRISM S3-S8 for *B. multicinctus* and PRISM S9-S14 for *D. russelii*.

## Figures and Tables

**Table 1:** Toxins that were matched from the HT-venomics data with the MS data (mass difference of max 5 Da). SEC-MS derived masses are derived from separations using 10% ACN + 0.05% FA.

Mascot Code	Name	HT-Venomics Retrieved Mass	MS Accurate Mass
*Bungarus multicinctus*			
*3L21A_BUNMU*	α-Bungarotoxin	7978.60	7978.38
*3L21V_BUNMU*	α-Bungarotoxin Isoform V31	8003.63	8005.39
*3LKB_BUNMU*	κ-bungarotoxin	7260.22	7259.92
*3NO5I_BUNMU*	γ-bungarotoxin	7519.33	7517.11
*3NOH_BUNMU*	Toxin BMLCL	9012.78	9012.52
*3NOHE_BUNMU*	Muscarinic toxin BM14	9068.89	9066.61
*PA2A_BUNMU*	Acidic PLA_2_	12,808.56	12,808.20
*PA2B2_BUNMU*	Basic PLA_2_ ß-bungarotoxin A2 chain	13,656.20	13,652.88
*PA2B_BUNMU* + *VKTH3_BUNMU*	Basic PLA2 ß-bungarotoxin A-AL2 chain + Kunitz-type serine protease inhibitor homolog beta-bungarotoxin B3 chain	13,575.11 + 7205.28 = total 20,780.39	20,776.95
*PA2B7_BUNMU + VKTH2_BUNMU*	Basic PLA2 ß-bungarotoxin A7 chain + Kunitz-type serine protease inhibitor homolog beta-bungarotoxin B2 chain	13,445.08 + 7186.37 = total 20,631.45	20,634.90
*Daboia russelii*			
*PA2B_DABRR*	Basic PLA_2_ RVV-VD	13,602.94	13,603.94
*PA2B5_DABRR*	Basic PLA_2_ VRV-PL-V	13,563.13	13,568.91
*PA2B8_DABRR*	Basic PLA_2_ VRV-PL-VIIIa	13,587.20	13,585.95

## Data Availability

Not applicable.

## Supplementary Materials

The following supporting information can be downloaded at: https://www.mdpi.com/article/10.3390/toxins15090552/s1. References [52,53,54,55,56,57] are cited in Supplementary Materials.
