# Supplementary material for: Analytical Size Exclusion Chromatography Coupled with Mass Spectrometry in Parallel with High-Throughput Venomics and Bioassaying for Venom Profiling
Source: Toxins (Basel). 2023 Sep 5;15(9):552. doi: 10.3390/toxins15090552 (PMC10534738; doi:10.3390/toxins15090552)
Supplement: Supplementary file 1 [file toxins-15-00552-s001.zip › toxins-2526775 SM v2.0.pdf]

# Supplementary Information

# Analytical Size Exclusion Chromatography Coupled with Mass Spectrometry in Parallel with High-Throughput Venomics and Bioassaying for Venom Profiling

## Table of content


S1. Plasma Coagulation Assay Protocol ........ 2

S2. Protein Mix separation ........ 2

S3. Optimisation of the SEC measurements ........ 3

S3.1. FLOW RATES ........ 3
S3.2. INJECTION VOLUME & VENOM CONCENTRATION ........ 4
S3.3. MOBILE PHASE COMPOSITION ........ 5
S3.4. SEC OPTIMISATION WITH VENOM ........ 10

S4. Venom composition ........ 14

S5. Proteomics Results ........ 15

S6. Plasma coagulation assay results ........ 19

S7. SEC-MS raw data ........ 22

S7.1. DECONVOLUTION DATA ........ 22
S7.2. TICS AND EICS ........ 29

S8. Reference material ........ 42

# S1. Plasma Coagulation Assay Protocol

A HTS (high-throughput screening) coagulation assay was utilised in this research to measure the effect on plasma coagulation caused by snake venom toxins [1]. Bovine plasma incubated with $Ca^{2+}$ results in a kinetic coagulation profile measured by spectrophotometric instrumentation as upon coagulation the plasma becomes less transparent hence resulting in higher absorbance. Coagulation bioactivity of crude venoms and venom toxins can be measured by testing the influence on the coagulation profile kinetically (i.e. by measuring the coagulation curve in-time).

The assay was performed at room temperature on transparent flat-bottom polystyrene 384-well plates (781185, Greiner Bio One, Alphen aan den Rijn, The Netherlands) filled with pre-nanofractionated and freeze-dried venom separated by SEC. First, 20 µl of 20 mM $CaCl_2$ was added to the wells, whereafter the plate was incubated for 10 minutes on a shaker at low shaking velocity. In the meantime, bovine plasma at room temperature was centrifuged for 4 minutes at 2000 rpm to get rid of particulate matter (e.g., some remaining white blood cells). After 10 minutes of incubation, 20 µl of the citrated plasma was added to the wells. The plate was then transferred to the plate reader and the measurement program was started within 1 minute. Pipetting calcium chloride and plasma was performed by a robotic pipetting system, ThermoScientific™ Multidrop™ 384.

The platereader, a Thermo Fisher Scientific Laboratory Varioskan™ LUX Multimode Microplate Reader with data analysis program SkanIt 4.1., was set to absorption at 595 nm. All 384 wells were read 80 times, resulting in a total reading time of approximately 100 minutes. Whether a compound is activating or inhibiting the coagulation process, depends on the velocity of the plasma clotting. A high velocity, meaning a fast increase in absorbance and steep slope, is caused by a procoagulant while an anticoagulant is responsible for a slow increase in absorbance. To display anticoagulation, the absorption of the 80$^{th}$ reading was taken and plotted. At this point the non-anticoagulant wells have reached the full coagulation endpoint while anticoagulation reached a lower absorbance endpoint measurement. For assessment of procoagulation, the steepness of the coagulation slope was used. For this, the average velocity of the first 15 measurements were used. If there is activation of coagulation in the first 15 readings, the sample will be labelled as procoagulant. Approximately 80 seconds pass between each measurement, meaning procoagulation is measured within 20 minutes.

Coagulation is not specifically caused by one toxin family. Russell’s viper venom for instance contains a metalloproteinase causing procoagulation and $PLA_2$s causing anticoagulation. When both inhibition and activation of coagulation is present in the same well, the procoagulant compound can cancel out the anticoagulation [2]. For this reason the pharmaceutical compound marimastat which inhibits the potential procoagulant activity of metalloproteinases was used in an additional assay on SEC-fractionated venom. A total of 10 µl of marimastat was added to the fractionated and vacuum centrifuged wells, followed by centrifuging the well plates for 1 minute at 1000 rpm before incubation of 30 minutes at room temperature. Afterwards, the same steps were performed as for the assay without marimastat. The concentration of marimastat was chosen according to the research of Xie *et al* 2020, which concluded in an optimal end concentration in the wells of 4 µM [2].

# S2. Protein Mix separation

A protein mix made from 1 mg/ml BSA, 1 mg/ml Ribonuclease A and 0.1 mg/ml uracil was used to test the separation efficiency and capacity of the SEC column during the method optimisation steps. Figure S1 shows a chromatogram of the SEC separation of the protein mix using a mobile phase of 20% ACN + 0.1% TFA at a flow rate of 0.2 ml/min with an injection volume of 20 µl. UV detection was done at a wavelength of 280 nm. Separation of the compounds in the mixture including the BSA dimer formed in solution was achieved. Identification of the individual peaks was verified by measuring all compounds individually.

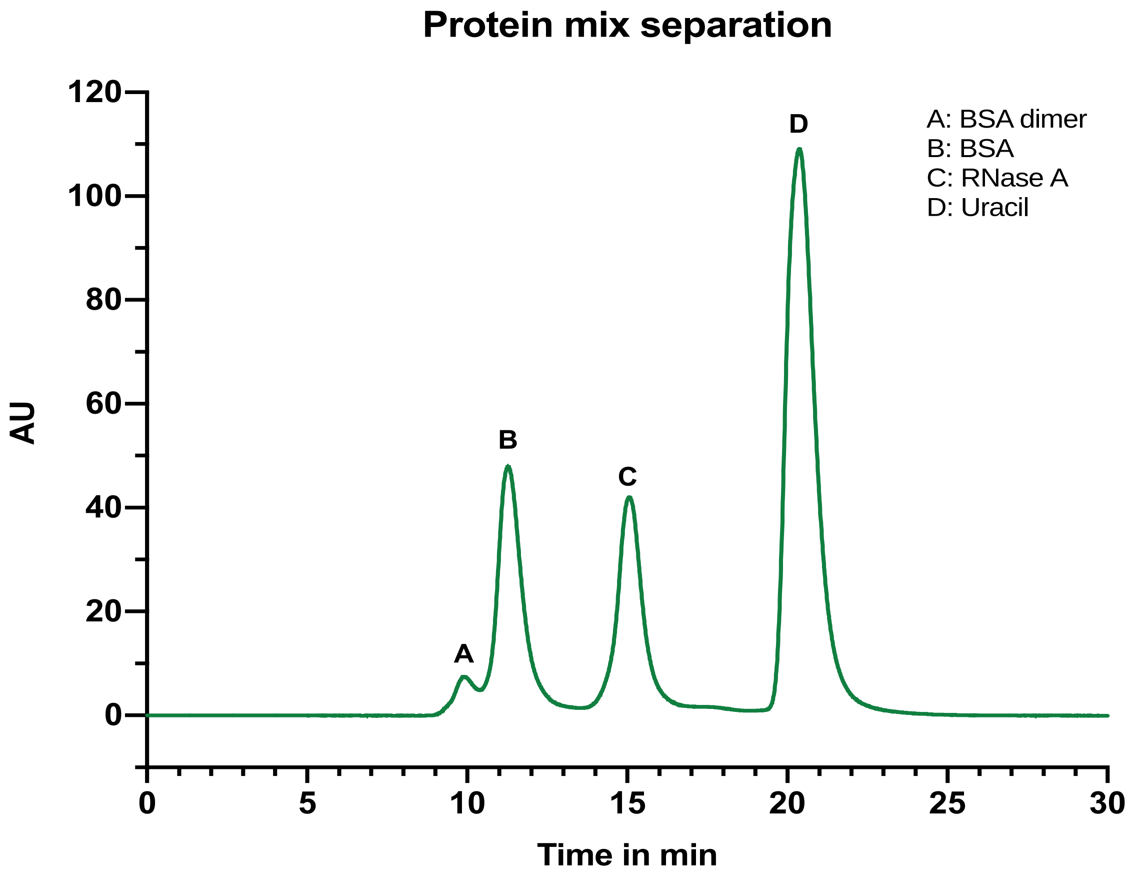


*Figure S1: SEC separation of the protein mix using 20% ACN + 0.1% TFA as a mobile phase. 20 µl was of a 1 mg/ml bovine serum albumin (BSA), 1 mg/ml ribonuclease A (RNase A) and 0.1 mg/ml uracil mixture was injected.*

# S3. Optimisation of the SEC measurements

There are several relevant parameters that influence a SEC separation, of which many are column specifications. The column length plays a role in the resolution of the peaks and back pressure. The particle diameter and their pore size highly influence the retention range and the selectivity of the column. Other influencing non-column specific parameters are the temperature and mobile phase. The flow rate does not show an effect on the selectivity of the separation but is correlated with retention times which is important for throughput. The mobile phase composition is of influence for potential secondary column material interactions of analytes, which should be avoided at all times. Additionally, the amount of sample injected into the column must be chosen carefully to avoid overloading the column which can lead to non-repeatable results. This is specifically important to keep in mind when using highly concentrated and protein-rich snake venom samples. A selection of parameters for optimisation was made including the composition of the mobile phase, injection volume and flow rate. All optimisation steps were measured in duplicate, for which no significant differences were observed in any optimisation step.

## S3.1. Flow rates

Three different flow rates, 0.1, 0.2 and 0.3 ml/min, were tested by injecting the protein mix or *B. multicinctus* venom. An injection volume of 10 µl and mobile phase composition of 20% ACN + 0.1% TFA were used. The results showed no alterations in peak shapes nor resolution. The only difference is the retention time frame in which the peaks eluted, as expected. See Figure S2 for the results.

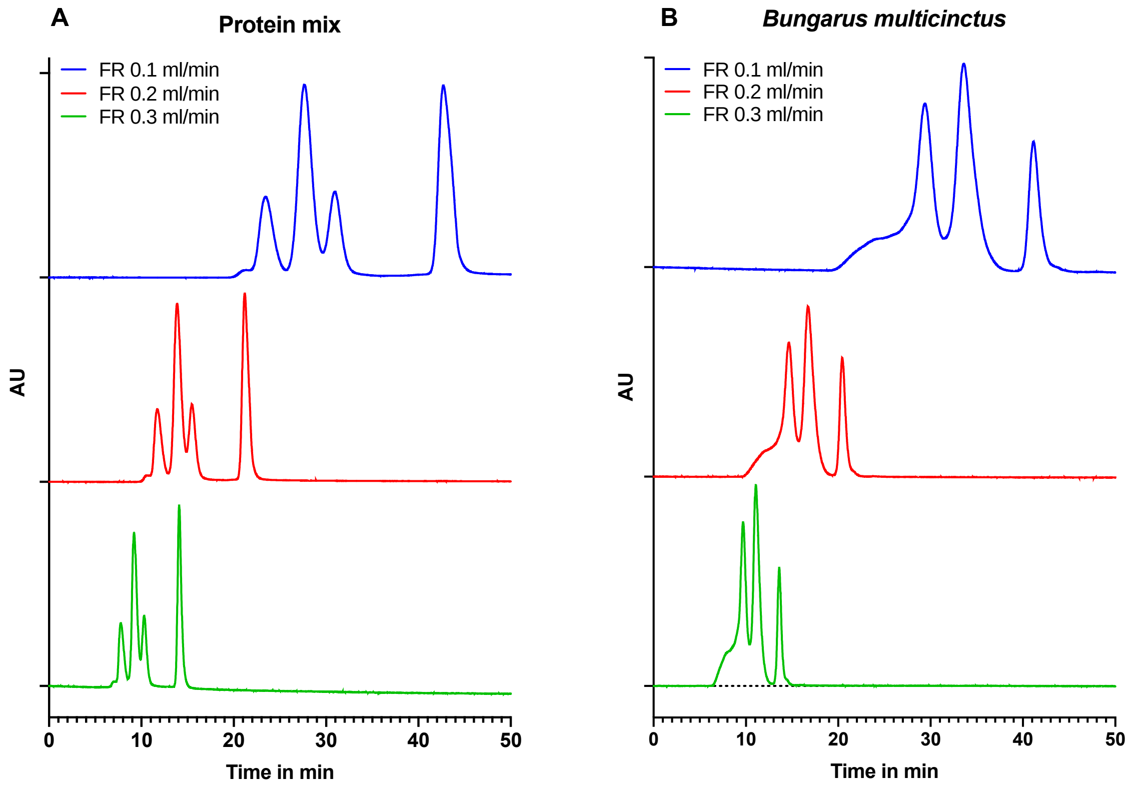


*Figure S2: A; Flow rate (FR) evaluation for injection of the protein mix. B; Injection of 1 mg/ml B. multicinctus venom.*

## S3.2. Injection volume & venom concentration

Optimal injection volumes for the SEC column used as advised by the manufacturer are between 3 and 20 µl. In this study however, higher injection volumes were potentially desired due to the post-column bioassaying after fractionation. Higher injection volumes allow more sample to be injected and thus resulting in higher concentrations of fractionated toxins in the eventual bioassay. A lower sample concentration and/or a lower injection volume can result in less to no activity [3]. In previous studies with post-column bioassaying, injection volumes up to 50 µl were used, for separation by RPLC. Here, injecting venom concentrations of 1 to 5 mg/ml was normally done [1,4,5]. In this study, evaluation of injection volume was performed by injecting volumes of 5, 10, 20, 30, 40 and 50 µl of 1 mg/ml venom and by 20 µl injections of venom at concentrations of 1, 2.5 and 5 mg/ml.

Venom of *B. multicinctus* was chosen for this evaluation. As mobile phase, 20% ACN + 0.1% TFA was chosen. Separations were performed at a flow rate of 0.3 ml/min resulting in a total void time (the column volume) of 15 minutes. Variation in venom concentration as well as injection volume did not provide deviations in the separation efficiency (see Figure S3). The retention times of the peaks were the same for all separations where only the intensity of the peaks increased with increasing concentration and/or injection volume. There was no case of overloading the column or dissimilarities in peak retention times.

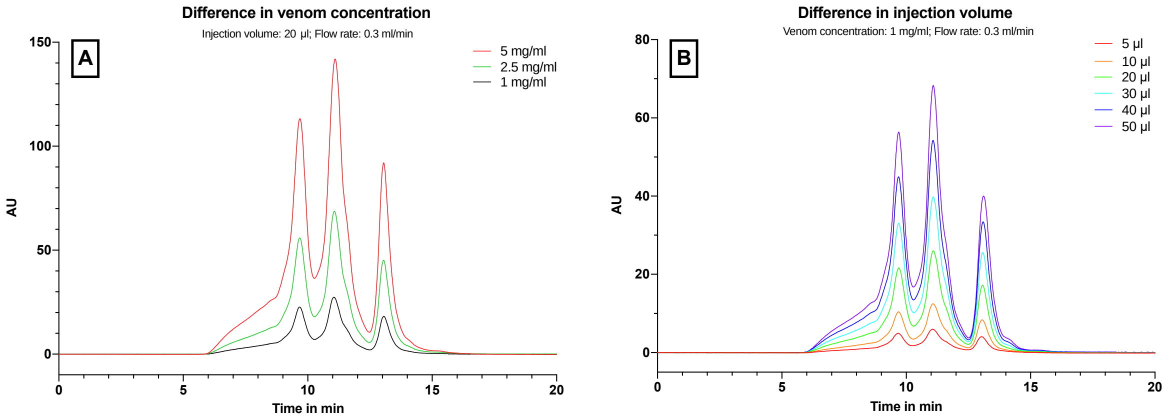


*Figure S3: SEC separations of 20 µl injections of B. multicinctus venom at concentrations of 1, 2.5 and 5 mg/ml (A) and SEC separations of 1 mg/ml B. multicinctus venom with injection volumes of 5, 10, 20, 30, 40 and 50 µl (B). Separations were performed at a flow rate of 0.3 ml/min. A lower concentration venom and a lower injection volume both influenced only the intensity of the peaks. There was no overloading of the column and no differences in the separation observed from this data.*

## S3.3. Mobile phase composition

Different mobile phase compositions were tested by separating initially the protein mix and afterwards the venoms (see S3.4 for the venom separations). All raw data are plotted in Prism documents, see SI documents Prism S1 for the protein mix separations and Prism S2 for the venom separations.

### *Effect of Organic solvent concentration*

Organic solvents acetonitrile (ACN) and isopropanol (IPA) were first tested at five concentrations: 0%, 5%, 10%, 15% and 20%, without the addition of acidifier. In all cases, no separation of the proteins within the protein mix was observed, see Figure S4. There was one peak visible which eluted at the column volume time of t=21 min. Concentrations of 5-20% ACN showed almost gaussian peaks, but when compared to separation by 100% Milli-Q, the peak showed a bit tailing. Fronting with irregular peak shape was observed for separation with IPA in the eluent. With increasing concentration of IPA, the peak became broader and showed a bump shape at the front of the peak. Under none of the investigated conditions, the compounds in the mixture were separated from each other.

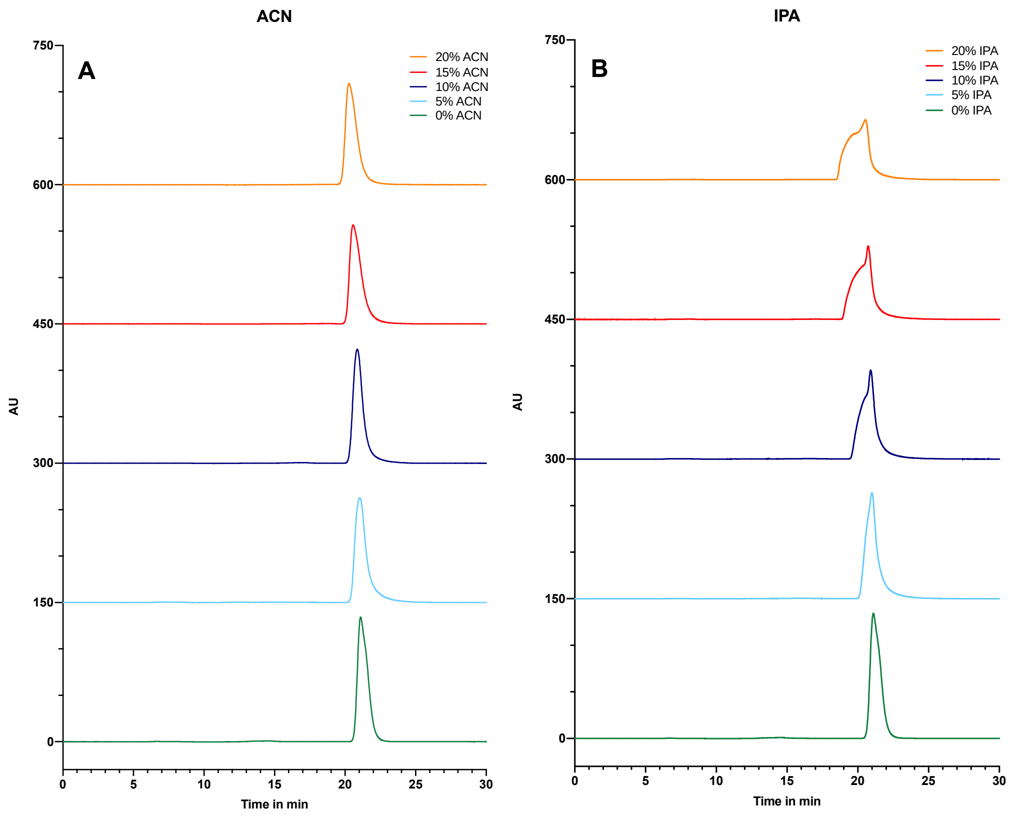


*Figure S4: Separation of the protein mix under 5 different concentrations of ACN in the eluent (**A**) or 5 different concentrations of IPA in the eluent (**B**). Under none of the investigated conditions, the compounds in the mixture were separated from each other.*

### *Effect of the Acidifiers*

The acidifiers included in this study were first tested individually by adding a percentage of 0%, 0.05% or 0.1% to Milli-Q and using this eluent separating the protein mix, see Figure S5. Addition of TFA gave separation of two different peaks in case of both concentrations of 0.1% and 0.05% tested with a slight difference in elution time of the first peak. The difference in retention time between peak 1 and 2 was larger when a concentration of 0.05% TFA was used.

In contrary to TFA, separation with FA as acidifier gave three peaks, which was the expected situation since there are three different proteins in the protein mix. Between the retention time frame of 9 to 13 minutes, two peaks were visible which is contradicted to the separation by TFA. Addition of an acidifier to the mobile phase for the SEC separation gave improvement in the chromatographic performance. TFA in this regard is called the golden standard when dealing with RPLC separations since it positively increases the resolution [6]. A downside of TFA is that it suppresses the ion signal in MS, causing poor MS data due to ion suppression. TFA, with a pKa value of 0.3, is also a very strong acidifier and this might affect the proteins native form. DFA was proven to be a good alternative for TFA in RPLC separations because of the small loss in resolution as compared to TFA used as acidifier [6]. DFA is a less strong acidifier, but nevertheless, still categorised as strong with a pKa of 1.34. Comparing both acidifiers as additions to the mobile phase v for SEC separations, DFA gave a better separation than TFA. The reason for this could be that TFA is too harsh on the proteins, causing alterations in their form. Studies have shown that a lower pH can cause denaturation of proteins [7]. This could also be an explanation for the better separation with FA compared to TFA.

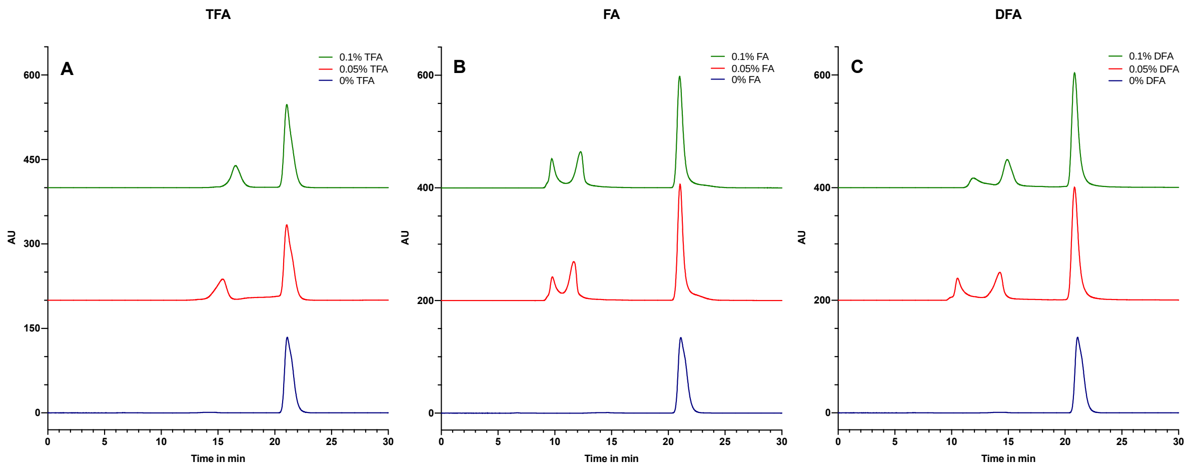


*Figure S5: Separation of the protein mix tested with three different concentrations of the acidifiers TFA (A), FA (B) and DFA (C)*

### *Effect of combinations of both organic solvent and acidifier together in the eluent*

First, protein mix separation was investigated by four different concentrations of ACN in combination with 2 different concentrations of acidifier TFA, FA or DFA (see Figure S6) In most cases, the separation performance increased with increasing concentration of ACN. Between acidifiers DFA and TFA small differences were observed in peak shape and retention times. DFA gave slightly better resolution compared to TFA, and also in combination with 5% ACN was still capable of separating the (first) two eluting proteins. FA gave poorer separation when looking at the first two (protein) peaks. Also, the BSA-dimer peak was not observed for any separation with FA.

The minimum concentration of ACN resulting in a good separation depended highly on the acidifier and its concentration added. For instance, when looking at the separation by ACN + 0.05% or 0.1% TFA in Figure S6 it is seen that all proteins in the protein mix were separated with a minimum concentration of 15% ACN + 0.1% TFA and a minimum of 10% ACN + 0.05% TFA. The BSA-dimer was best separated with 0.1% TFA + 15% or 20%, and by 0.1% DFA with all concentrations ACN tested.

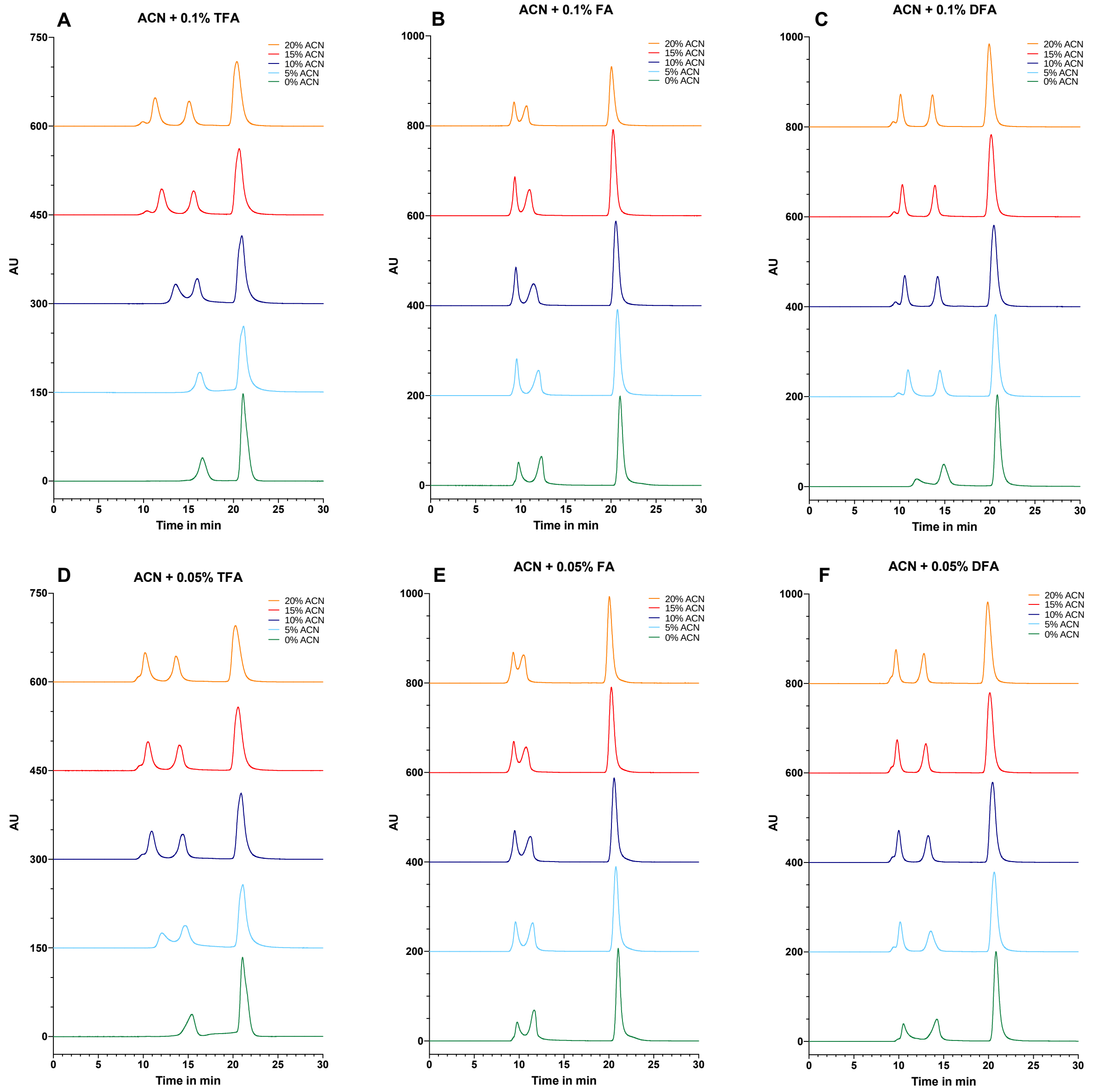


*Figure S6: Separation of the protein mix with eluents tested containing five concentrations of ACN with 0.1% TFA (**A**), 0.1% FA (**B**), 0.1% DFA (**C**), 0.05% TFA (**D**), 0.05% FA (**E**) or 0.05% DFA (**F**).*

Separation using different concentrations of IPA was tested next with acidifiers TFA and FA, see Figure S7. Again, addition of FA showed poor separation compared to TFA, where 0.1% FA had a better resolution between the eluting peaks in contrast to 0.05% FA. The 10% IPA concentration was sufficient for separation of the compounds in the protein mix with addition of TFA. IPA seemed to give a slightly better separation than ACN, however it did give unusual and broader peak shape of first two elution peaks. At higher concentrations of IPA, the last eluting peak even tended to split in two co-eluting peaks with bad peak shape. IPA significantly increases the viscosity of the mobile phase, which causes an elevation in the backpressure [8]. The SEC column used in this study has an optimum performance at a pressure below 140 bar, which was exceeded when using 20% IPA at flowrates of 0.3 ml/min or higher. Therefore, ACN was used further as organic solvent in the eluent.

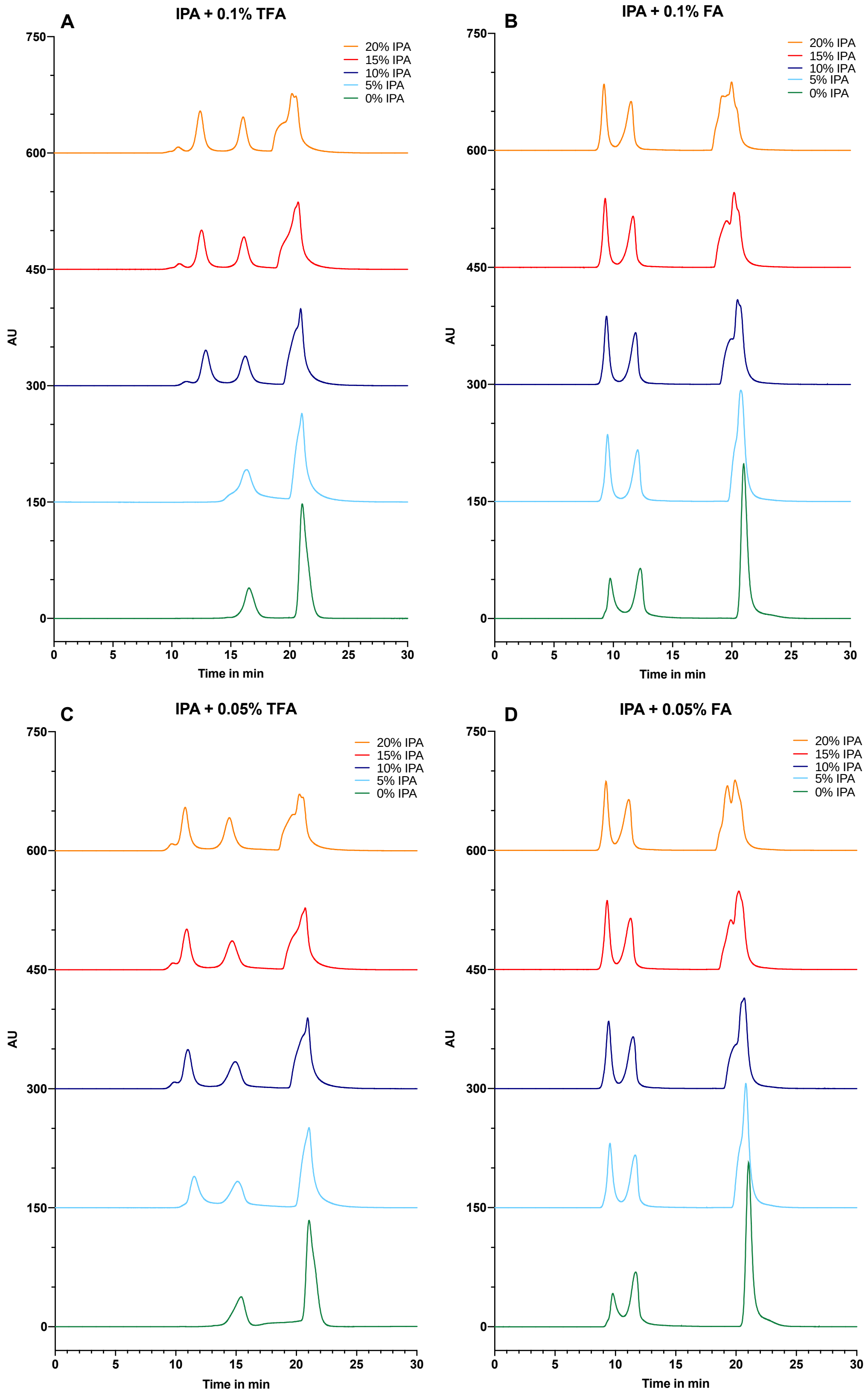


*Figure S7: Separation of the protein mix with eluents tested containing 5 concentrations of IPA and 0.1% TFA (A), 0.1% FA (B), 0.05% TFA (C) or 0.05% FA (D)*

## S3.4. SEC optimisation with venom

Since the combination of organic solvent with TFA in Milli-Q showed best results with PM separations, evaluation of separations with venom were restricted to these conditions. Venoms of *B. multicinctus* and *D. russelii*, were chosen for their complexity and known effects on coagulation modulation, see SI section S4 for their venom compositions. The largest toxins (SVMPs and SVSPs) are expected to elute first, followed by $PLA_2$s and ending with 3FTxs.

Separation of the venom from *B. multicinctus* showed similar results when different mobile phases were used. In Figure S8A-D, all chromatograms resulting from a total of 16 different mobile phase compositions tested for both organics combined with TFA at different concentrations are presented. In all chromatograms there are three clear main peaks visible, with an additional shoulder peak at the beginning of the first peak. Between the organic solvents IPA and ACN, better separation was observed with ACN. This was concluded due to an additional visible shoulder peak at RT 18-19 min shown in Figure S8A and at RT 17-18 min in Figure S8B. Whether this shoulder peak occurs, depends on the concentration of ACN in combination with the concentration of TFA added. For 0.1% TFA, this shoulder peak was visible at the range of 5% to 20% ACN, whilst when 0.05% TFA was added the peak disappeared at 20% ACN. When IPA was used as organic solvent, separation with 20% IPA again showed the peak splitting effect.

Analytical separations of venom from *D. russelii* evaluated with different mobile phase compositions were difficult to compare by only SEC-UV data. This is due to the many dissimilarities in retention time profiles, as shown in Figure S8E-H. Some peaks showed shifts in retention time with increasing organic solvent concentration in the mobile phase, including a peak eluting after the column volume time (thus the compound(s) representing this peak had secondary binding interactions with the SEC column material). The retention time of this specific peak decreased with increasing concentrations of organic eluent (indicating the reduction of possible secondary interactions with the column material). It is well known that separation of proteins by SEC can cause interactions with the SEC surface material due to hydrophobicity of some proteins [9]. A higher concentration of ACN in the mobile phase will cause these proteins to be better soluble in the mobile phase and interact less with the SEC material.

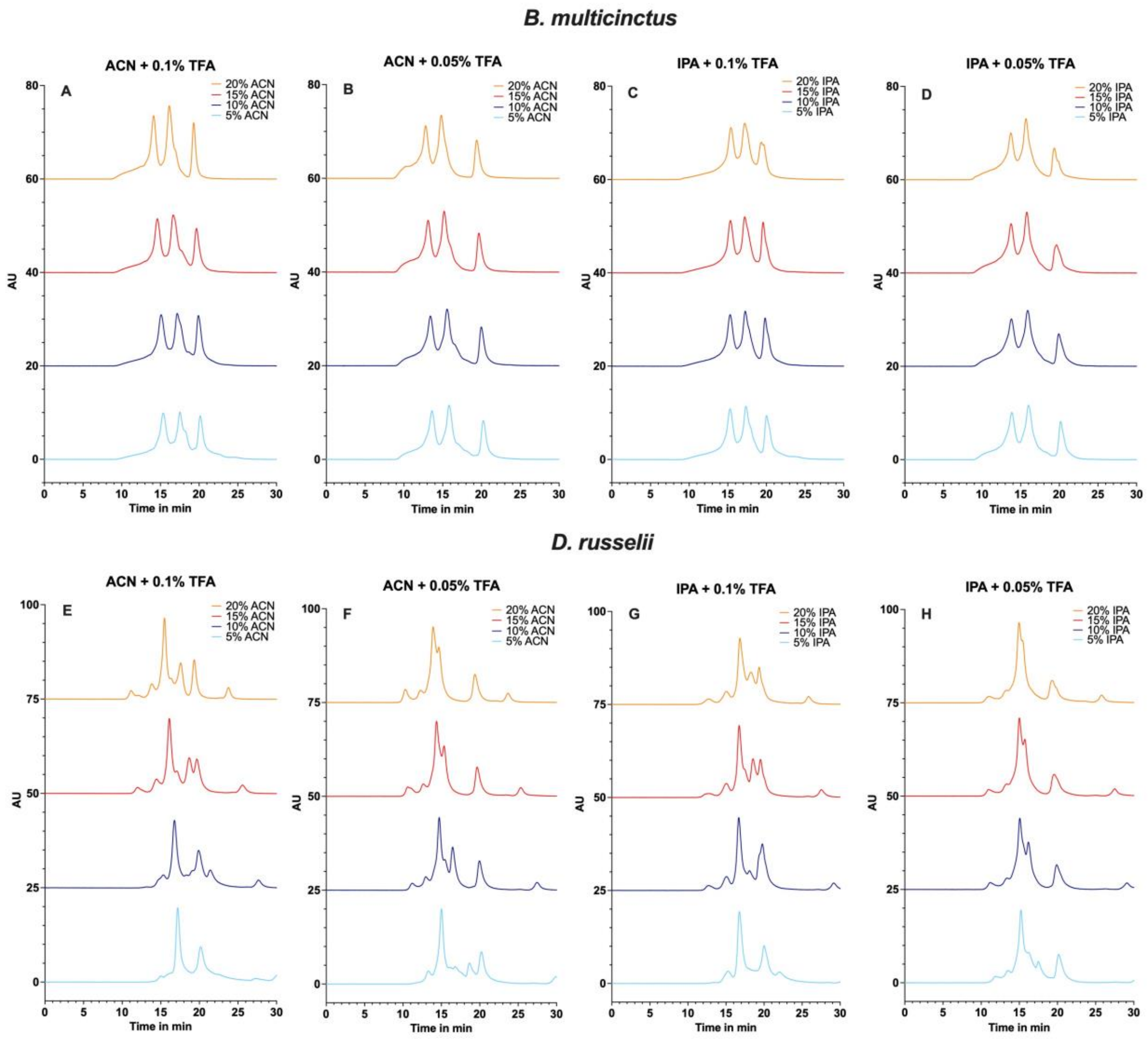


*Figure S8: SEC separation of B. multicinctus venom by 5-20% ACN + 0.1% TFA (A), 5-20% ACN + 0.05% TFA (B), 5-20% IPA +0.1% TFA (C) and 5-20% IPA + 0.05% TFA (D). SEC separation of D. russelii venom by 5-20% ACN + 0.1% TFA ©, 5-20% ACN + 0.05% TFA (F), 5-20% IPA + 0.1% TFA (G) and 5-20% IPA + 0.05% TFA (H). See SI document Prism S2 for all raw data.*

To also study the effect of DFA and FA for SEC separations of the two venoms, additional experiments were conducted. Results are given in Figure S9. Separations with 0.1% or 0.05% DFA gave similar results in combination with 10%, 15% and 20% ACN. Separations with FA as acidifier again showed less separation of which separation by 20% ACN + 0.05% FA gave the worst separation.

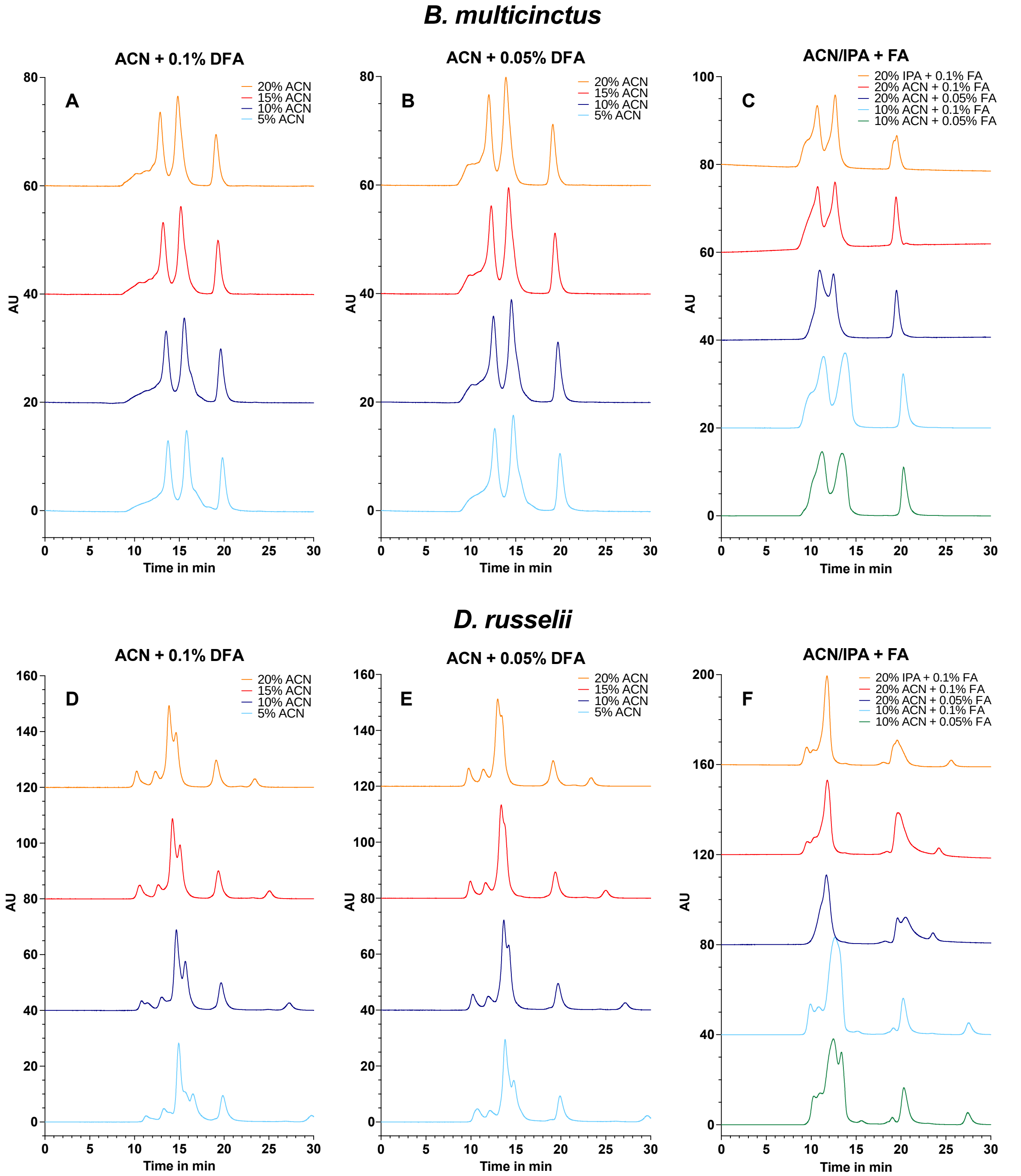


*Figure S9: Separation of 1 mg/ml venom from B. multicinctus and D. russelii by mobile phases containing different concentrations of ACN or IPA in combination with different concentrations of DFA or FA. See SI document Prism S2 for all raw data.*

A selection between the two organic solvents was made for follow-up research. Addition of IPA to the mobile phase increased the backpressure of the column significantly more than ACN. This phenomenon is likely the result of the high viscosity of IPA [8]. Castells *et al* 1997 [10] also suggested that the viscosity of IPA was responsible for broader an non-gaussian peaks. Due to the higher viscosity of using IPA in the mobile phase opposed to the lower viscosity of the injected samples, the peaks get instable and can therefore cause peak splitting. According to Goyon *et al* 2017 [8], IPA is supposed to be a better organic solvent to retain proteins from interacting with the SEC-column surface. However, in the specific case of venom from *D. russelii*, ACN assumingly better prevented proteins from interacting with the SEC material assumed by the shorter retention time of the last eluting peak. An explanation for the better performance of ACN is that this organic modifier enhances electrostatic

interactions and is therefore able to perturb ionic exchange [9,11]. Proteins with a large portion of basic amino acids will be protonated within the acidic conditions of the mobile phase and therefore can form secondary interactions with the hydrophilic film bonded silica surface of the SEC column. High concentrations of both ACN and IPA dissolved in water have the potency to unfold proteins [12]. Taking this into account, the lowest concentration of organic modifier which gives acceptable SEC separations was selected to avoid venom toxin denaturation. Since 10% ACN gave sufficient separation under all conditions tested, this concentration was chosen for further post-column bioassaying and MS analysis.

No initial selection was done for the tested acidifiers since their presence can also affect ionisation within the MS analysis and bioactivity of the toxins tested by bioassays. In addition, the three acidifiers FA, TFA and DFA in both concentrations of 0.05% and 0.1% were taken along to the next steps in this research.

## S4. Venom composition

Venoms of the species *D. russelii* and *B. multicinctus* were chosen because of their complexity and distinct differences in composition. In Figure S10, pie diagrams are given for the toxin composition of both venoms according to two studies from literature [13,14]. The venom of *B. multicinctus* contains mostly 3FTxs (around 65%) of which approximately 41% are classified as α-bungarotoxins and the others as κ-bungarotoxins and regular 3FTxs. Around 25% are ß-bungarotoxins which consist of an A- and B-chain, where the A-chain is classified as a $PLA_2$ and the B-chain as a kunitz type toxin. Other toxins include $PLA_2$s, C-type lectins and sometimes also SVMPs [13,15]. Venom of *D. russelii* is known for huge interspecies variation in venom composition. In general it mostly consists of a wide variation of $PLA_2$s, with a lower concentration of SVMPs, (Kunitz type) SVSPs, VEGF toxins and phosphodiesterases [14]. The relevant mass range of venom toxins in *D. russelii* venom is larger for than for *B. multicinctus* venom due to the higher abundance of SVMPs, which can have a mass up to 100 kDa. SVMPs are expected to elute first on SEC separations as they comprise the largest venom toxins in these venoms (i.e., a mass range of 20 to100 kDa), followed by or in combination with SVSPs with a mass range of 26 to 67 kDa [16,17]. ß-bungarotoxins have a mass range of approximately 20-21 kDa, $PLA_2$s are within the range of 13-15 kDa and the smallest toxin families are the 3FTxs and kunitz type toxins with masses around 7 kDa [18–21].

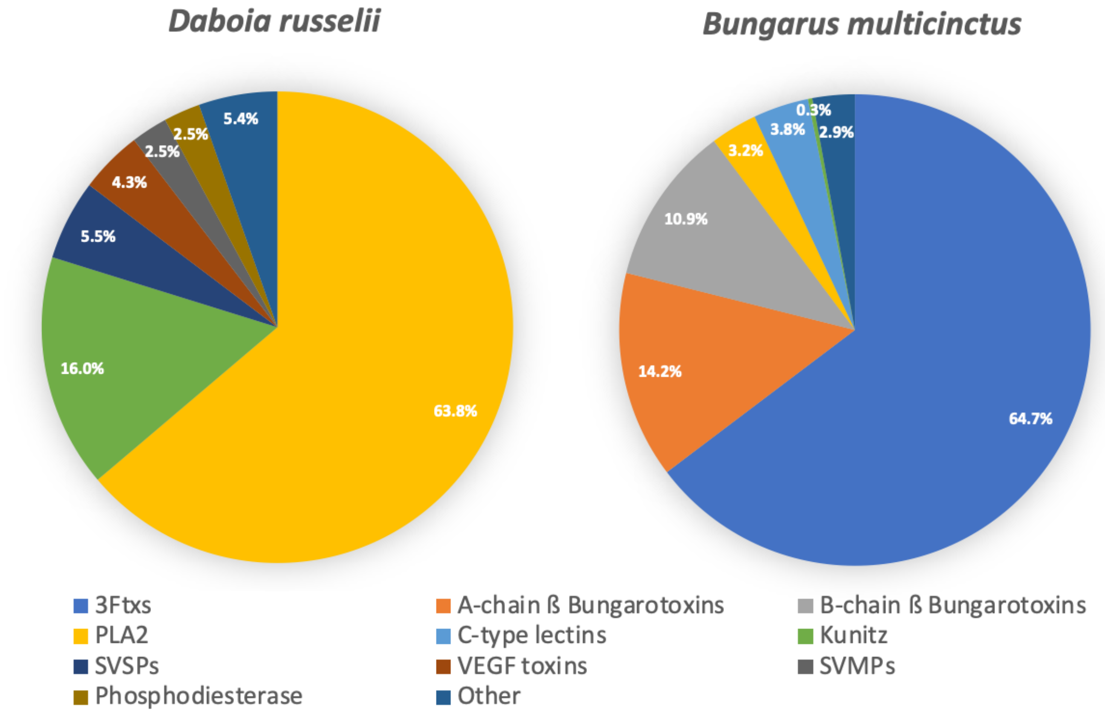


*Figure S10: The toxin distribution in venoms of D. russelii and B. multicinctus. 3FTx, three-finger toxin; $PLA_2$, phospholipase $A_2$; SVSP, snake venom serine protease; VEGF, vascular endothelial growth factor; SVMP, snake venom metalloproteinase* [13,14]

# S5. Proteomics Results

Tables S1 and S2 include all venom toxins that were identified by proteomics analysis from venoms of the species *Bungarus multicinctus* and *Daboia russelii*. The tables include the protein codes from the MASCOT database, name of the toxins, toxin family they belong to, exact masses retrieved from MASCOT (excluding modifications such as PTMs), the number of modifications (mod.) like glycosylation sites or PTMs and the sum of the protein scores, for each of the 6 mobile phases tested for SEC separation, for both venoms. In case of *D. russelii* venom, also the exact species connected to the MASCOT code, *Daboia russelii russelii* (DABRR) or *Daboia russelii siamensis* (DABSI), was added to the table.

*Table S1: Proteins identified for B. multicinctus venom separations. The proteins (i.e., venom toxins) were obtained through the MASCOT search engine from the Swissprot database and are all from one species, with code BUNMU. In the table the protein code, name, toxin family, exact mass derived from the peptide sequence and the amount of post translational modification (PTM) such as glycosylation's are displayed next to the sum of protein scores per separation. The Sum of protein scores is the sum of how many of the found peptides match with the sequence of the toxin. The more peptides match, the more likely it is that the matched protein is correct.*

| *Bungarus multicinctus* | | | | | *Sum of protein scores per separation* | | | | | |
|---|---|---|---|---|---|---|---|---|---|---|
| **Code** | **Name** | **Toxin Family** | **Exact mass** | **PTM** | **TFA 0.05** | **TFA 0.1** | **FA 0.05** | **FA 0.1** | **DFA 0.05** | **DFA 0.1** |
| *3L21A* | alpha-Bungarotoxin | 3FTxs | 7978.6 | | 24094 | 38223 | 34417 | 28563 | 31188 | 36039 |
| *3L21V* | alpha-Bungarotoxin Isoform V31 | 3FTxs | 8003.63 | | 25889 | 19475 | 28859 | 25780 | 23090 | 17378 |
| *3LK3* | Kappa-3-bungarotoxin | 3FTxs | 7368.22 | | 237 | 203 | 97 | 81 | 145 | |
| *3LK6* | Kappa-6-bungarotoxin | 3FTxs | 7365.29 | | 181 | 291 | 228 | 132 | 343 | 232 |
| *3LKB* | Kappa-bungarotoxin | 3FTxs | 7260.22 | | 276 | 133 | | | 259 | |
| *3NO41* | Neurotoxin BM10-1-like | 3FTxs | 7233.21 | | 36 | | | | | |
| *3NO4H* | Long neurotoxin homolog | 3FTxs | 7352.27 | | 798 | 1151 | 843 | 676 | 632 | 724 |
| *3NO52* | Long neurotoxin homolog NTL2 | 3FTxs | 7568.35 | | 230 | 258 | 363 | 364 | 304 | 413 |
| *3NO5I* | Gamma-bungarotoxin | 3FTxs | 7519.33 | | | 131 | | | | |
| *3NOH* | Toxin BMLCL | 3FTxs | 9012.78 | | 5336 | 6306 | 625 | 1203 | 1924 | 3194 |
| *3NOH3* | Cytotoxin-like protein TA-BMBGT3 | 3FTxs | 9111.86 | | 1496 | 1780 | 1211 | 945 | 1548 | 1928 |
| *3NOHE* | Muscarinic toxin BM14 | 3FTxs | 9068.89 | | 6189 | 5978 | 3148 | 3146 | 2589 | 5677 |
| *3SO3* | Short neurotoxin homolog NTL4 | 3FTxs | 7201.62 | | 2596 | 2786 | 1469 | 1986 | 2435 | 2984 |
| *3SO7* | Short neurotoxin homolog | 3FTxs | 7154.46 | | | 194 | | 222 | | 136 |
| *3SO93* | Neurotoxin-like protein pMD18-NTL3 | 3FTxs | 7468.36 | | 2114 | 2154 | 1930 | 1786 | 1612 | 1725 |
| *LECM2* | C-type lectin BML-2 | C-type lectin | 15749.44 | 1 | | 145 | | | 106 | |
| *OXLA* | L-amino-acid oxidase | Oxidoreduct. | 56836.01 | 2 | | 52 | 30 | 2384 | 5296 | 7399 |
| *PA2A* | Acidic $PLA_2$ | $PLA_2$ | 12808.56 | | 2873 | 1225 | 1380 | 1270 | 1019 | 821 |
| *PA2A4* | Acidic $PLA_2$ beta-bungarotoxin A4 chain | $PLA_2$ | 13394.88 | | 337 | 1714 | | 2537 | 739 | |
| *PA2A6* | Acidic $PLA_2$ beta-bungarotoxin A6 chain | $PLA_2$ | 13396.96 | | 30 | 53 | 80 | 89 | 31 | 61 |
| *PA2B* | Basic $PLA_2$ beta-bungarotoxin A-AL2 chain | $PLA_2$ | 13575.11 | | 18165 | 23381 | 7811 | 18686 | 12209 | 14253 |
| *PA2B1* | Basic $PLA_2$ beta-bungarotoxin A1 chain | $PLA_2$ | 13468.01 | | 12301 | 1938 | 9001 | 2955 | 3129 | 1240 |
| *PA2B2* | Basic $PLA_2$ beta-bungarotoxin A2 chain | $PLA_2$ | 13656.2 | | 21525 | 10835 | 8566 | 6858 | 4033 | |
| *PA2B5* | Basic $PLA_2$ beta-bungarotoxin A5 chain | $PLA_2$ | 13559.07 | | 29 | 381 | | 31 | | 62 |
| *PA2B7* | Basic $PLA_2$ beta-bungarotoxin A7 chain | $PLA_2$ | 13445.08 | | 615 | 1771 | 1313 | 1382 | 405 | 651 |
| *PA2BA* | Basic $PLA_2$ beta-bungarotoxin A-AL1 chain | $PLA_2$ | 13472.08 | | 27116 | 39265 | 14669 | 28957 | 23492 | 31146 |
| *PA2BC* | Basic $PLA_2$ beta-bungarotoxin A-AL3 chain | $PLA_2$ | 13966.33 | | 35 | | | | | |
| *VKTH1* | Kunitz-type serine protease inhibitor homolog beta-bungarotoxin B1 chain | Kunitz-type | 7177.34 | | 1312 | 148 | 403 | | 438 | 174 |
| *VKTH2* | Kunitz-type serine protease inhibitor homolog beta-bungarotoxin B2 chain | Kunitz-type | 7186.37 | | 718 | | 331 | | 650 | 93 |
| *VKTH3* | Kunitz-type serine protease inhibitor homolog beta-bungarotoxin B3 chain | Kunitz-type | 7205.28 | | 2422 | | | | 712 | 70 |
| *VM3* | Zinc metalloproteinase-disintegrin-like BmMP | SVMP | 47743.54 | 1 | 235 | 1103 | | 889 | 1879 | 1872 |
| *VNP* | Natriuretic peptide BM026 | Natriuretic peptide | 4943.54 | 2 | 37 | 949 | 39 | | 35 | 39 |
| | | *Total detected toxins per separation* | | | ***28*** | ***28*** | ***22*** | ***23*** | ***27*** | ***24*** |

*Table S2: Proteins identified for D. russelii venom separations. The proteins (i.e., venom toxins) were obtained through the MASCOT search engine from the Swissprot database and are majorly from two species, the Daboia russelii russelii (DABRR) and the Daboia russelii siamensis (DABSI). In the table the protein code, name, toxin family, exact mass derived from the peptide sequence and the amount of post translational modification (PTM) such as glycosylation's are displayed next to the sum of protein scores per separation. The Sum of protein scores is the sum of how many of the found peptides match with the sequence of the toxin. The more peptides match, the more likely it is that the matched protein is correct.*

| *Daboia russelii* | | | | | | *Sum of protein scores per separation* | | | | | |
|---|---|---|---|---|---|---|---|---|---|---|---|
| ***Species*** | ***Code*** | ***Name*** | ***Toxin Family*** | ***Exact mass*** | ***PTM*** | ***TFA 0.05*** | ***TFA 0.1*** | ***FA 0.05*** | ***FA 0.1*** | ***DFA 0.05*** | ***DFA 0.1*** |
| *DABRR* | NGFV | Venom nerve growth factor | NGF-beta | 13,268.32 | 1 | | | | | 31 | |
| *DABRR* | OXLA | L-amino-acid oxidase | Oxidoreduct. | 54,826.05 | 2 | 4338 | | 1083 | 12991 | 21070 | 25120 |
| *DABSI* | OXLA | L-amino-acid oxidase | Oxidoreduct. | 46,338.57 | 2 | 855 | | 53 | | | |
| *DABSI* | PA2A | Acidic $PLA_2$ Drs-$PLA_2$ | $PLA_2$ | 2,012.07 | | | 25 | | | | |
| *DABSI* | PA2A7 | Acidic $PLA_2$ RV-7 | $PLA_2$ | 13,655.72 | | 1082 | 1442 | 8290 | 7610 | 3778 | 3494 |
| *DABRR* | PA2B | Basic $PLA_2$ RVV-VD | $PLA_2$ | 13,602.94 | | | 105 | | | | |
| *DABRR* | PA2B1 | Basic $PLA_2$ Drk-b1 | $PLA_2$ | 14,066.30 | | | | | | | 76 |
| *DABSI* | PA2B1 | Basic $PLA_2$ DsM-b1/DsM-b1' | $PLA_2$ | 14,045.35 | | 83 | 89 | | 370 | 23 | 336 |
| *DABRR* | PA2B3 | Basic $PLA_2$ 3 | $PLA_2$ | 13,663.18 | | 68786 | 90425 | 40483 | 33435 | 40909 | 46041 |
| *DABSI* | PA2B4 | Basic $PLA_2$ RV-4 | $PLA_2$ | 13,789.20 | | 2721 | 1952 | | 3284 | 89 | 2274 |
| *DABRR* | PA2B5 | Basic $PLA_2$ VRV-PL-V | $PLA_2$ | 13,563.13 | | 62957 | 66232 | 51720 | 40805 | 47877 | 51458 |
| *DABRR* | PA2B8 | Basic $PLA_2$ VRV-PL-VIIIa | $PLA_2$ | 13,587.20 | | 36376 | 39843 | 30315 | 22701 | 25927 | 32780 |
| *DABSI* | PA2BS | Basic $PLA_2$ DsM-S1 | $PLA_2$ | 13,615.21 | | 413 | 6088 | | | 7653 | 1786 |
| *DABSI* | SL3 | Snaclec 3 | Snaclec | 14,481.62 | | | | 1870 | 1847 | 1160 | 111 |
| *DABSI* | SL4 | Snaclec 4 | Snaclec | 14,368.69 | | | | 1523 | 1286 | 667 | 374 |
| *DABSI* | SL5 | Snaclec 5 | Snaclec | 14,727.86 | | | | 412 | 166 | 62 | |
| *DABSI* | SL6 | Snaclec 6 | Snaclec | 14,227.80 | | 78 | | 35 | | 31 | |
| *DABSI* | SL7 | Snaclec 7 | Snaclec | 15,696.16 | | 2975 | | 773 | 715 | 6395 | 2779 |
| *DABSI* | SLA | Snaclec dabocetin subunit alpha | Snaclec | 15,150.20 | | | | 29 | 64 | 155 | |
| *DABSI* | SLLC1 | Snaclec coagulation factor X-activating enzyme light chain 1 | Snaclec | 14,452.80 | 1 | 426 | 137 | | | 60 | 272 |
| *DABSI* | SLLC2 | Snaclec coagulation factor X-activating enzyme light chain 2 | Snaclec | 15,922.45 | 1 | 85 | 38 | 57 | 76 | 37 | 56 |
| *DABRR* | TXVE | Snake venom vascular endothelial growth factor toxin VR-1 | VEGF | 12,539.92 | 1 | 3884 | 4951 | 2846 | 2603 | 5508 | 5041 |
| *DABSI* | TXVE | Snake venom vascular endothelial growth factor toxin VR-1 | VEGF | 12,539.92 | 1 | 1297 | 327 | | | | 212 |
| *DABRR* | VKT4 | Kunitz-type serine protease inhibitor 4 | Kunitz | 6,696.99 | 1 | | | | 176 | | 349 |
| *DABSI* | VKTB1 | Kunitz-type serine protease inhibitor B1 | Kunitz | 6,869.09 | | 463 | 277 | 293 | 500 | 642 | 1147 |
| *DABSI* | VKTC1 | Kunitz-type serine protease inhibitor C1 | Kunitz | 6,866.13 | 1 | 39 | | 34 | 76 | 82 | 170 |
| *DABSI* | VKTC3 | Kunitz-type serine protease inhibitor C3 | Kunitz | 6,994.14 | | 80 | | 134 | 66 | 518 | 876 |
| *DABSI* | VM3CX | Coagulation factor X-activating enzyme heavy chain | SVMP | 48,125.17 | 4 | 3959 | 4375 | 1323 | 1319 | 1630 | 2821 |
| *DABSI* | VSPA | Factor V activator RVV-V alpha | SVSP | 26,153.10 | 1 | 2956 | 872 | 1210 | 418 | 2578 | 3103 |
| *DABSI* | VSPAF | Alpha-fibrinogenase-like | SVSP | 25,782.81 | 1 | 1607 | 1439 | 842 | 863 | 1844 | 1711 |
| *DABSI* | VSPB | Beta-fibrinogenase-like | SVSP | 25,350.33 | 4 | 2721 | 3544 | 2098 | 1133 | 1865 | 2317 |
| *DABSI* | VSPG | actor V activator RVV-V gamma | SVSP | 26,138.03 | 1 | 829 | 1789 | | 1209 | 562 | |
| | | | | *Total detected toxins per separation* | | ***23*** | ***19*** | ***21*** | ***23*** | ***27*** | ***24*** |

All proteins have a protein score chromatogram (PSC) available for each measurement. All EICs per fractionated plate are given in Figure S11 for *B. multicinctus* and Figure S12 for *D. russelii*. The data was plotted in Prism, see SI documents Prism S3-S8 for *B. multicinctus* and Prism S9-S14 for *D. russelii*.

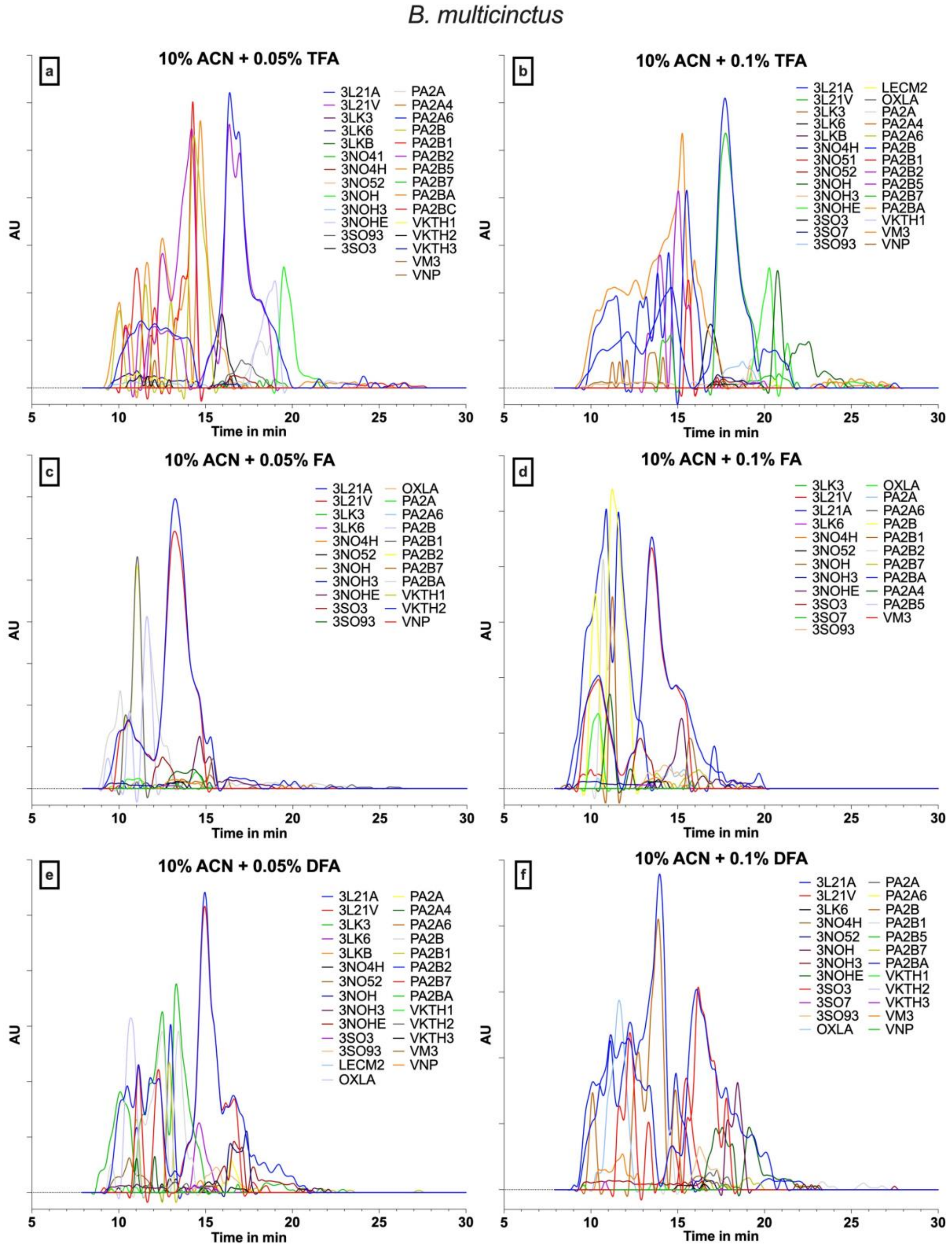


*Figure S11: A visualization of the PSCs from the identified proteins in the proteomics analysis per sample for the B. multicinctus venom. Proteins identified in the fractionated plates from separation using 10% ACN + 0.05% TFA (a), 10% ACN + 0.1% TFA (b), 10% ACN + 0.05% FA (c), 10% ACN + 0.1% FA (d), 10% ACN + 0.05% DFA (e) and 10% ACN + 0.1% DFA (f).*

*D. russelii*

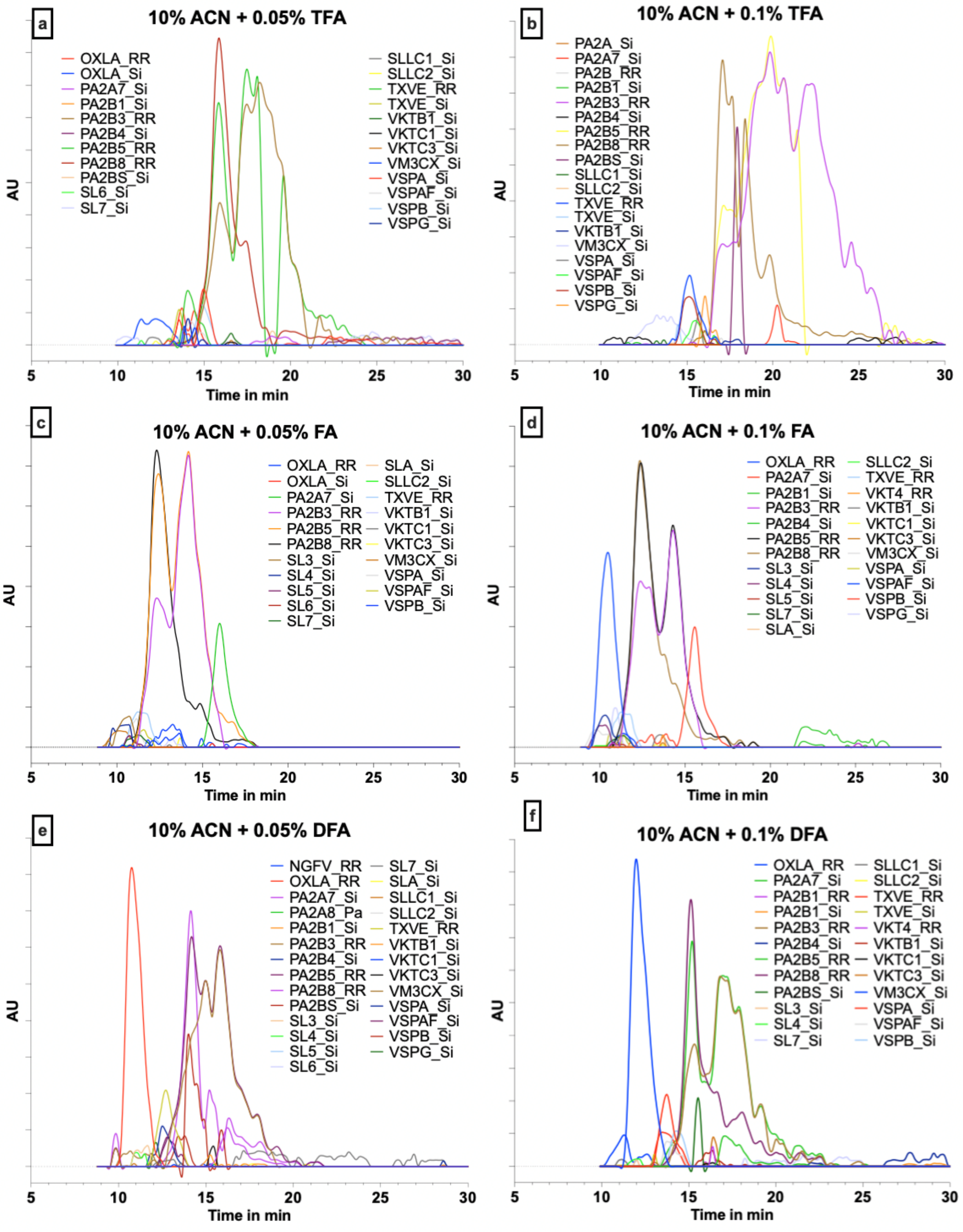


*Figure S12: A visualization of the PSCs from the identified proteins in the proteomics analysis per sample for the D. russelii venom. Proteins identified in the fractionated plates from separation using 10% ACN + 0.05% TFA (a), 10% ACN + 0.1% TFA (b), 10% ACN + 0.05% FA (c), 10% ACN + 0.1% FA (d), 10% ACN + 0.05% DFA (e) and 10% ACN + 0.1% DFA (f).*

## S6. Plasma coagulation assay results

Plasma coagulation assay data for venom of *B. multicinctus*, separated with SEC using 6 different mobile phase compositions are shown in Figure S13. The UV data using 280 nm is shown in black with the absorption unit (AU) on the left y-axis. Anticoagulation is shown in blue with the absorption intensity on the right y-axis. The procoagulation is shown in red with the average rate on the right y-axis. The data was plotted in Prism, see SI documents Prism S3-S8 for the raw data.

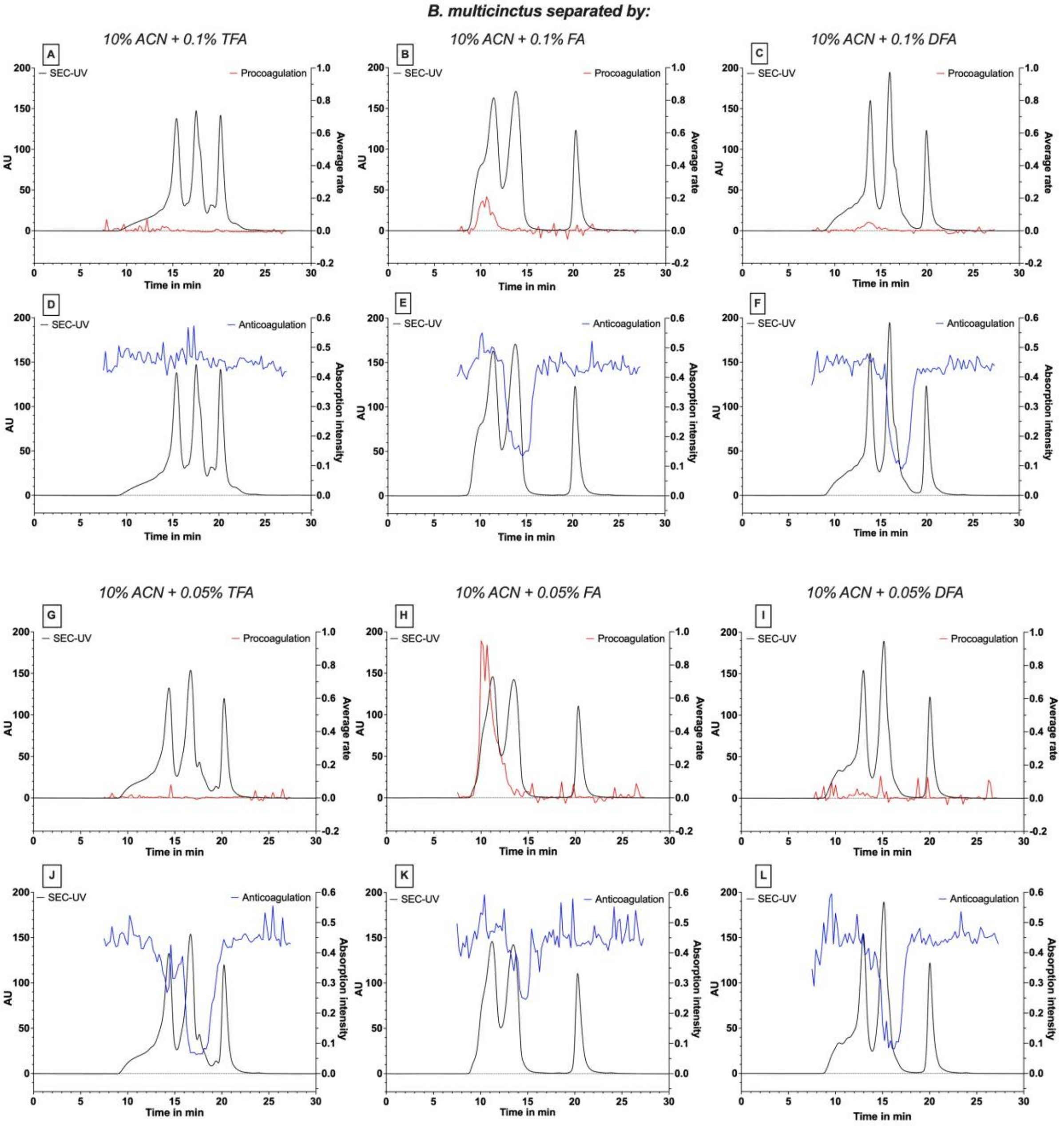


*Figure S13: Plasma coagulation bioassay results of B. multicinctus venom after SEC separation, presented as bioassay chromatograms. Pro- and anticoagulation results for SEC separation using 10% ACN + 0.1% TFA (A+D), 10% ACN + 0.1% FA (B+E), 10% ACN + 0.1% DFA (C+F), 10% ACN + 0.05% TFA (G+J), 10% ACN + 0.05% FA (H+K) and 10% ACN + 0.05% DFA (I+L). as eluent.*

Plasma coagulation assay data for venom of *D. russelii*, separated by SEC using 6 different mobile phase compositions are shown in Figure S14. The UV data using 280 nm is shown in black with the absorption unit (AU) on the left y-axis. Anticoagulation is shown in blue with the absorption intensity

on the right y-axis. The procoagulation is shown in red with the average rate on the right y-axis. The data was plotted in Prism, see SI documents Prism S9-S14 for the raw data.

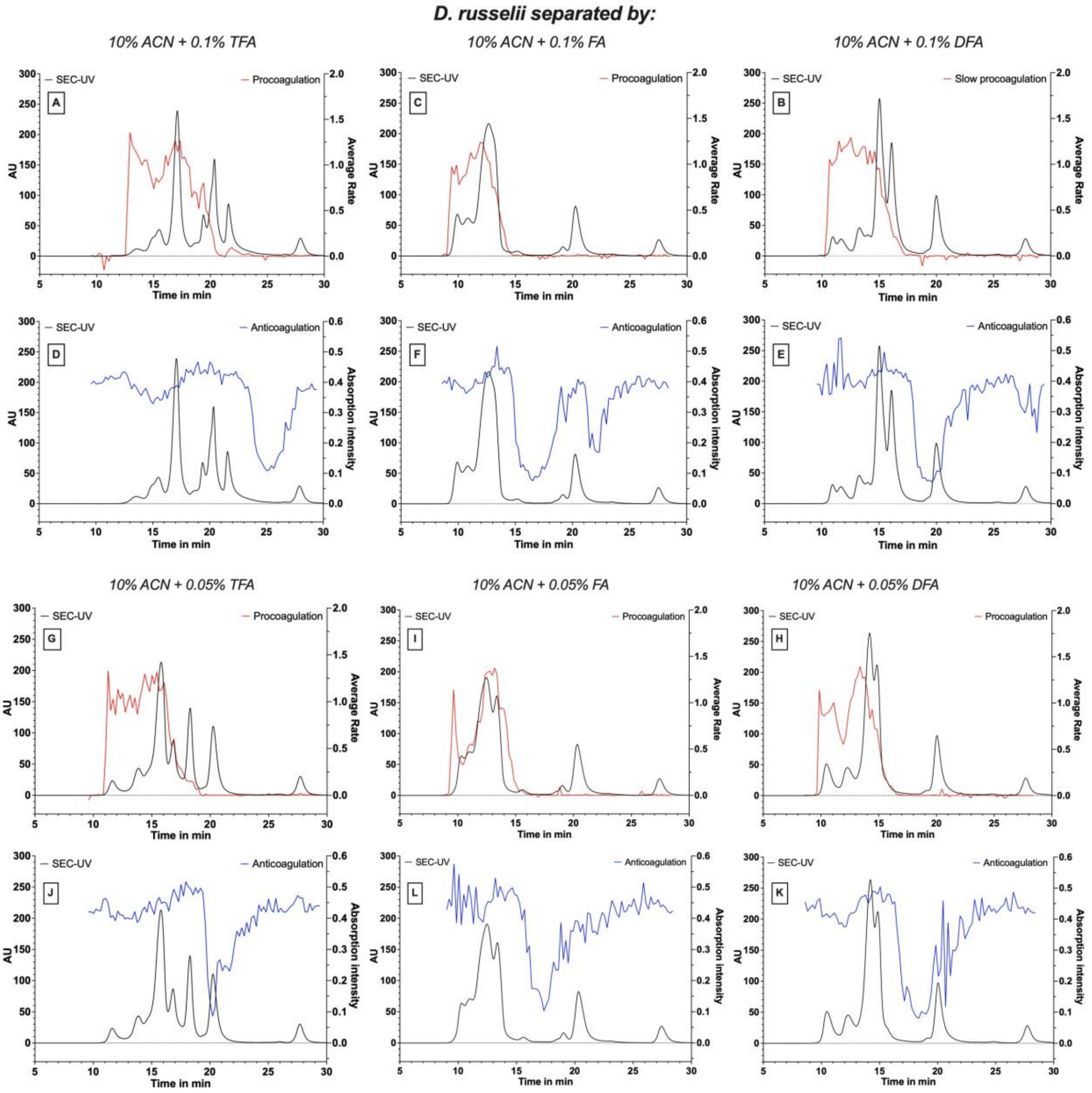


*Figure S14: Plasma coagulation bioassay results of* D. russelii *venom after SEC separation, presented as bioassay chromatograms. Pro- and anticoagulation results for SEC separation using 10% ACN + 0.1% TFA (A+D), 10% ACN + 0.1% FA (B+E), 10% ACN + 0.1% DFA (C+F), 10% ACN + 0.05% TFA (G+J), 10% ACN + 0.05% FA (H+K) and 10% ACN + 0.05% DFA (I+L) as eluent.*

An overlay was made of the bioassay chromatogram data (with and without marimastat added to the bioassay), UV data and proteomics data of *D. russelii* venom SEC-separated using mobile phase of 10% ACN and 0.05% DFA (See Figure S15). This overlay shows which toxins elute at the same time as that the anti- and procoagulation were detected.

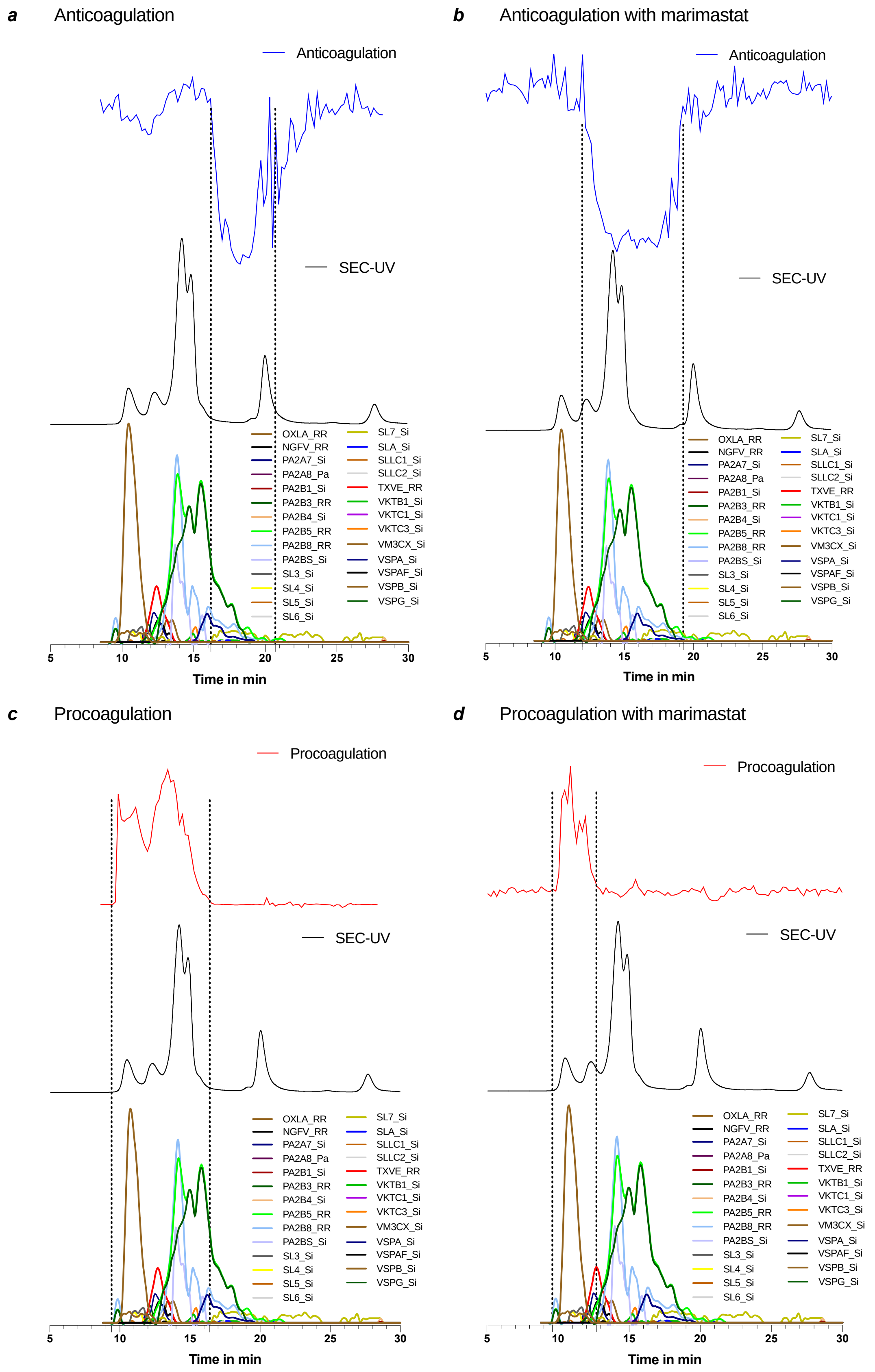


*Figure S15: Overlay of the bioassay, UV and proteomics data of D. russelii venom. (a) anticoagulation with the regular assay, (b) anticoagulation with assay including marimastat, (c) procoagulation with the regular assay and (d) procoagulation with the assay including marimastat.*

# S7. SEC-MS raw data

Raw data including all deconvoluted masses, the TICs and EICs of the SEC-MS measurements for both venoms are represented in this SI. The TICs and EICs are visualised in PowerPoint documents, see documents PP S1 for *B. multicinctus* and PP S2 for *D. russelii*.

## S7.1.Deconvolution Data

Each table contains deconvolution data for both venoms from the separation by:

- Table S3: 10% ACN + 0.1% TFA
- Table S4: 10% ACN + 0.05% TFA
- Table S5: 10% ACN + 0.1% FA
- Table S6: 10% ACN + 0.05% FA
- Table S7: 10% ACN + 0.1% DFA
- Table S8: 10% ACN + 0.05% DFA

*Table S3: A list of all deconvoluted m/z values from the MS data from the SEC separation using 10% ACN + 0.1% TFA as the mobile phase. The delay of 1.2 min between the HPLC and MS systems is processed in the retention time (RT).*

| **MS analysis of separation by 10% ACN + 0.1% TFA** | | | | | | | | | |
|---|---|---|---|---|---|---|---|---|---|
| *Bungarus multicinctus* | | | | | *Daboia russelii* | | | | |
| *m/z* | Charge | Mass | Intensity | RT | *m/z* | Charge | Mass | Intensity | RT |
| 2299.5728 | 9 | 20692.54 | 10477 | 13.8 - 16.5 | 2276.0485 | 11 | 25029.02 | 2357 | 14.4-16.1 |
| 2306.7966 | 9 | 20744.04 | 10133 | 13.8 - 16.5 | 2471.3139 | 10 | 24708.68 | 2263 | 14.4-16.1 |
| 2309.6844 | 9 | 20766.03 | 8746 | 13.8 - 16.5 | 1943.3013 | 7 | 13598.03 | 290545 | 16.2-18.7 |
| 2301.2355 | 9 | 20702.05 | 6626 | 13.8 - 16.5 | 1675.3732 | 8 | 13397.81 | 37761 | 16.2-18.7 |
| 2293.5656 | 9 | 20639.05 | 3996 | 13.8 - 16.5 | 1938.0047 | 6 | 11624.26 | 35092 | 16.2-18.7 |
| 2327.9225 | 9 | 20934.13 | 2520 | 13.8 - 16.5 | 2269.8471 | 6 | 13613.49 | 28749 | 16.2-18.7 |
| 1597.706 | 5 | 7985.11 | 461366 | 16.6 - 19.4 | 1941.0105 | 7 | 13590.85 | 20373 | 16.2-18.7 |
| 1603.3099 | 5 | 8013.57 | 156205 | 16.6 - 19.4 | 1661.2399 | 8 | 13284.43 | 18801 | 16.2-18.7 |
| 1502.0522 | 5 | 7506.71 | 56762 | 16.6 - 19.4 | 3400.0219 | 8 | 27195.02 | 15173 | 16.2-18.7 |
| 1600.9033 | 5 | 8004.36 | 43791 | 16.6 - 19.4 | 2273.344 | 6 | 13637.68 | 9882 | 16.2-18.7 |
| 1328.5852 | 6 | 7967.80 | 31582 | 16.6 - 19.4 | 1645.1081 | 8 | 13155.45 | 9780 | 16.2-18.7 |
| 1472.2395 | 5 | 7358.38 | 30992 | 16.6 - 19.4 | 1921.829 | 6 | 11527.15 | 7242 | 16.2-18.7 |
| 1606.5072 | 5 | 8029.89 | 26188 | 16.6 - 19.4 | 1781.496 | 7 | 12465.59 | 6501 | 16.2-18.7 |
| 1475.6397 | 5 | 7374.11 | 25545 | 16.6 - 19.4 | 1797.6509 | 7 | 12578.68 | 6337 | 16.2-18.7 |
| 1505.6517 | 5 | 7524.79 | 24079 | 16.6 - 19.4 | 1950.2932 | 7 | 13646.74 | 23020 | 18.9-20.1 |
| 1817.2831 | 4 | 7267.67 | 18402 | 16.6 - 19.4 | 2278.6692 | 6 | 13669.99 | 7813 | 18.9-20.1 |
| 1609.7041 | 5 | 8045.34 | 13877 | 16.6 - 19.4 | 2283.6694 | 6 | 13700.09 | 6847 | 18.9-20.1 |
| 1591.3068 | 5 | 7954.30 | 10639 | 16.6 - 19.4 | 2286.1627 | 6 | 13714.97 | 5790 | 18.9-20.1 |
| 1509.2511 | 5 | 7542.92 | 8605 | 16.6 - 19.4 | 1887.2606 | 7 | 13204.48 | 5098 | 18.9-20.1 |
| 1461.2286 | 5 | 7302.74 | 8527 | 16.6 - 19.4 | 1921.7052 | 7 | 13446.94 | 4692 | 18.9-20.1 |
| 1324.2508 | 6 | 7942.81 | 8100 | 16.6 - 19.4 | 1865.964 | 7 | 13057.28 | 4180 | 18.9-20.1 |
| 2016.8623 | 4 | 8065.75 | 7156 | 16.6 - 19.4 | 1905.6975 | 7 | 13334.24 | 3888 | 18.9-20.1 |
| 1432.6773 | 5 | 7160.31 | 6948 | 16.6 - 19.4 | 1952.1508 | 7 | 13659.72 | 36240 | 20.2-21.3 |
| 1455.6283 | 5 | 7276.03 | 7584 | 16.6 - 19.4 | 1888.831 | 7 | 13217.60 | 6536 | 20.2-21.3 |
| 1586.098 | 5 | 7928.35 | 5882 | 16.6 - 19.4 | 1923.5616 | 7 | 13459.95 | 6356 | 20.2-21.3 |
| 1312.5748 | 6 | 7872.72 | 5197 | 16.6 - 19.4 | 1907.2644 | 7 | 13346.76 | 5352 | 20.2-21.3 |
| 1498.6474 | 5 | 7490.46 | 4953 | 16.6 - 19.4 | 1867.8196 | 7 | 13070.12 | 4987 | 20.2-21.3 |
| 1466.6364 | 5 | 7331.86 | 4856 | 16.6 - 19.4 | 1952.1486 | 7 | 13660.15 | 87564 | 21.3-24.2 |
| 1315.2439 | 6 | 7888.02 | 4624 | 16.6 - 19.4 | 1888.8278 | 7 | 13217.45 | 13223 | 21.3-24.2 |
| 1815.7536 | 5 | 9075.89 | 10292 | 19.7 - 21.5 | 1923.5587 | 7 | 13459.96 | 12670 | 21.3-24.2 |
| 1812.3538 | 5 | 9058.65 | 8887 | 19.7 - 21.5 | 2280.1671 | 6 | 13677.23 | 11527 | 21.3-24.2 |
| 1818.9556 | 5 | 9092.58 | 4905 | 19.7 - 21.5 | 1907.4051 | 7 | 13347.09 | 10412 | 21.3-24.2 |
| 1804.7292 | 5 | 9021.24 | 2002 | 19.7 - 21.5 | 1867.817 | 7 | 13070.48 | 10214 | 21.3-24.2 |
| 1807.7368 | 5 | 9037.18 | 1718 | 19.7 - 21.5 | 2283.8297 | 6 | 13699.64 | 7684 | 21.3-24.2 |
| 2280.1812 | 4 | 9119.06 | 1524 | 19.7 - 21.5 | 1950.0042 | 7 | 13654.44 | 7637 | 21.3-24.2 |
| 1804.5334 | 5 | 9019.90 | 5035 | 21.7 - 23.6 | | | | | |

| | | | | | | | | | |
|---|---|---|---|---|---|---|---|---|---|
| 1801.3314 | 5 | 9002.96 | 4218 | 21.7 - 23.6 | | | | | |
| 1807.531 | 5 | 9036.50 | 2069 | 21.7 - 23.6 | | | | | |

*Table S4: A list of all deconvoluted m/z values from the MS data from the SEC separation using 10% ACN + 0.05% TFA as the mobile phase. The delay of 1.2 min between the HPLC and MS systems is processed in the retention time (RT).*

| **MS analysis of separation by 10% ACN + 0.5% TFA** | | | | | | | | | |
|---|---|---|---|---|---|---|---|---|---|
| *Bungarus multicinctus* | | | | | *Daboia russelii* | | | | |
| *m/z* | Charge | Mass | Intensity | RT | *m/z* | Charge | Mass | Intensity | RT |
| 2069.696 | 10 | 20692.26 | 17918 | 12.9 - 15.3 | 2471.2931 | 10 | 24708.63 | 6845 | 12.7-14.5 |
| 2075.0981 | 10 | 20746.75 | 17226 | 12.9 - 15.3 | 2275.9359 | 11 | 25028.79 | 4860 | 12.7-14.5 |
| 2078.795 | 10 | 20781.29 | 15857 | 12.9 - 15.3 | 2270.2137 | 11 | 24970.81 | 2651 | 12.7-14.5 |
| 2064.5893 | 10 | 20641.08 | 6515 | 12.9 - 15.3 | 2600.0398 | 11 | 28589.36 | 1634 | 12.7-14.5 |
| 2300.9931 | 9 | 20716.39 | 10591 | 12.9 - 15.3 | 2613.4133 | 11 | 28736.47 | 1570 | 12.7-14.5 |
| 2080.3946 | 10 | 20804.51 | 6390 | 12.9 - 15.3 | 1943.139 | 7 | 13597.59 | 337306 | 14.7-16.7 |
| 2088.3098 | 10 | 20878.03 | 4825 | 12.9 - 15.3 | 1675.3565 | 8 | 13397.66 | 38470 | 14.7-16.7 |
| 2095.0144 | 10 | 20939.10 | 4346 | 12.9 - 15.3 | 1945.5644 | 7 | 13612.91 | 30387 | 14.7-16.7 |
| 2093.7154 | 10 | 20927.08 | 4006 | 12.9 - 15.3 | 1660.979 | 8 | 13283.65 | 23977 | 14.7-16.7 |
| 1997.2383 | 4 | 7986.99 | 2934 | 12.9 - 15.3 | 1698.3678 | 8 | 13590.72 | 22882 | 14.7-16.7 |
| 1597.6897 | 5 | 7985.02 | 609885 | 15.4 - 17.6 | 1937.9851 | 6 | 11623.46 | 30881 | 14.7-16.7 |
| 1603.2942 | 5 | 8013.46 | 193664 | 15.4 - 17.6 | 3022.3211 | 9 | 27196.59 | 10527 | 14.7-16.7 |
| 1501.8375 | 5 | 7506.54 | 66192 | 15.4 - 17.6 | 1644.9664 | 8 | 13154.80 | 9048 | 14.7-16.7 |
| 1600.8867 | 5 | 8004.20 | 54591 | 15.4 - 17.6 | 2273.4896 | 6 | 13629.77 | 7567 | 14.7-16.7 |
| 1328.5712 | 6 | 7967.66 | 50668 | 15.4 - 17.6 | 1693.1145 | 8 | 13543.44 | 5311 | 14.7-16.7 |
| 1472.4244 | 5 | 7358.59 | 41829 | 15.4 - 17.6 | 1952.5632 | 7 | 13660.77 | 7218 | 14.7-16.7 |
| 1475.4238 | 5 | 7373.87 | 35817 | 15.4 - 17.6 | 1689.4908 | 8 | 13512.04 | 5833 | 14.7-16.7 |
| 1817.2656 | 4 | 7267.49 | 34063 | 15.4 - 17.6 | 1797.7735 | 7 | 12578.85 | 5636 | 14.7-16.7 |
| 1505.6364 | 5 | 7524.76 | 33304 | 15.4 - 17.6 | 1781.6207 | 7 | 12465.76 | 5354 | 14.7-16.7 |
| 1606.691 | 5 | 8030.35 | 30092 | 15.4 - 17.6 | 1673.4788 | 8 | 13386.00 | 4825 | 14.7-16.7 |
| 1609.6884 | 5 | 8045.40 | 15700 | 15.4 - 17.6 | 1687.1143 | 8 | 13493.66 | 4426 | 14.7-16.7 |
| 1326.5736 | 6 | 7956.61 | 15388 | 15.4 - 17.6 | 1907.3025 | 6 | 11439.93 | 3622 | 14.7-16.7 |
| 1461.2139 | 5 | 7302.79 | 15556 | 15.4 - 17.6 | 1950.127 | 7 | 13646.16 | 39312 | 16.8-17.8 |
| 1324.0709 | 6 | 7941.60 | 13963 | 15.4 - 17.6 | 1887.0921 | 7 | 13203.72 | 9419 | 16.8-17.8 |
| 1509.2359 | 5 | 7542.17 | 12930 | 15.4 - 17.6 | 1921.8271 | 7 | 13446.08 | 7551 | 16.8-17.8 |
| 1432.8647 | 5 | 7160.25 | 11286 | 15.4 - 17.6 | 1380.985 | 5 | 6900.93 | 7244 | 16.8-17.8 |
| 1455.6123 | 5 | 7275.37 | 12695 | 15.4 - 17.6 | 1865.942 | 7 | 13056.65 | 7041 | 16.8-17.8 |
| 1312.7281 | 6 | 7873.23 | 8077 | 15.4 - 17.6 | 1905.5281 | 7 | 13333.87 | 6365 | 16.8-17.8 |
| 1315.0646 | 6 | 7887.05 | 7627 | 15.4 - 17.6 | 1909.6624 | 7 | 13362.91 | 5946 | 16.8-17.8 |
| 1466.6217 | 5 | 7331.60 | 7330 | 15.4 - 17.6 | 1925.6809 | 7 | 13475.70 | 5681 | 16.8-17.8 |
| 1586.0826 | 5 | 7928.31 | 7213 | 15.4 - 17.6 | 1890.9469 | 7 | 13233.36 | 4933 | 16.8-17.8 |
| 1498.6337 | 5 | 7490.41 | 6443 | 15.4 - 17.6 | 1681.4727 | 8 | 13475.95 | 4175 | 16.8-17.8 |
| 1317.0659 | 6 | 7899.08 | 6135 | 15.4 - 17.6 | 1870.3649 | 7 | 13086.81 | 3428 | 16.8-17.8 |
| 1503.761 | 6 | 9020.06 | 3804 | 17.7 - 19.1 | 1947.9838 | 7 | 13635.14 | 4931 | 16.8-17.8 |
| 1506.591 | 6 | 9038.75 | 3090 | 17.7 - 19.1 | 1400.8043 | 5 | 7000.28 | 2957 | 16.8-17.8 |
| 1801.3167 | 5 | 9002.17 | 3144 | 19.4 - 21.5 | 1902.8106 | 7 | 13318.58 | 2925 | 16.8-17.8 |
| | | | | | 1385.5783 | 3 | 4156.02 | 2755 | 16.8-17.8 |
| | | | | | 1370.9155 | 3 | 4112.04 | 2650 | 16.8-17.8 |
| | | | | | 1839.2199 | 7 | 12868.31 | 2530 | 16.8-17.8 |
| | | | | | 1366.2435 | 3 | 4097.35 | 2498 | 16.8-17.8 |
| | | | | | 1804.0538 | 7 | 12624.86 | 2543 | 16.8-17.8 |
| | | | | | 1954.5558 | 7 | 13674.72 | 28866 | 16.8-17.8 |
| | | | | | 2735.573 | 5 | 13673.12 | 15745 | 16.8-17.8 |
| | | | | | 1952.1276 | 7 | 13659.57 | 210712 | 17.9-19.8 |
| | | | | | 1888.9499 | 7 | 13217.19 | 40447 | 17.9-19.8 |
| | | | | | 1923.3958 | 7 | 12459.42 | 37351 | 17.9-19.8 |
| | | | | | 1907.3839 | 7 | 13346.48 | 32220 | 17.9-19.8 |
| | | | | | 1867.9408 | 7 | 13070.10 | 31847 | 17.9-19.8 |
| | | | | | 1949.9831 | 6 | 11695.36 | 18389 | 17.9-19.8 |
| | | | | | 1806.2006 | 7 | 12637.61 | 10966 | 17.9-19.8 |
| | | | | | 2273.733 | 6 | 13638.28 | 9398 | 17.9-19.8 |
| | | | | | 1840.9326 | 7 | 12881.82 | 10768 | 17.9-19.8 |
| | | | | | 1904.8103 | 7 | 13332.96 | 10085 | 17.9-19.8 |
| | | | | | 2736.3724 | 5 | 13678.40 | 9423 | 17.9-19.8 |
| | | | | | 1822.069 | 7 | 12750.53 | 8293 | 17.9-19.8 |
| | | | | | 2739.9651 | 5 | 13698.74 | 7362 | 17.9-19.8 |
| | | | | | 1789.904 | 7 | 12534.39 | 6864 | 17.9-19.8 |
| | | | | | 1859.6518 | 7 | 13013.46 | 6635 | 17.9-19.8 |
| | | | | | 3035.9723 | 9 | 27321.78 | 5390 | 17.9-19.8 |
| | | | | | 2743.367 | 5 | 13714.86 | 4678 | 17.9-19.8 |
| | | | | | 1697.2291 | 8 | 13574.03 | 3661 | 17.9-19.8 |
| | | | | | 1771.6151 | 7 | 12395.07 | 2398 | 17.9-19.8 |

*Table S5: A list of all deconvoluted m/z values from the MS data from the SEC separation using 10% ACN + 0.1% FA as the mobile phase. The delay of 1.2 min between the HPLC and MS systems is processed in the retention time (RT).*

| **MS analysis of separation by 10% ACN + 0.1% FA** | | | | | | | | | |
|---|---|---|---|---|---|---|---|---|---|
| *Bungarus multicinctus* | | | | | *Daboia russelii* | | | | |
| *m/z* | Charge | Mass | Intensity | RT | *m/z* | Charge | Mass | Intensity | RT |
| 1724.9243 | 12 | 20692.89 | 44946 | 10.6 - 12.4 | 3616.0636 | 25 | 90359.88 | 1069 | 8.8-11.2 |
| 1890.0054 | 11 | 20778.12 | 31727 | 10.6 - 12.4 | 2200.4308 | 13 | 28592.51 | 2937 | 8.8-11.2 |
| 1886.6445 | 11 | 20739.94 | 36262 | 10.6 - 12.4 | 2211.6683 | 13 | 28738.59 | 2691 | 8.8-11.2 |
| 1876.9987 | 11 | 20641.38 | 16321 | 10.6 - 12.4 | 2267.0054 | 6 | 13597.75 | 242421 | 11.4-14.6 |
| 1726.176 | 12 | 20701.98 | 29614 | 10.6 - 12.4 | 1937.8281 | 6 | 11623.37 | 148514 | 11.4-14.6 |
| 1720.6681 | 12 | 20635.93 | 20467 | 10.6 - 12.4 | 1867.8105 | 7 | 13069.9 | 89962 | 11.4-14.6 |
| 1730.4241 | 12 | 20753.00 | 41991 | 10.6 - 12.4 | 1476.7549 | 9 | 13283.96 | 9596 | 11.4-14.6 |
| 1745.9342 | 12 | 20939.12 | 9310 | 10.6 - 12.4 | 1888.9642 | 7 | 13217.81 | 79290 | 11.4-14.6 |
| 1926.1181 | 7 | 13477.62 | 7099 | 10.6 - 12.4 | 1822.2255 | 7 | 12750.77 | 64814 | 11.4-14.6 |
| 1820.3219 | 4 | 7278.01 | 4796 | 10.6 - 12.4 | 1906.9725 | 7 | 13345.46 | 57179 | 11.4-14.6 |
| 1748.1003 | 12 | 20969.91 | 7445 | 10.6 - 12.4 | 1644.9761 | 8 | 13154.76 | 53266 | 11.4-14.6 |
| 1597.6921 | 5 | 7985.20 | 552058 | 12.6 - 16.9 | 1947.8313 | 6 | 11688.66 | 47754 | 11.4-14.6 |
| 1603.2969 | 5 | 8013.53 | 331287 | 12.6 - 16.9 | 1671.7141 | 7 | 11697.34 | 99270 | 11.4-14.6 |
| 1877.0481 | 4 | 7506.61 | 142368 | 12.6 - 16.9 | 1909.3096 | 6 | 11451.44 | 40424 | 11.4-14.6 |
| 1831.9035 | 7 | 12819.56 | 105834 | 12.6 - 16.9 | 1934.1548 | 6 | 11608.25 | 34084 | 11.4-14.6 |
| 1815.7433 | 5 | 9076.14 | 57326 | 12.6 - 16.9 | 1683.1076 | 8 | 13459.78 | 36644 | 11.4-14.6 |
| 1505.6379 | 5 | 7524.74 | 49234 | 12.6 - 16.9 | 1804.0717 | 7 | 12630.49 | 36898 | 11.4-14.6 |
| 1472.6258 | 5 | 7358.46 | 41910 | 12.6 - 16.9 | 1797.7833 | 7 | 12579.22 | 35370 | 11.4-14.6 |
| 1600.6827 | 5 | 8000.34 | 40285 | 12.6 - 16.9 | 1781.3435 | 7 | 12465.41 | 34300 | 11.4-14.6 |
| 1454.0115 | 5 | 7267.31 | 37139 | 12.6 - 16.9 | 1921.8232 | 6 | 11527.05 | 33600 | 11.4-14.6 |
| 1812.7417 | 5 | 9058.70 | 36682 | 12.6 - 16.9 | 2279.827 | 6 | 13674.56 | 30907 | 11.4-14.6 |
| 1834.3302 | 7 | 12834.26 | 33583 | 12.6 - 16.9 | 1934.1548 | 6 | 11600.65 | 34084 | 11.4-14.6 |
| 1799.4566 | 7 | 12591.65 | 32700 | 12.6 - 16.9 | 1474.758 | 9 | 13267.29 | 6984 | 11.4-14.6 |
| 1328.5711 | 6 | 7967.56 | 30352 | 12.6 - 16.9 | 1771.4818 | 7 | 12395.34 | 26785 | 11.4-14.6 |
| 1606.6904 | 5 | 8030.27 | 30112 | 12.6 - 16.9 | 1787.7729 | 7 | 12512.57 | 26853 | 11.4-14.6 |
| 1609.6881 | 5 | 8045.69 | 24347 | 12.6 - 16.9 | 1904.9689 | 7 | 13331.35 | 23262 | 11.4-14.6 |
| 1819.3381 | 5 | 9092.45 | 22277 | 12.6 - 16.9 | 1487.4342 | 9 | 13388.60 | 5687 | 11.4-14.6 |
| 1804.7227 | 5 | 9019.86 | 21688 | 12.6 - 16.9 | 1675.4914 | 8 | 13397.78 | 187178 | 11.4-14.6 |
| 1508.6358 | 5 | 7539.39 | 19723 | 12.6 - 16.9 | 1968.677 | 6 | 11806.23 | 20734 | 11.4-14.6 |
| 2250.8969 | 4 | 9002.24 | 7401 | 12.6 - 16.9 | 1978.3442 | 6 | 11866.06 | 19978 | 11.4-14.6 |
| 1461.6123 | 5 | 7305.08 | 18214 | 12.6 - 16.9 | 1882.1326 | 6 | 11290.30 | 32331 | 11.4-14.6 |
| 1326.5723 | 6 | 7955.35 | 15317 | 12.6 - 16.9 | 1997.1928 | 6 | 11979.34 | 18808 | 11.4-14.6 |
| 1498.6301 | 5 | 7490.40 | 15306 | 12.6 - 16.9 | 1762.9083 | 7 | 12336.14 | 17815 | 11.4-14.6 |
| 1475.6121 | 5 | 7376.84 | 15242 | 12.6 - 16.9 | 1820.0836 | 7 | 12741.02 | 20416 | 11.4-14.6 |
| | | | | | 1689.4974 | 8 | 13510.89 | 16963 | 11.4-14.6 |
| | | | | | 1838.9462 | 7 | 12872.08 | 21344 | 11.4-14.6 |
| | | | | | 1987.5243 | 6 | 11920.52 | 15473 | 11.4-14.6 |
| | | | | | 1886.9642 | 7 | 13207.28 | 21836 | 11.4-14.6 |
| | | | | | 2283.3266 | 6 | 13696.69 | 11155 | 11.4-14.6 |
| | | | | | 1931.4869 | 6 | 11584.95 | 14385 | 11.4-14.6 |
| | | | | | 1634.8392 | 7 | 11441.06 | 14123 | 11.4-14.6 |
| | | | | | 1865.9566 | 7 | 13058.45 | 31419 | 11.4-14.6 |
| | | | | | 1509.6674 | 9 | 13588.78 | 13739 | 11.4-14.6 |
| | | | | | 1813.9362 | 7 | 12692.86 | 13677 | 11.4-14.6 |
| | | | | | 1832.7988 | 7 | 12824.33 | 13214 | 11.4-14.6 |
| | | | | | 1753.0476 | 7 | 12265.77 | 12624 | 11.4-14.6 |
| | | | | | 1681.2322 | 8 | 13447.08 | 13960 | 11.4-14.6 |
| | | | | | 1769.1957 | 7 | 12383.31 | 10809 | 11.4-14.6 |
| | | | | | 1744.6156 | 7 | 12206.86 | 10789 | 11.4-14.6 |
| | | | | | 2028.2127 | 6 | 12164.73 | 10039 | 11.4-14.6 |
| | | | | | 1949.0735 | 7 | 13637.83 | 38551 | 14.8-16.2 |
| | | | | | 1916.4848 | 7 | 13411.10 | 12864 | 14.8-16.2 |
| | | | | | 1877.3241 | 7 | 13135.64 | 9422 | 14.8-16.2 |
| | | | | | 1895.6175 | 7 | 13263.71 | 6157 | 14.8-16.2 |
| | | | | | 1656.538 | 8 | 13246.01 | 4896 | 14.8-16.2 |
| | | | | | 1856.1689 | 7 | 12988.34 | 4565 | 14.8-16.2 |
| | | | | | 1543.3413 | 9 | 13883.42 | 19743 | 21.1-23.4 |
| | | | | | 1672.4221 | 7 | 11703.31 | 2184 | 21.1-23.4 |
| | | | | | 1698.8611 | 7 | 11887.53 | 1806 | 21.1-23.4 |
| | | | | | 1689.4857 | 8 | 13508.40 | 1264 | 21.1-23.4 |
| | | | | | 1531.7797 | 9 | 13778.90 | 1240 | 21.1-23.4 |

*Table 6: A list of all deconvoluted m/z values from the MS data from the SEC separation using 10% ACN + 0.05% FA as the mobile phase. The delay of 1.2 min between the HPLC and MS systems is processed in the retention time (RT).*

| **MS analysis of separation by 10% ACN + 0.05% FA** | | | | | | | | | |
|---|---|---|---|---|---|---|---|---|---|
| *Bungarus multicinctus* | | | | | *Daboia russelii* | | | | |
| *m/z* | Charge | Mass | Intensity | RT | *m/z* | Charge | Mass | Intensity | RT |
| 1544.2289 | 9 | 13882.09 | 5103 | 1.0 - 8.4 | 2383.4758 | 13 | 30972.09 | 3884 | 9.3-11.3 |
| 1699.0003 | 7 | 11886.95 | 913 | 1.0 - 8.4 | 2359.3129 | 13 | 30657.96 | 2294 | 9.3-11.3 |
| 1707.6169 | 8 | 13652.88 | 2023 | 1.0 - 8.4 | 1926.0457 | 13 | 25019.41 | 5068 | 9.3-11.3 |
| 1724.9206 | 12 | 20686.96 | 32912 | 8.6 - 12.2 | 1784.2534 | 14 | 24949.51 | 3243 | 9.3-11.3 |
| 1729.3396 | 12 | 20726.90 | 31875 | 8.6 - 12.2 | 3477.0974 | 26 | 90378.34 | 2029 | 9.3-11.3 |
| 1592.2346 | 12 | 19082.67 | 22717 | 8.6 - 12.2 | 2266.8402 | 6 | 13585.95 | 248619 | 11.3-13 |
| 1732.4201 | 12 | 20776.95 | 27566 | 8.6 - 12.2 | 1675.4924 | 8 | 13386.84 | 180504 | 11.3-13 |
| 1740.5956 | 12 | 20875.06 | 10856 | 8.6 - 12.2 | 1937.8294 | 6 | 11612.90 | 176089 | 11.3-13 |
| 1720.582 | 12 | 20634.90 | 14787 | 8.6 - 12.2 | 1644.9787 | 6 | 13144.74 | 56234 | 11.3-13 |
| 1456.4546 | 5 | 7277.24 | 6715 | 8.6 - 12.2 | 1658.8541 | 8 | 13260.74 | 42097 | 11.3-13 |
| 1726.0032 | 12 | 20696.87 | 21123 | 8.6 - 12.2 | 1921.6538 | 6 | 11515.84 | 39255 | 11.3-13 |
| 1597.6872 | 5 | 7978.38 | 482007 | 12.4 - 15.9 | 1781.4879 | 7 | 12456.34 | 36925 | 11.3-13 |
| 1603.292 | 5 | 8005.39 | 264255 | 12.4 - 15.9 | 1797.6428 | 7 | 12569.42 | 35263 | 11.3-13 |
| 1877.2931 | 4 | 7499.12 | 131493 | 12.4 - 15.9 | 1934.9907 | 6 | 11598.88 | 26962 | 11.3-13 |
| 1832.0409 | 7 | 12808.20 | 95572 | 12.4 - 15.9 | 1968.6775 | 6 | 11799.99 | 21403 | 11.3-13 |
| 1815.7375 | 5 | 9066.61 | 52441 | 12.4 - 15.9 | 1907.319 | 6 | 11431.84 | 20113 | 11.3-13 |
| 1505.633 | 5 | 7517.11 | 40901 | 12.4 - 15.9 | 1762.91 | 7 | 12328.29 | 18073 | 11.3-13 |
| 1472.6201 | 5 | 1352.05 | 35018 | 12.4 - 15.9 | 1673.1374 | 8 | 13368.80 | 17918 | 11.3-13 |
| 1812.3335 | 5 | 9049.60 | 34012 | 12.4 - 15.9 | 1987.5278 | 6 | 11912.08 | 17225 | 11.3-13 |
| 1600.6774 | 5 | 7992.36 | 32324 | 12.4 - 15.9 | 1626.8444 | 8 | 12997.67 | 17053 | 11.3-13 |
| 1799.4518 | 7 | 12581.08 | 29672 | 12.4 - 15.9 | 1879.9718 | 6 | 11269.77 | 34503 | 11.3-13 |
| 1328.5665 | 6 | 7959.34 | 24706 | 12.4 - 15.9 | 1689.5003 | 8 | 13500.91 | 14225 | 11.3-13 |
| 1606.6853 | 5 | 8022.37 | 24080 | 12.4 - 15.9 | 2269.6692 | 6 | 13603.94 | 15362 | 11.3-13 |
| 1834.1824 | 7 | 12826.20 | 28376 | 12.4 - 15.9 | 1509.7811 | 9 | 13568.91 | 15387 | 11.3-13 |
| 1818.7361 | 5 | 9083.62 | 22179 | 12.4 - 15.9 | 1832.6586 | 7 | 12815.54 | 12486 | 11.3-13 |
| 1609.6836 | 5 | 8037.36 | 21395 | 12.4 - 15.9 | 1744.6172 | 7 | 12199.25 | 12145 | 11.3-13 |
| 1804.716 | 5 | 9012.52 | 19695 | 12.4 - 15.9 | 1642.8479 | 8 | 13125.69 | 12031 | 11.3-13 |
| 1454.1993 | 5 | 7259.92 | 18345 | 12.4 - 15.9 | 3022.2265 | 9 | 27187.92 | 11940 | 11.3-13 |
| 1461.6061 | 5 | 7304.45 | 15318 | 12.4 - 15.9 | 1865.4659 | 6 | 11180.73 | 11259 | 11.3-13 |
| 1498.6257 | 5 | 7489.37 | 13846 | 12.4 - 15.9 | 1918.814 | 6 | 11498.82 | 10113 | 11.3-13 |
| 1822.7507 | 4 | 7287.04 | 13513 | 12.4 - 15.9 | 2277.3278 | 6 | 13648.87 | 271355 | 13.1-15.1 |
| 1807.7162 | 5 | 9035.88 | 12415 | 12.4 - 15.9 | 1949.9954 | 6 | 11687.90 | 227528 | 13.1-15.1 |
| 1476.6116 | 5 | 7377.41 | 11526 | 12.4 - 15.9 | 1806.0702 | 7 | 12628.41 | 166219 | 13.1-15.1 |
| 1326.5671 | 6 | 7954.37 | 11518 | 12.4 - 15.9 | 1867.9539 | 7 | 13059.57 | 126591 | 13.1-15.1 |
| | | | | | 1888.8186 | 7 | 13206.64 | 110394 | 13.1-15.1 |
| | | | | | 1789.9156 | 7 | 12514.32 | 106364 | 13.1-15.1 |
| | | | | | 1822.2238 | 7 | 12741.48 | 94585 | 13.1-15.1 |
| | | | | | 1840.9438 | 7 | 12872.52 | 93268 | 13.1-15.1 |
| | | | | | 1909.3092 | 6 | 11444.78 | 65099 | 13.1-15.1 |
| | | | | | 1907.2523 | 7 | 13337.69 | 59208 | 13.1-15.1 |
| | | | | | 1933.987 | 6 | 11589.85 | 52979 | 13.1-15.1 |
| | | | | | 1683.1056 | 8 | 13450.77 | 51544 | 13.1-15.1 |
| | | | | | 1882.132 | 6 | 11278.71 | 48848 | 13.1-15.1 |
| | | | | | 1771.4796 | 7 | 12385.28 | 42055 | 13.1-15.1 |
| | | | | | 1859.8049 | 7 | 13003.54 | 32577 | 13.1-15.1 |
| | | | | | 1978.5096 | 6 | 11857.98 | 30611 | 13.1-15.1 |
| | | | | | 1946.9892 | 6 | 11667.85 | 29751 | 13.1-15.1 |
| | | | | | 1997.3572 | 6 | 11971.07 | 28346 | 13.1-15.1 |
| | | | | | 1787.3402 | 7 | 12496.29 | 24461 | 13.1-15.1 |
| | | | | | 1904.6789 | 7 | 13318.62 | 22184 | 13.1-15.1 |
| | | | | | 1966.5041 | 6 | 11786.95 | 20531 | 13.1-15.1 |
| | | | | | 1752.9009 | 7 | 12257.24 | 18522 | 13.1-15.1 |
| | | | | | 1803.35 | 7 | 12608.37 | 17531 | 13.1-15.1 |
| | | | | | 2028.209 | 6 | 12157.19 | 14871 | 13.1-15.1 |
| | | | | | 1930.9763 | 6 | 11571.80 | 10894 | 13.1-15.1 |
| | | | | | 1768.7608 | 7 | 12366.24 | 10862 | 13.1-15.1 |
| | | | | | 1948.9279 | 7 | 13627.41 | 60796 | 15.2-16.6 |
| | | | | | 1916.6246 | 7 | 13400.28 | 32763 | 15.2-16.6 |
| | | | | | 1877.1779 | 7 | 13124.16 | 24573 | 15.2-16.6 |
| | | | | | 1895.4718 | 7 | 13252.21 | 16466 | 15.2-16.6 |
| | | | | | 1856.1688 | 7 | 12977.09 | 13483 | 15.2-16.6 |
| | | | | | 1656.4099 | 8 | 13236.19 | 12904 | 15.2-16.6 |
| | | | | | 1703.4331 | 8 | 13609.37 | 7282 | 15.2-16.6 |

*Table S7: A list of all deconvoluted m/z values from the MS data from the SEC separation using 10% ACN + 0.1% DFA as the mobile phase. The delay of 1.2 min between the HPLC and MS systems is processed in the retention time (RT).*

| **MS analysis of separation by 10% ACN + 0.1% DFA** | | | | | | | | | |
|---|---|---|---|---|---|---|---|---|---|
| *Bungarus multicinctus* | | | | | *Daboia russelii* | | | | |
| *m/z* | Charge | Mass | Intensity | RT | *m/z* | Charge | Mass | Intensity | RT |
| 2076.1062 | 10 | 20754.35 | 31737 | 13.1 - 14.9 | 2246.8255 | 11 | 24707.53 | 12286 | 12.8-14.2 |
| 2069.7038 | 10 | 20692.41 | 31687 | 13.1 - 14.9 | 2086.2859 | 12 | 25028.50 | 7696 | 12.8-14.2 |
| 2078.8038 | 10 | 20781.59 | 25006 | 13.1 - 14.9 | 2081.3689 | 12 | 24971.60 | 5007 | 12.8-14.2 |
| 2073.8006 | 10 | 20736.72 | 18832 | 13.1 - 14.9 | 2383.467 | 12 | 28595.25 | 4791 | 12.8-14.2 |
| 2064.3973 | 10 | 20639.60 | 12394 | 13.1 - 14.9 | 2395.8855 | 12 | 28742.11 | 4708 | 12.8-14.2 |
| 1898.6496 | 11 | 20878.93 | 7814 | 13.1 - 14.9 | 1700.5077 | 8 | 13597.65 | 608257 | 14.3-15.9 |
| 1904.6531 | 11 | 20933.37 | 7724 | 13.1 - 14.9 | 1675.3674 | 6 | 13397.60 | 107574 | 14.3-15.9 |
| 1906.9291 | 11 | 20962.19 | 6300 | 13.1 - 14.9 | 1937.9971 | 6 | 11623.59 | 95271 | 14.3-15.9 |
| 1775.2185 | 9 | 15972.18 | 6023 | 13.1 - 14.9 | 1476.7566 | 9 | 13284.17 | 7456 | 14.3-15.9 |
| 1778.2206 | 9 | 15998.96 | 5808 | 13.1 - 14.9 | 1698.504 | 8 | 13591.07 | 35761 | 14.3-15.9 |
| 1331.5787 | 6 | 7985.08 | 661916 | 15.1 - 19.0 | 1702.3811 | 8 | 13612.71 | 33649 | 14.3-15.9 |
| 1336.2486 | 6 | 8013.45 | 297208 | 15.1 - 19.0 | 1644.9761 | 8 | 13154.79 | 28960 | 14.3-15.9 |
| 1328.5747 | 6 | 7967.57 | 92845 | 15.1 - 19.0 | 1934.9944 | 6 | 11605.54 | 16782 | 14.3-15.9 |
| 1502.04 | 5 | 7506.73 | 67672 | 15.1 - 19.0 | 1879.9718 | 6 | 11275.98 | 22571 | 14.3-15.9 |
| 1334.2428 | 6 | 8004.70 | 60782 | 15.1 - 19.0 | 1921.6519 | 6 | 11525.91 | 19557 | 14.3-15.9 |
| 1831.9018 | 7 | 12819.86 | 59691 | 15.1 - 19.0 | 1781.488 | 7 | 12465.39 | 18580 | 14.3-15.9 |
| 1338.9125 | 6 | 8030.06 | 35147 | 15.1 - 19.0 | 1797.5427 | 7 | 12578.61 | 18480 | 14.3-15.9 |
| 1513.4491 | 6 | 9076.08 | 32849 | 15.1 - 19.0 | 1707.2487 | 8 | 13650.10 | 14803 | 14.3-15.9 |
| 1461.4169 | 5 | 7304.38 | 31181 | 15.1 - 19.0 | 1673.2392 | 8 | 13387.22 | 12538 | 14.3-15.9 |
| 1472.427 | 5 | 7358.86 | 30190 | 15.1 - 19.0 | 1689.6264 | 8 | 13511.86 | 11359 | 14.3-15.9 |
| 1505.6386 | 6 | 7524.89 | 30455 | 15.1 - 19.0 | 1763.0537 | 7 | 12335.88 | 10082 | 14.3-15.9 |
| 1817.2681 | 4 | 7267.69 | 29655 | 15.1 - 19.0 | 1907.3175 | 6 | 11439.21 | 9673 | 14.3-15.9 |
| 1326.5766 | 6 | 7957.04 | 29360 | 15.1 - 19.0 | 1987.529 | 6 | 11920.75 | 9634 | 14.3-15.9 |
| 1510.4478 | 6 | 9058.15 | 25042 | 15.1 - 19.0 | 1952.1352 | 7 | 13659.66 | 212225 | 15.9-17.7 |
| 1341.5753 | 6 | 8045.26 | 24831 | 15.1 - 19.0 | 1888.9586 | 7 | 13217.30 | 93556 | 15.9-17.7 |
| 1834.3295 | 7 | 12834.79 | 24495 | 15.1 - 19.0 | 1867.8056 | 7 | 13070.06 | 78397 | 15.9-17.7 |
| 1475.6254 | 5 | 7374.87 | 23844 | 15.1 - 19.0 | 1907.2486 | 7 | 13346.52 | 71325 | 15.9-17.7 |
| 1324.0736 | 6 | 7941.49 | 23702 | 15.1 - 19.0 | 1923.4028 | 7 | 13459.52 | 70786 | 15.9-17.7 |
| 1455.6152 | 5 | 7276.08 | 24232 | 15.1 - 19.0 | 1949.8228 | 6 | 11695.96 | 39561 | 15.9-17.7 |
| 1312.5637 | 6 | 7872.25 | 16537 | 15.1 - 19.0 | 1806.0657 | 7 | 12637.59 | 33803 | 15.9-17.7 |
| 1516.1177 | 6 | 9092.61 | 16359 | 15.1 - 19.0 | 1841.082 | 7 | 12882.07 | 27846 | 15.9-17.7 |
| 1466.6217 | 5 | 7331.18 | 14858 | 15.1 - 19.0 | 1822.2192 | 7 | 12750.80 | 23856 | 15.9-17.7 |
| 1315.067 | 6 | 78867.00 | 14535 | 15.1 - 19.0 | 1904.6741 | 7 | 13327.37 | 21600 | 15.9-17.7 |
| 1321.5708 | 6 | 7926.60 | 14101 | 15.1 - 19.0 | 1790.0525 | 7 | 12524.59 | 20106 | 15.9-17.7 |
| 1458.6125 | 5 | 7290.88 | 12372 | 15.1 - 19.0 | 1859.8013 | 7 | 13013.97 | 15478 | 15.9-17.7 |
| 1317.5667 | 6 | 7901.60 | 12301 | 15.1 - 19.0 | 1380.7899 | 5 | 6901.10 | 10013 | 15.9-17.7 |
| 1509.2398 | 5 | 7541.91 | 12373 | 15.1 - 19.0 | | | | | |
| 1310.0637 | 6 | 7856.86 | 11877 | 15.1 - 19.0 | | | | | |
| 1319.5696 | 6 | 7614.60 | 11740 | 15.1 - 19.0 | | | | | |
| 1767.7402 | 4 | 7069.46 | 9246 | 15.1 - 19.0 | | | | | |

*Table S8: A list of all deconvoluted m/z values from the MS data from the SEC separation using 10% ACN + 0.05% DFA as the mobile phase. The delay of 1.2 min between the HPLC and MS systems is processed in the retention time (RT).*

| **MS analysis of separation by 10% ACN + 0.05% DFA** | | | | | | | | | |
|---|---|---|---|---|---|---|---|---|---|
| *Bungarus multicinctus* | | | | | *Daboia russelii* | | | | |
| *m/z* | Charge | Mass | Intensity | RT | *m/z* | Charge | Mass | Intensity | RT |
| 2076.1957 | 10 | 20742.95 | 32771 | 12.2 - 14.0 | 2246.7402 | 11 | 24707.61 | 19572 | 11.6-13.1 |
| 1889.9029 | 11 | 20771.36 | 26195 | 12.2 - 14.0 | 2086.3749 | 12 | 25028.51 | 9479 | 11.6-13.1 |
| 2073.7902 | 10 | 20731.17 | 20644 | 12.2 - 14.0 | 2395.7254 | 12 | 28741.21 | 6644 | 11.6-13.1 |
| 1775.2079 | 9 | 15972.30 | 9832 | 12.2 - 14.0 | 2081.2889 | 12 | 24968.83 | 5352 | 11.6-13.1 |
| 1778.4337 | 9 | 15999.66 | 9285 | 12.2 - 14.0 | 1700.5064 | 8 | 13597.64 | 500958 | 13.2-17.1 |
| 1881.631 | 11 | 20678.79 | 34658 | 12.2 - 14.0 | 1952.1393 | 7 | 13660.12 | 175840 | 13.2-17.1 |
| 2071.0899 | 10 | 20699.51 | 22965 | 12.2 - 14.0 | 1675.4915 | 8 | 13397.71 | 88561 | 13.2-17.1 |
| 2064.4867 | 10 | 20630.37 | 14394 | 12.2 - 14.0 | 1888.8194 | 7 | 13217.29 | 80415 | 13.2-17.1 |
| 1331.5703 | 6 | 7985.01 | 778230 | 14.1 - 17.5 | 1867.8097 | 7 | 13070.09 | 66804 | 13.2-17.1 |
| 1336.2402 | 6 | 8013.36 | 333137 | 14.1 - 17.5 | 1907.8965 | 7 | 13347.26 | 62611 | 13.2-17.1 |
| 1328.5662 | 6 | 7967.54 | 108420 | 14.1 - 17.5 | 1923.5511 | 7 | 13460.06 | 61103 | 13.2-17.1 |
| 1501.8299 | 5 | 7506.54 | 71957 | 14.1 - 17.5 | 1937.9947 | 6 | 11623.83 | 59269 | 13.2-17.1 |
| 1832.033 | 7 | 12820.15 | 68372 | 14.1 - 17.5 | 1660.9881 | 7 | 13284.34 | 51333 | 13.2-17.1 |
| 1334.2348 | 6 | 8004.30 | 66483 | 14.1 - 17.5 | 1954.4234 | 7 | 13675.37 | 35276 | 13.2-17.1 |
| 1817.5076 | 4 | 7268.18 | 43257 | 14.1 - 17.5 | 1671.7132 | 7 | 11696.93 | 21945 | 13.2-17.1 |
| 1513.4401 | 6 | 9076.04 | 40321 | 14.1 - 17.5 | 1698.376 | 8 | 13590.71 | 29872 | 13.2-17.1 |
| 1461.4076 | 5 | 7304.15 | 36285 | 14.1 - 17.5 | 1702.5029 | 8 | 13613.52 | 25194 | 13.2-17.1 |
| 1338.9041 | 6 | 8029.64 | 36028 | 14.1 - 17.5 | 1645.2247 | 8 | 13155.52 | 20725 | 13.2-17.1 |
| 1326.5685 | 6 | 7956.94 | 32951 | 14.1 - 17.5 | 1789.9148 | 7 | 12524.46 | 18784 | 13.2-17.1 |
| 1505.6286 | 5 | 7524.81 | 32427 | 14.1 - 17.5 | 1859.6641 | 7 | 13012.34 | 15576 | 13.2-17.1 |
| 1510.4384 | 6 | 9058.21 | 31460 | 14.1 - 17.5 | 1891.1043 | 7 | 13233.13 | 14491 | 13.2-17.1 |
| 1472.6171 | 5 | 7359.27 | 27850 | 14.1 - 17.5 | 1797.6405 | 7 | 12578.77 | 13958 | 13.2-17.1 |
| 1324.0655 | 6 | 7941.41 | 27089 | 14.1 - 17.5 | 1658.8521 | 8 | 13269.48 | 13747 | 13.2-17.1 |
| 1341.5674 | 6 | 8045.40 | 27085 | 14.1 - 17.5 | 1781.4861 | 7 | 12465.59 | 13234 | 13.2-17.1 |
| 1834.3175 | 7 | 12834.45 | 26184 | 14.1 - 17.5 | 1956.9981 | 7 | 13695.02 | 12083 | 13.2-17.1 |
| 1475.614 | 5 | 7375.20 | 20280 | 14.1 - 17.5 | 1934.9906 | 7 | 13542.30 | 11626 | 13.2-17.1 |
| 1516.1084 | 6 | 9092.37 | 19015 | 14.1 - 17.5 | 1886.9637 | 7 | 13206.40 | 22833 | 13.2-17.1 |
| 1312.5555 | 6 | 7872.19 | 18656 | 14.1 - 17.5 | 1698.376 | 8 | 13576.21 | 29872 | 13.2-17.1 |
| 1248.8552 | 6 | 7489.84 | 16878 | 14.1 - 17.5 | 1685.2335 | 8 | 13475.73 | 10861 | 13.2-17.1 |
| 1315.2253 | 6 | 7887.50 | 16451 | 14.1 - 17.5 | 1804.0695 | 7 | 12629.33 | 10686 | 13.2-17.1 |
| 1321.7324 | 6 | 7927.27 | 15793 | 14.1 - 17.5 | 1870.0967 | 7 | 13085.40 | 11350 | 13.2-17.1 |
| 1317.5594 | 6 | 7901.46 | 14302 | 14.1 - 17.5 | 1806.0698 | 7 | 12639.61 | 32572 | 13.2-17.1 |
| 1458.6034 | 5 | 7290.97 | 13910 | 14.1 - 17.5 | 1689.4988 | 8 | 13511.50 | 9911 | 13.2-17.1 |
| 1319.5615 | 6 | 7914.61 | 13382 | 14.1 - 17.5 | 2176.9441 | 6 | 13055.74 | 4591 | 13.2-17.1 |
| | | | | | 1838.9457 | 7 | 12874.62 | 8248 | 13.2-17.1 |
| | | | | | 1959.6976 | 7 | 13713.97 | 8122 | 13.2-17.1 |
| | | | | | 1881.9648 | 6 | 11287.91 | 7911 | 13.2-17.1 |
| | | | | | 1694.8729 | 8 | 13556.13 | 7841 | 13.2-17.1 |
| | | | | | 1902.8227 | 7 | 13320.35 | 7490 | 13.2-17.1 |
| | | | | | 1820.3683 | 7 | 12743.59 | 6971 | 13.2-17.1 |
| | | | | | 1771.4798 | 7 | 12884.33 | 6711 | 13.2-17.1 |
| | | | | | 1987.5267 | 6 | 11920.65 | 6693 | 13.2-17.1 |
| | | | | | 1787.7729 | 7 | 12512.23 | 7056 | 13.2-17.1 |

## S7.2. TICs and EICs

Each figure contains the TIC and EICs for one specific venom separation. The upper figure shows the TIC while the figure below shows the corresponding EICs. The delay of 1.2 min between the HPLC system and the MS is not converted in this data. The EICs m/z values are listed below the figure and divided into the RT section that was taken for deconvolution per peak. Figures S16-S21 show data from the *B. multicinctus* venom, while Figures S22-S27 show data for the *D. russelii* venom.

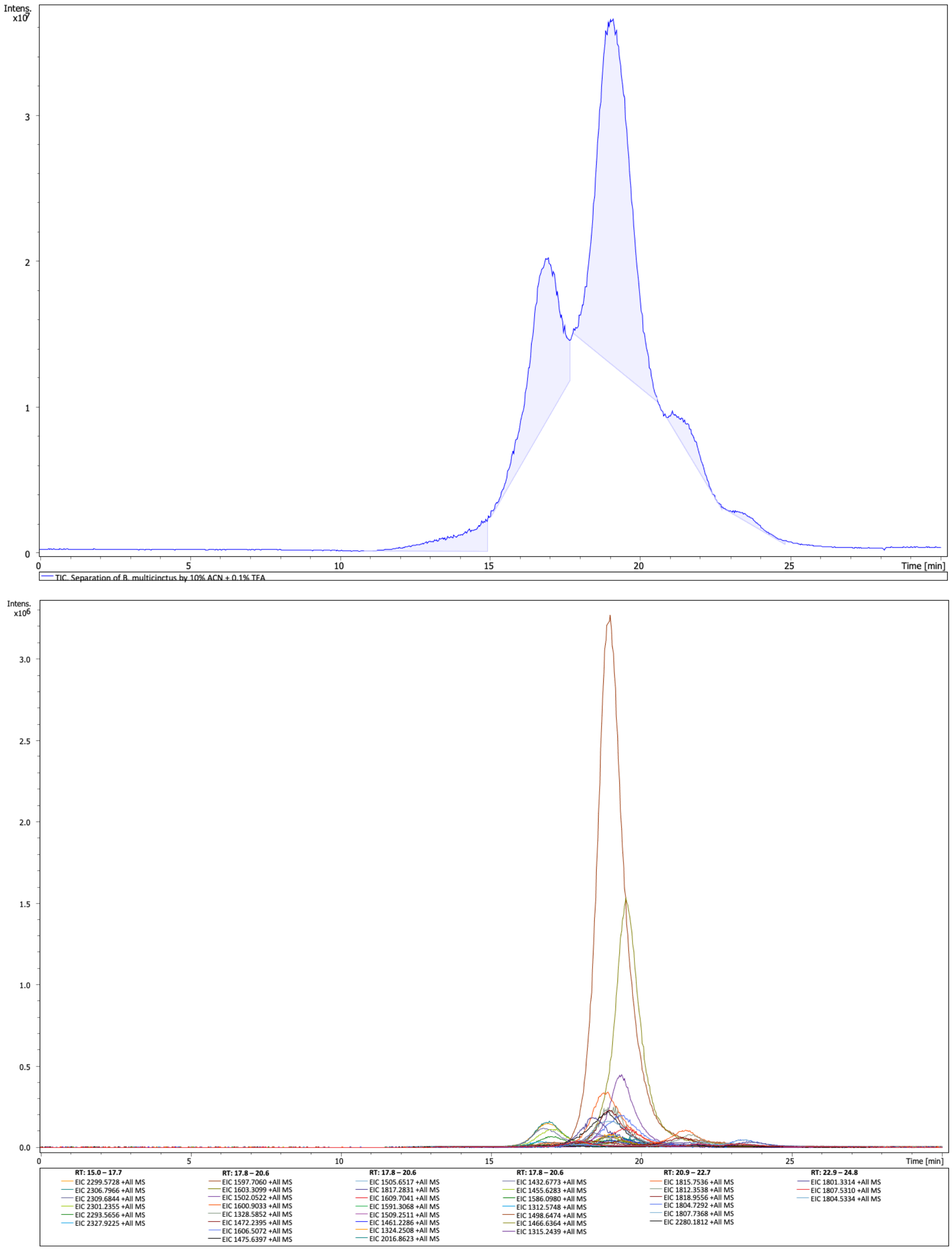


*Figure S16: TIC and EICs of SEC-MS measurement of B. multicinctus venom separated using 10% ACN + 0.1% TFA.*

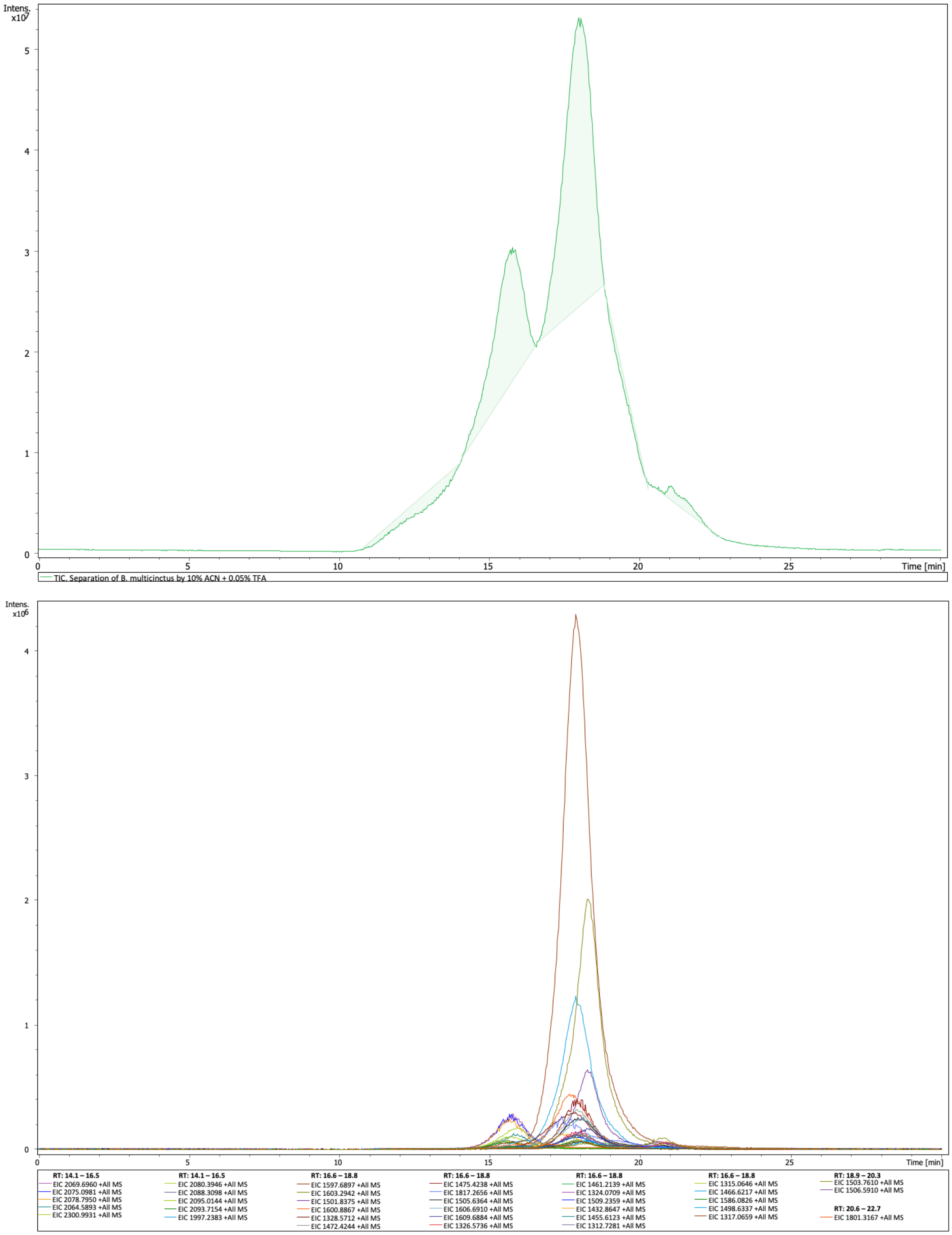


*Figure 17: TIC and EICs of SEC-MS measurement of B. multicinctus venom separated using 10% ACN + 0.05% TFA.*

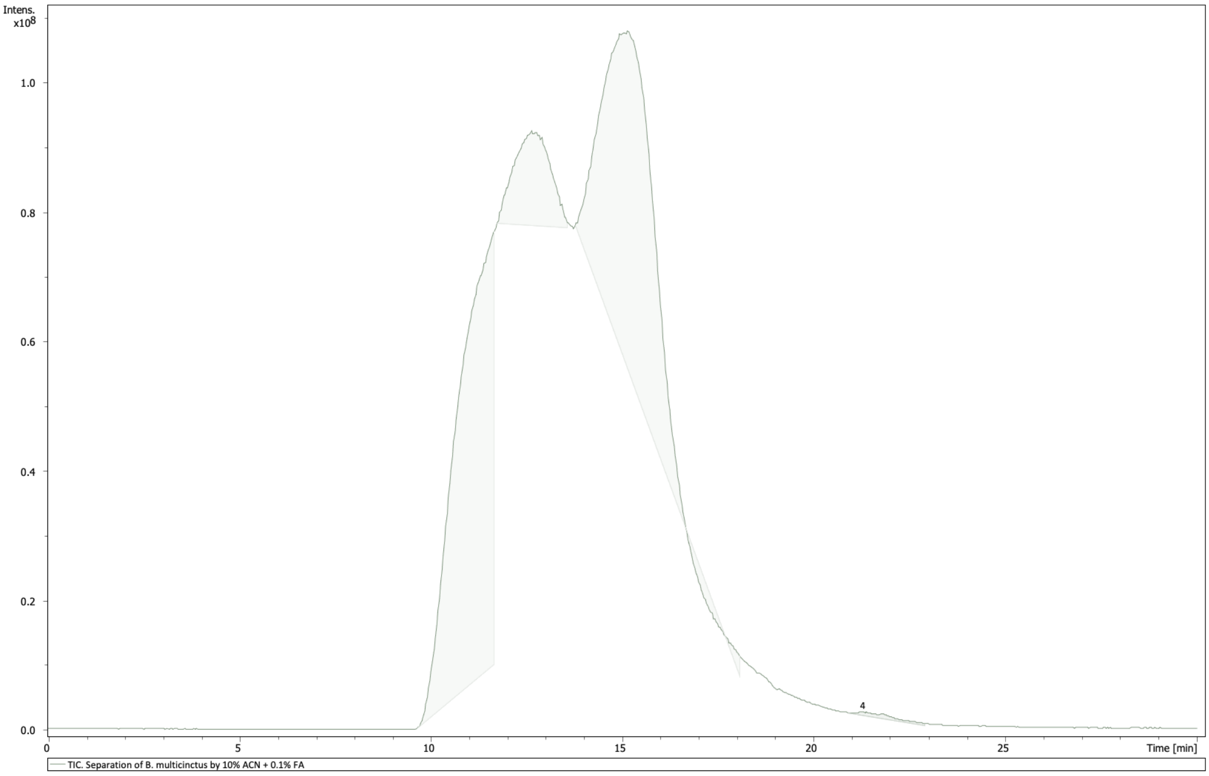


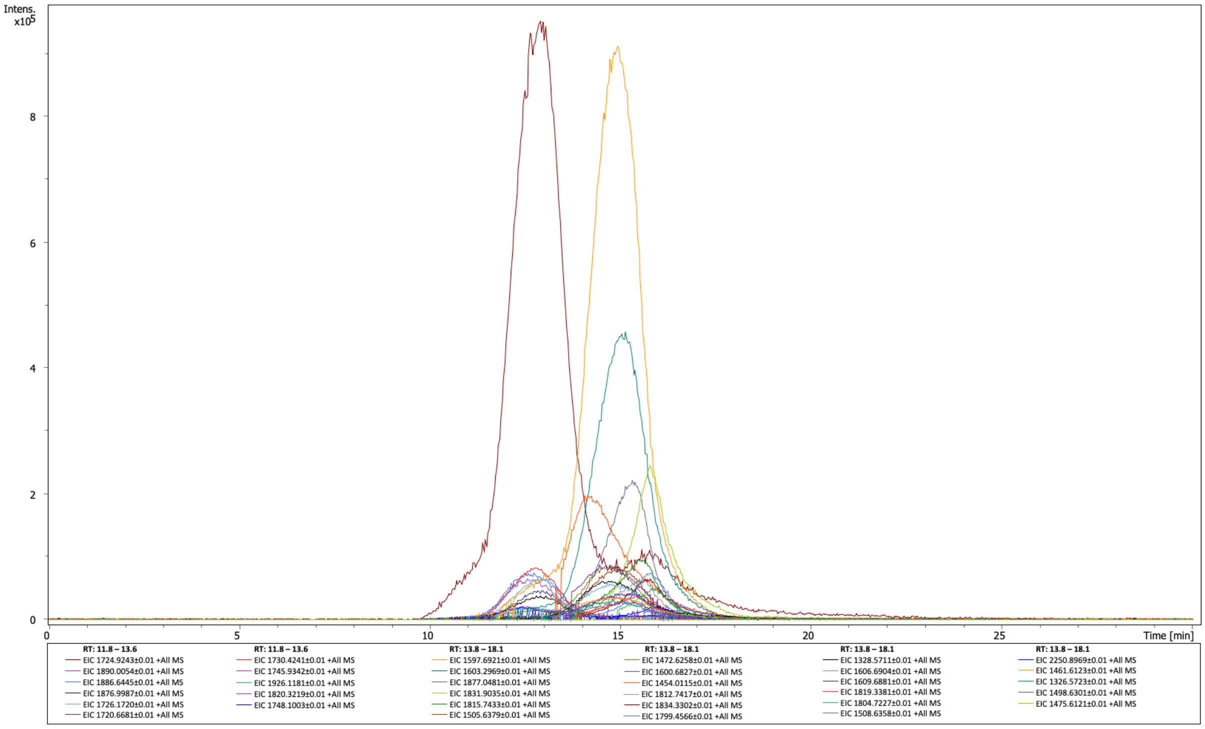


*Figure S18: TIC and EICs of SEC-MS measurement of B. multicinctus venom separated using 10% ACN + 0.1% FA.*

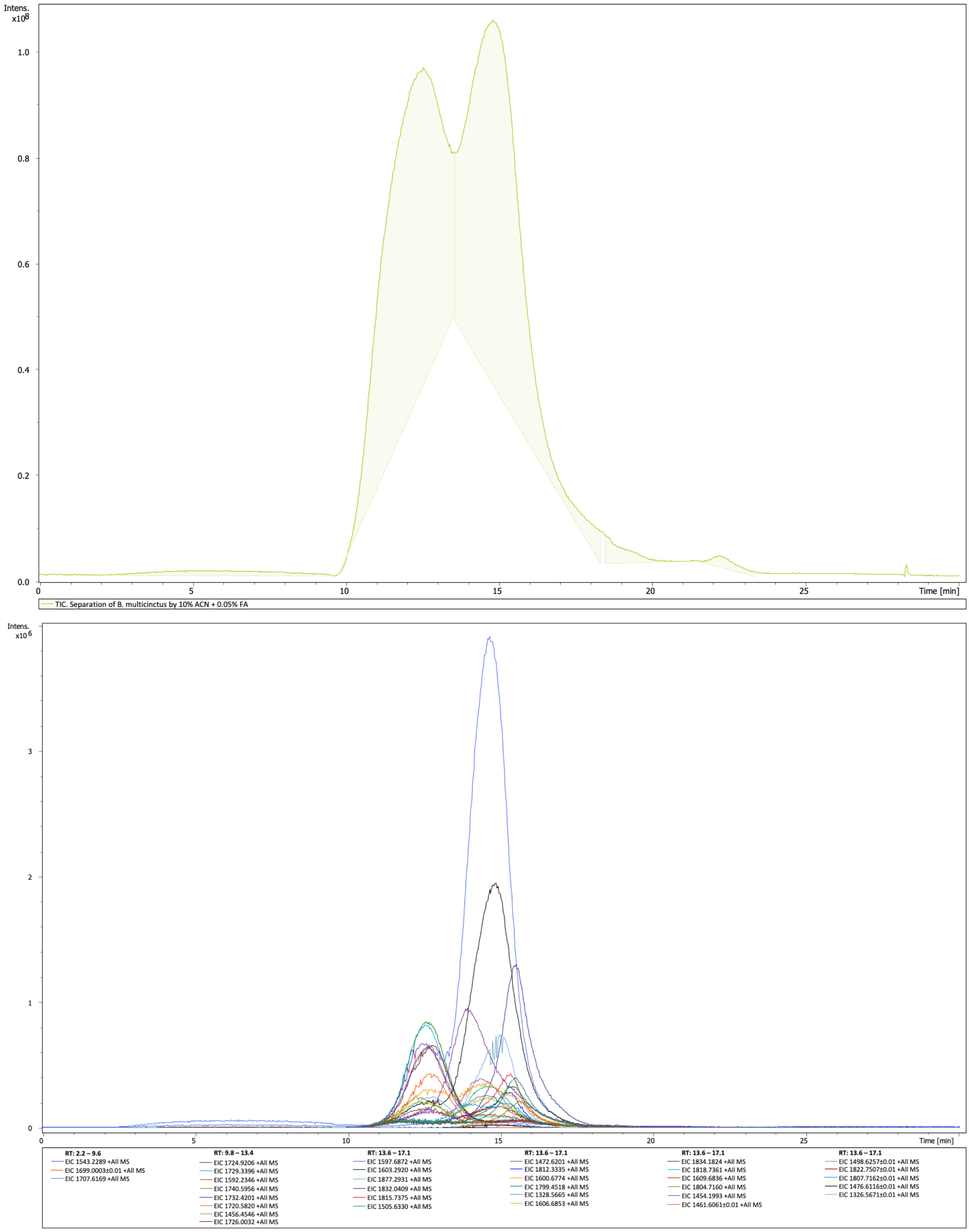


*Figure S19: TIC and EICs of SEC-MS measurement of B. multicinctus venom separated using 10% ACN + 0.05% FA.*

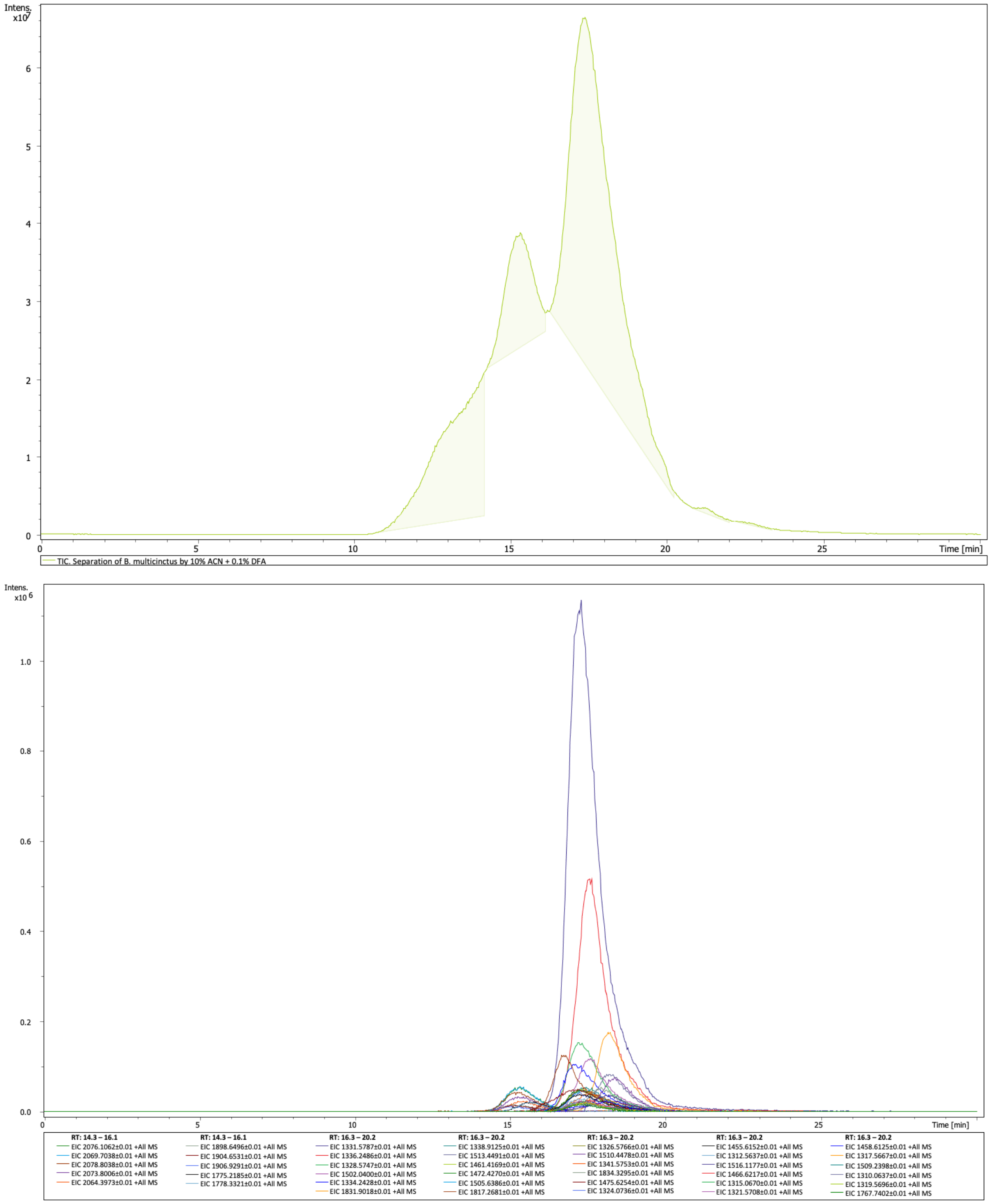


*Figure S20: TIC and EICs of SEC-MS measurement of B. multicinctus venom separated using 10% ACN + 0.1% DFA.*

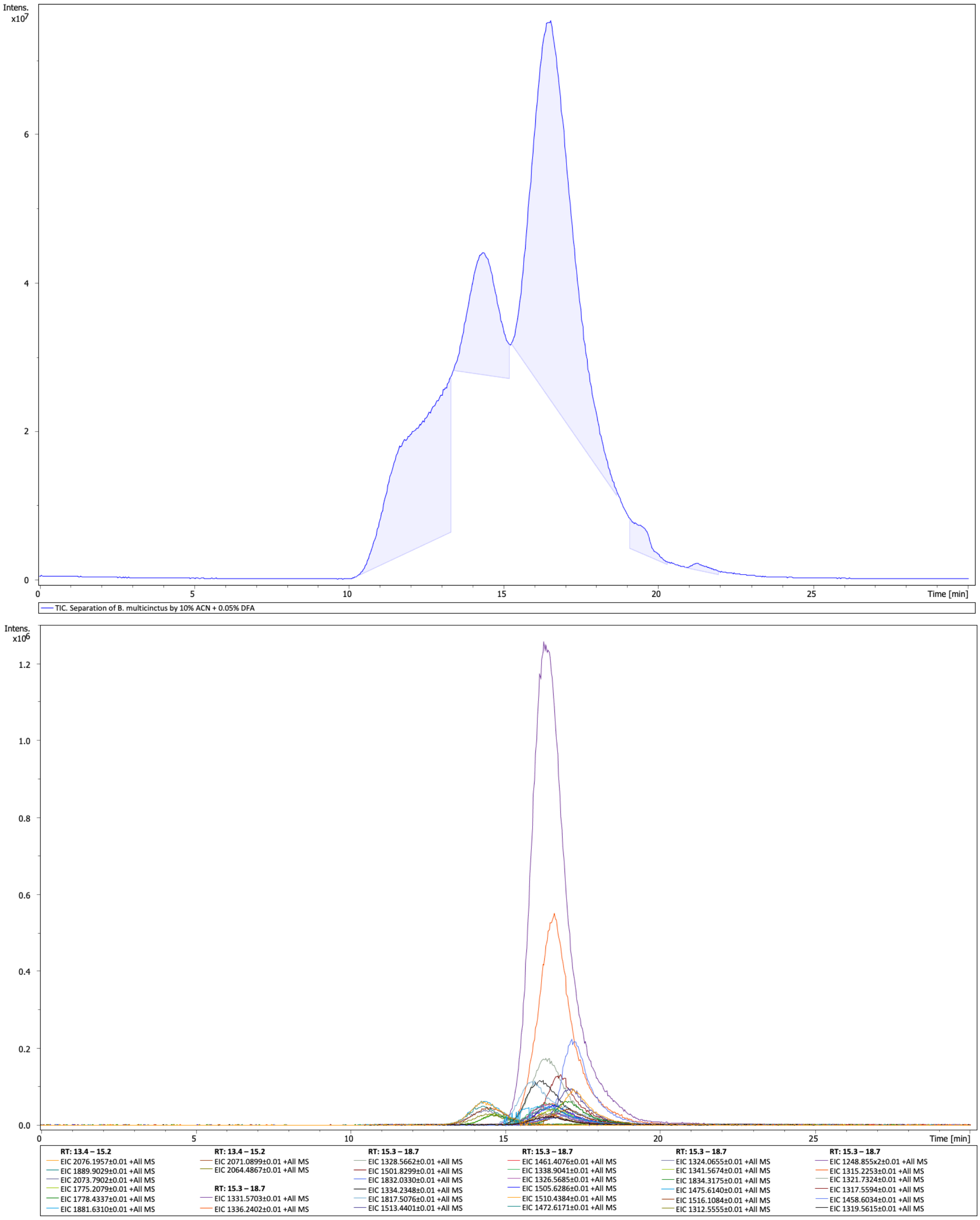


*Figure S21: TIC and EICs of SEC-MS measurement of B. multicinctus venom separated using 10% ACN + 0.05% DFA.*

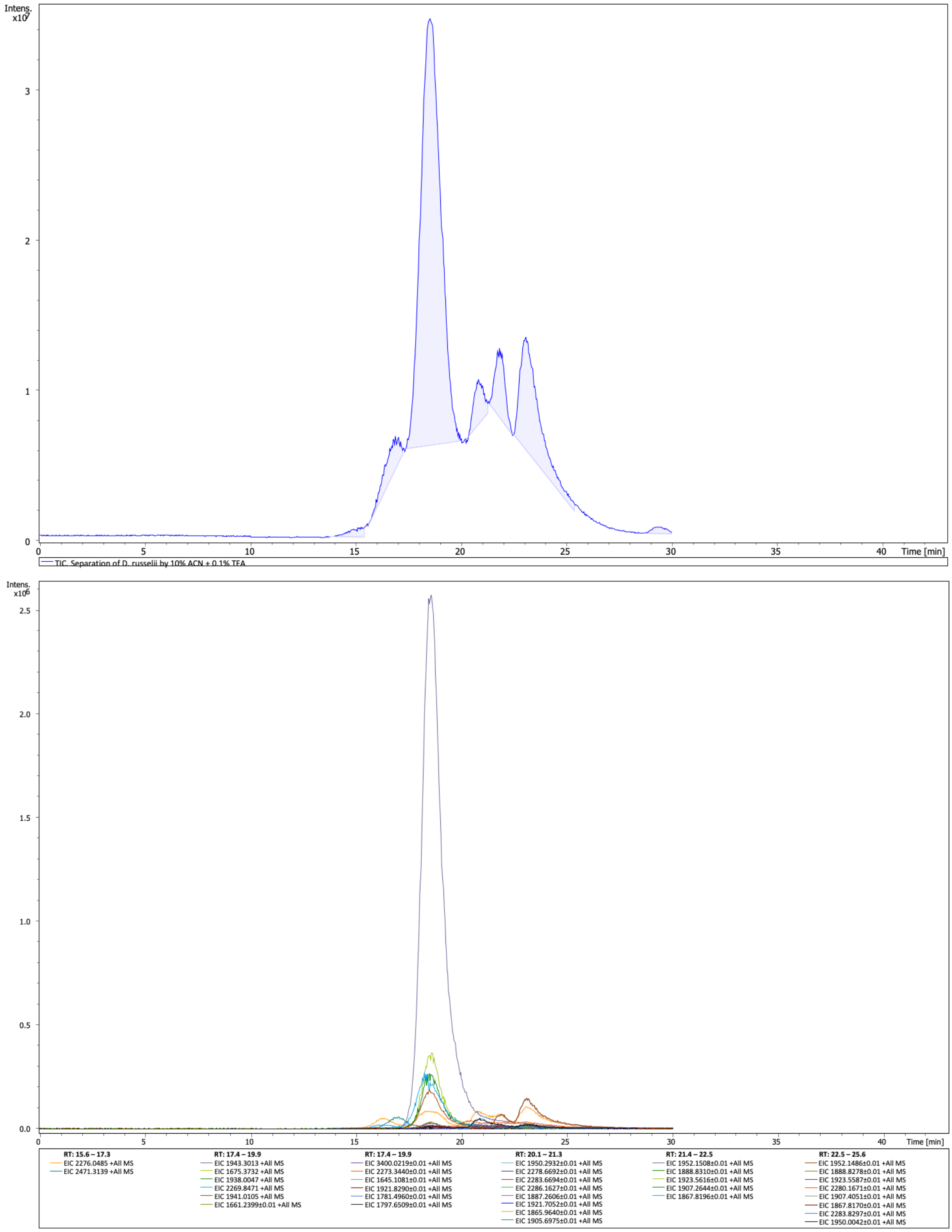


*Figure S22: TIC and EICs of SEC-MS measurement of D. russelii venom separated using 10% ACN + 0.1% TFA.*

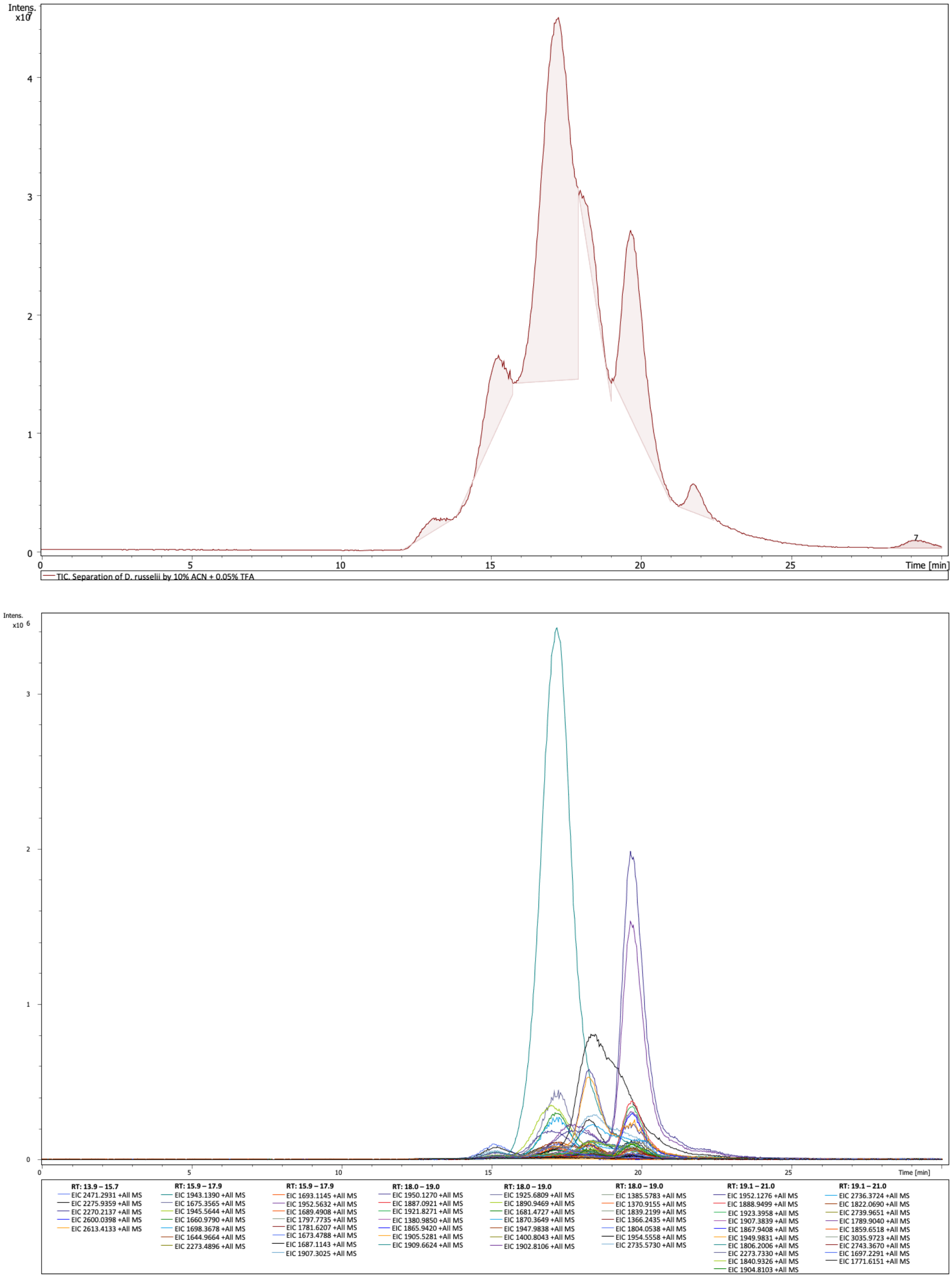


*Figure 23: TIC and EICs of SEC-MS measurement of D. russelii venom separated using 10% ACN + 0.05% TFA.*

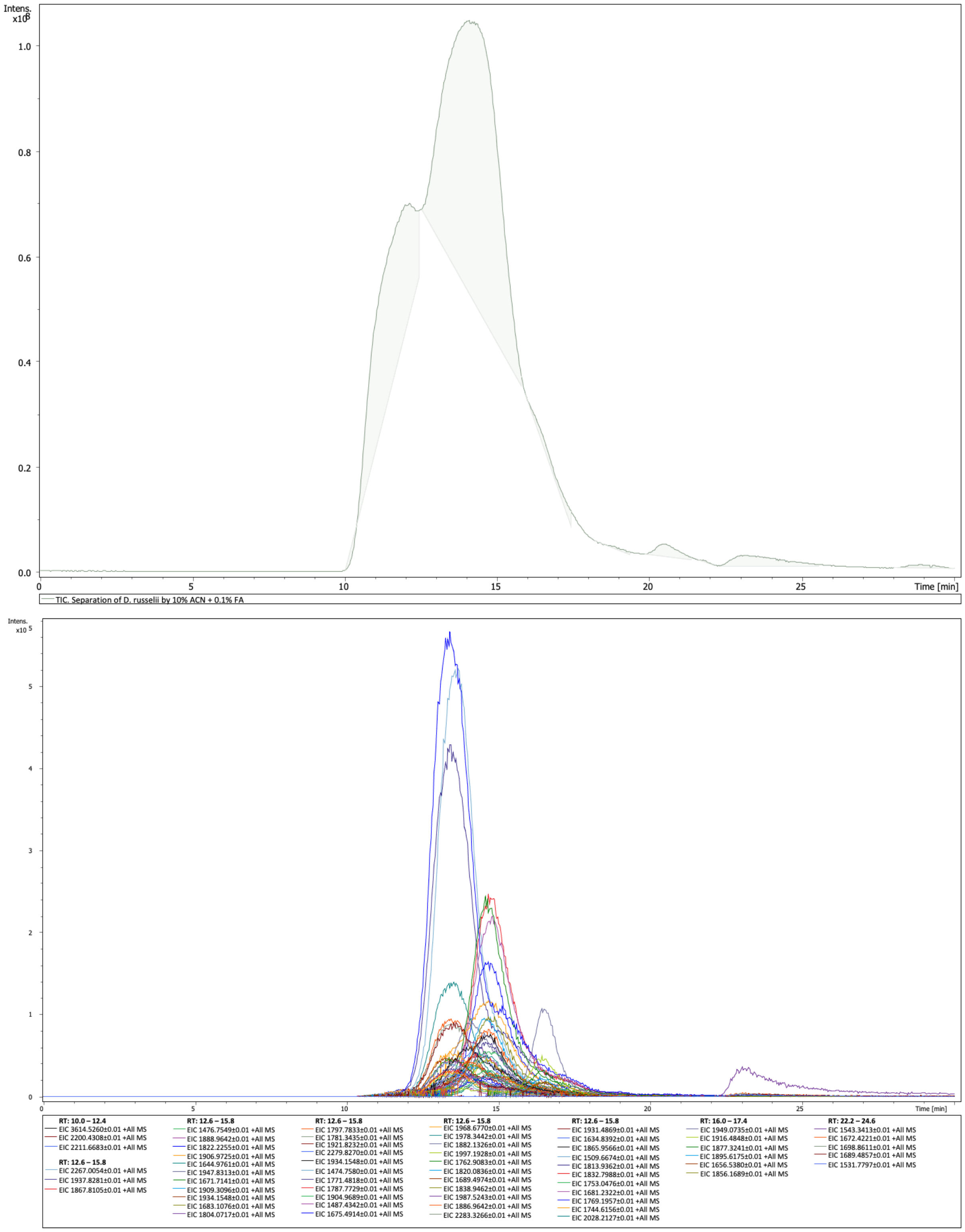


*Figure S24: TIC and EICs of SEC-MS measurement of D. russelii venom separated using 10% ACN + 0.1% FA.*

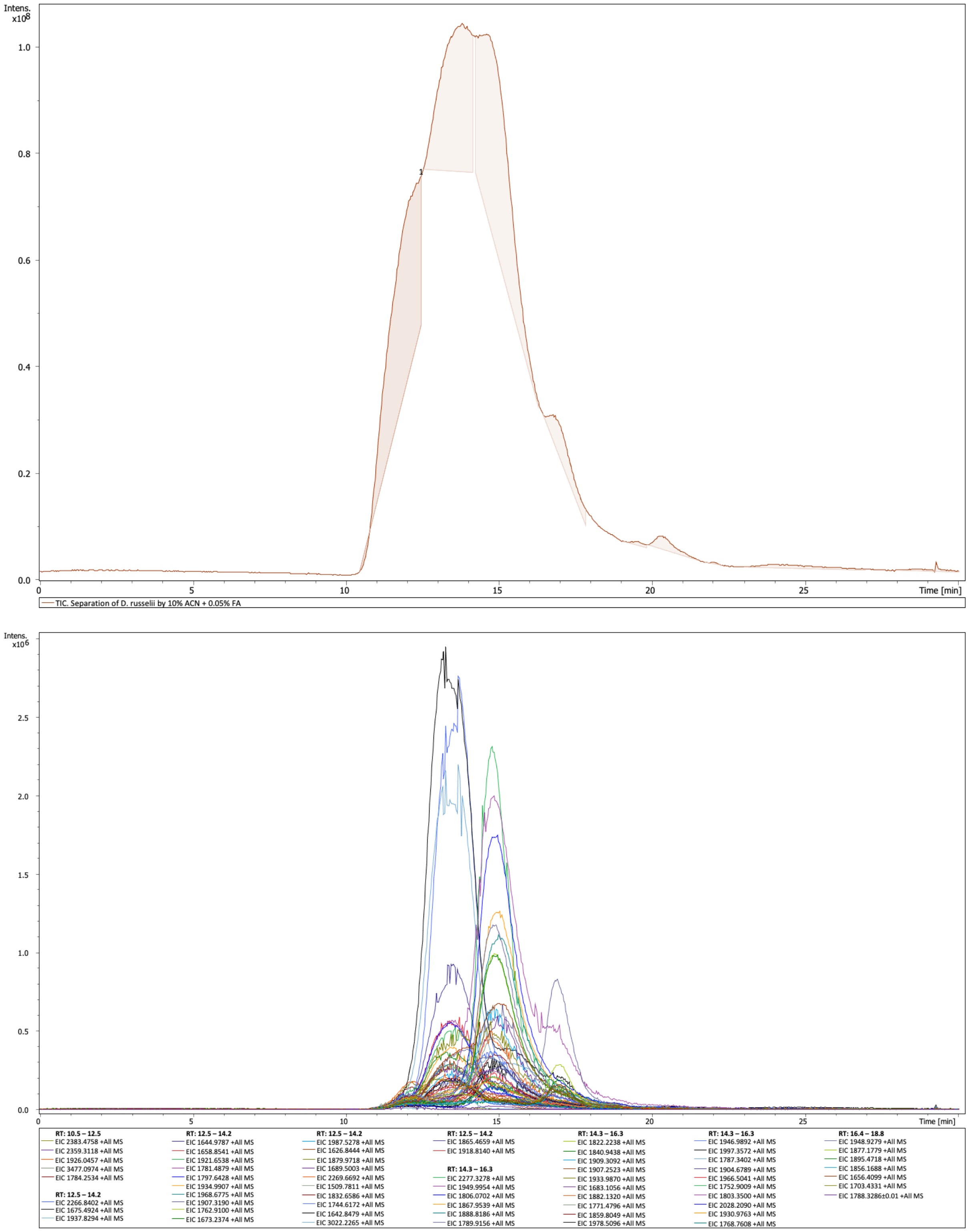


*Figure S25: TIC and EICs of SEC-MS measurement of D. russelii venom separated using 10% ACN + 0.05% FA.*

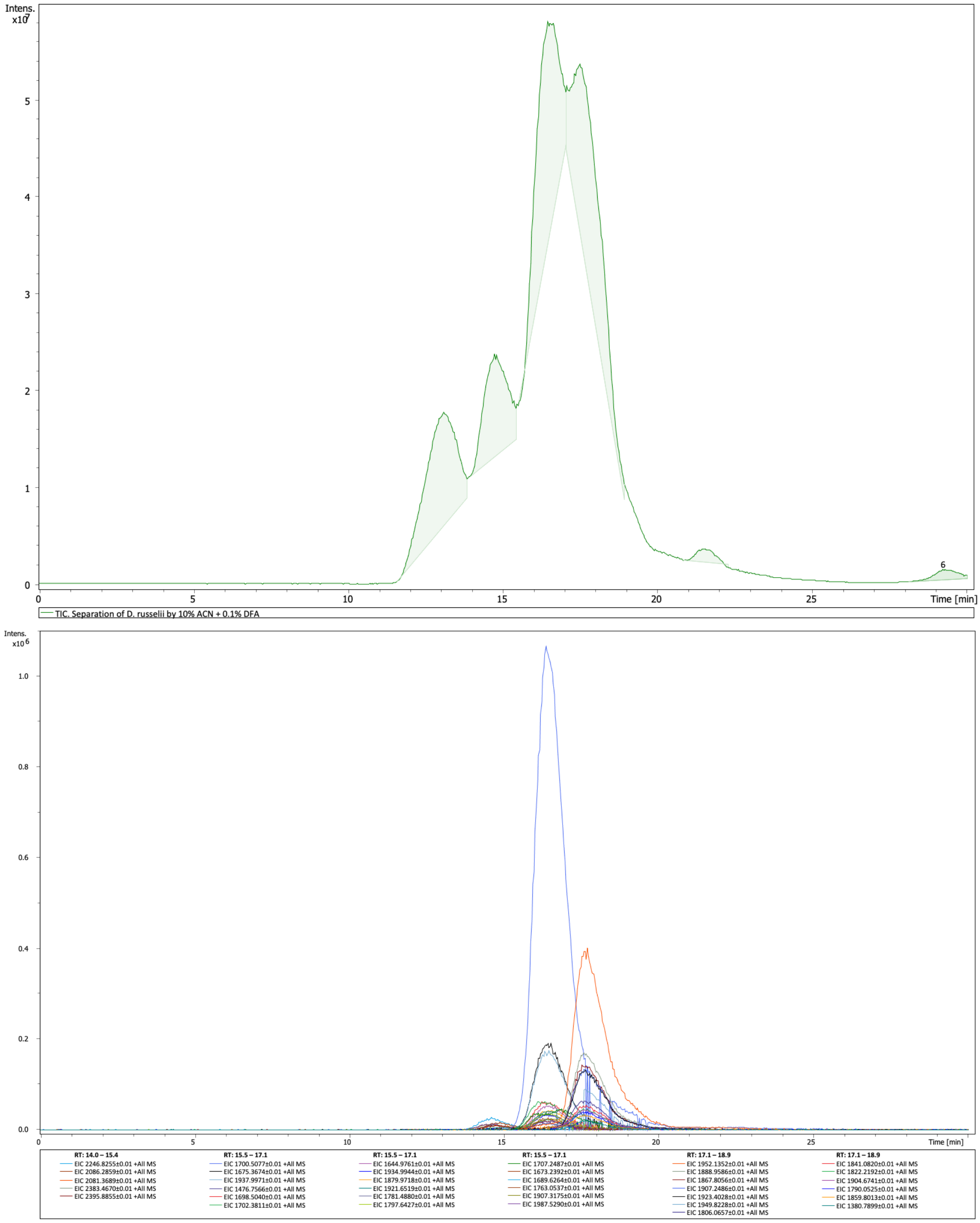


*Figure S26: TIC and EICs of SEC-MS measurement of D. russelii venom separated using 10% ACN + 0.1% DFA*

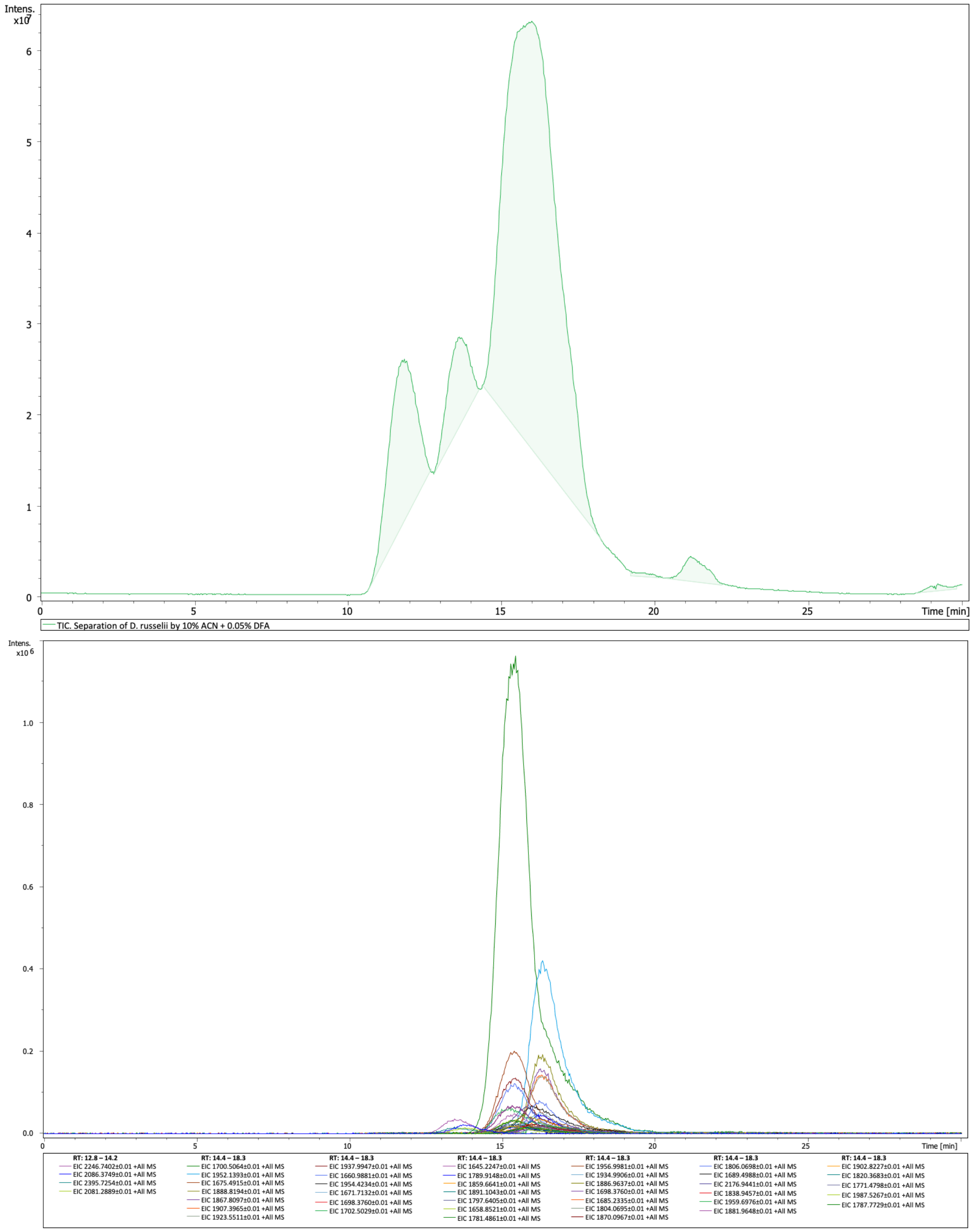


*Figure S27: TIC and EICs of SEC-MS measurement of D. russelii venom separated using 10% ACN + 0.05% DFA.*

# S8. Reference material


1. Still, K.B.M.; Nandlal, R.S.S.; Slagboom, J.; Somsen, G.W.; Casewell, N.R.; Kool, J. Multipurpose HTS Coagulation Analysis: Assay Development and Assessment of Coagulopathic Snake Venoms. *Toxins (Basel).* **2017**, *9*, 1–16, doi:10.3390/toxins9120382.
2. Xie, C.; Slagboom, J.; Albulescu, L.O.; Somsen, G.W.; Vonk, F.J.; Casewell, N.R.; Kool, J. Neutralising Effects of Small Molecule Toxin Inhibitors on Nanofractionated Coagulopathic Crotalinae Snake Venoms. *Acta Pharm. Sin. B* **2020**, *10*, 1835–1845, doi:10.1016/j.apsb.2020.09.005.
3. Mladic, M.; de Waal, T.; Burggraaff, L.; Slagboom, J.; Somsen, G.W.; Niessen, W.M.A.; Manjunatha Kini, R.; Kool, J. Rapid Screening and Identification of ACE Inhibitors in Snake Venoms Using At-Line Nanofractionation LC-MS. *Anal. Bioanal. Chem.* **2017**, *409*, 5987–5997, doi:10.1007/s00216-017-0531-3.
4. Xie, C.; Albulescu, L.-O.; Bittenbinder, M.A.; Somsen, G.W.; Vonk, F.J.; Casewell, N.R.; Kool, J. Neutralizing Effects of Small Molecule Inhibitors and Metal Chelators on Coagulopathic Viperinae Snake Venom Toxins. *Biomedicines* **2020**, *8*, doi:10.3390/biomedicines8090297.
5. Slagboom, J.; Mladić, M.; Xie, C.; Kazandjian, T.D.; Vonk, F.; Somsen, G.W.; Casewell, N.R.; Kool, J. High Throughput Screening and Identification of Coagulopathic Snake Venom Proteins and Peptides Using Nanofractionation and Proteomics Approaches. *PLoS Negl. Trop. Dis.* **2020**, *14*, 1–26, doi:10.1371/journal.pntd.0007802.
6. Lardeux, H.; Duivelshof, B.L.; Colas, O.; Beck, A.; McCalley, D. V.; Guillarme, D.; D'Atri, V. Alternative Mobile Phase Additives for the Characterization of Protein Biopharmaceuticals in Liquid Chromatography – Mass Spectrometry. *Anal. Chim. Acta* **2021**, *1156*, 338347, doi:10.1016/j.aca.2021.338347.
7. Mou, X.; Yang, X.; Li, H.; Ambrogelly, A.; Pollard, D.J. A High Throughput Ultra Performance Size Exclusion Chromatography Assay for the Analysis of Aggregates and Fragments of Monoclonal Antibodies. *Pharm. Bioprocess.* **2014**, *2*, 141–156, doi:10.4155/pbp.14.7.
8. Goyon, A.; Beck, A.; Colas, O.; Sandra, K.; Guillarme, D.; Fekete, S. Evaluation of Size Exclusion Chromatography Columns Packed with Sub-3 Mm Particles for the Analysis of Biopharmaceutical Proteins. *J. Chromatogr. A* **2017**, *1498*, 80–89, doi:10.1016/j.chroma.2016.11.056.
9. Goyon, A.; Fekete, S.; Beck, A.; Veuthey, J.L.; Guillarme, D. Unraveling the Mysteries of Modern Size Exclusion Chromatography - the Way to Achieve Confident Characterization of Therapeutic Proteins. *J. Chromatogr. B Anal. Technol. Biomed. Life Sci.* **2018**, *1092*, 368–378, doi:10.1016/j.jchromb.2018.06.029.
10. Castells, R.C.; Castells, C.B.; Castillo, M.A. Influence of Differences between Sample and Mobile Phase Viscosities on the Shape of Chromatographic Elution Profiles. *J. Chromatogr. A* **1997**, *775*, 73–79, doi:10.1016/S0021-9673(97)00343-9.
11. Gekko, K.; Ohmae, E.; Kameyama, K.; Takagi, T. Acetonitrile-Protein Interactions: Amino Acid Solubility and Preferential Solvation. *Biochim. Biophys. Acta - Protein Struct. Mol. Enzymol.* **1998**, *1387*, 195–205, doi:10.1016/S0167-4838(98)00121-6.
12. Mattos, C.; Ringe, D. Proteins in Organic Solvents. *Curr. Opin. Struct. Biol.* **2001**, *11*, 761–764, doi:10.1016/S0959-440X(01)00278-0.
13. Jiang, Y.; Li, Y.; Lee, W.; Xu, X.; Zhang, Y.; Zhao, R.; Zhang, Y.; Wang, W. Venom Gland Transcriptomes of Two Elapid Snakes (Bungarus Multicinctus and Naja Atra) and Evolution of Toxin Genes. *BMC Genomics* **2011**, *12*, 1–13, doi:10.1186/1471-2164-12-1.
14. Faisal, T.; Tan, K.Y.; Sim, S.M.; Quraishi, N.; Tan, N.H.; Tan, C.H. Proteomics, Functional Characterization and Antivenom Neutralization of the Venom of Pakistani Russell's Viper (Daboia Russelii) from the Wild. *J. Proteomics* **2018**, *183*, 1–13, doi:10.1016/j.jprot.2018.05.003.
15. Oh, A.M.F.; Tan, K.Y.; Tan, N.H.; Tan, C.H. Proteomics and Neutralization of Bungarus Multicinctus (Many-Banded Krait) Venom: Intra-Specific Comparisons between Specimens from China and Taiwan. *Comp. Biochem. Physiol. Part - C Toxicol. Pharmacol.* **2021**, *247*, 109063, doi:10.1016/j.cbpc.2021.109063.
16. Markland, F.S.; Swenson, S. Snake Venom Metalloproteinases. *Toxicon* **2013**, *62*, 3–18,

doi:10.1016/j.toxicon.2012.09.004.

17. Serrano, S.M.T.; Maroun, R.C. Snake Venom Serine Proteinases: Sequence Homology vs. Substrate Specificity, a Paradox to Be Solved. *Toxicon* **2005**, *45*, 1115–1132, doi:10.1016/j.toxicon.2005.02.020.
18. Lin, B.; Zhang, J.R.; Lu, H.J.; Zhao, L.; Chen, J.; Zhang, H.F.; Wei, X.S.; Zhang, L.Y.; Wu, X.B.; Lee, W.H. Immunoreactivity and Neutralization Study of Chinese Bungarus Multicinctus Antivenin and Lab-Prepared Anti-Bungarotoxin Antisera towards Purified Bungarotoxins and Snake Venoms. *PLoS Negl. Trop. Dis.* **2020**, *14*, 1–19, doi:10.1371/journal.pntd.0008873.
19. Ye, F.; Zheng, Y.; Wang, X.; Tan, X.; Zhang, T.; Xin, W.; Wang, J.; Huang, Y.; Fan, Q.; Wang, J. Recognition of Bungarus Multicinctus Venom by a DNA Aptamer against β-Bungarotoxin. *PLoS One* **2014**, *9*, 2–9, doi:10.1371/journal.pone.0105404.
20. Burke, J.E.; Dennis, E.A. Phospholipase A2 Biochemistry. *Cardiovasc. Drugs Ther.* **2009**, *23*, 49–59, doi:10.1007/s10557-008-6132-9.
21. Fry, B.G.; Wüster, W.; Kini, R.M.; Brusic, V.; Khan, A.; Venkataraman, D.; Rooney, A.P. Molecular Evolution and Phylogeny of Elapid Snake Venom Three-Finger Toxins. *J. Mol. Evol.* **2003**, *57*, 110–129, doi:10.1007/s00239-003-2461-2.
